# Comparison of methods to detect copy number alterations in cancer using simulated and real genotyping data

**DOI:** 10.1186/1471-2105-13-192

**Published:** 2012-08-07

**Authors:** David Mosén-Ansorena, Ana María Aransay, Naiara Rodríguez-Ezpeleta

**Affiliations:** 1 Genome Analysis Platform, CIC bioGUNE - CIBERehd, Technologic Park of Bizkaia, building 502, 48160, Derio, Spain; 2 Marine Research Division, AZTI-Tecnalia, Txatxarramendiugartea z/g, 48395, Sukarrieta, Spain

## Abstract

**Background:**

The detection of genomic copy number alterations (CNA) in cancer based on SNP arrays requires methods that take into account tumour specific factors such as normal cell contamination and tumour heterogeneity. A number of tools have been recently developed but their performance needs yet to be thoroughly assessed. To this aim, a comprehensive model that integrates the factors of normal cell contamination and intra-tumour heterogeneity and that can be translated to synthetic data on which to perform benchmarks is indispensable.

**Results:**

We propose such model and implement it in an R package called CnaGen to synthetically generate a wide range of alterations under different normal cell contamination levels. Six recently published methods for CNA and loss of heterozygosity (LOH) detection on tumour samples were assessed on this synthetic data and on a dilution series of a breast cancer cell-line: ASCAT, GAP, GenoCNA, GPHMM, MixHMM and OncoSNP. We report the recall rates in terms of normal cell contamination levels and alteration characteristics: length, copy number and LOH state, as well as the false discovery rate distribution for each copy number under different normal cell contamination levels.

Assessed methods are in general better at detecting alterations with low copy number and under a little normal cell contamination levels. All methods except GPHMM, which failed to recognize the alteration pattern in the cell-line samples, provided similar results for the synthetic and cell-line sample sets. MixHMM and GenoCNA are the poorliest performing methods, while GAP generally performed better. This supports the viability of approaches other than the common hidden Markov model (HMM)-based.

**Conclusions:**

We devised and implemented a comprehensive model to generate data that simulate tumoural samples genotyped using SNP arrays. The validity of the model is supported by the similarity of the results obtained with synthetic and real data. Based on these results and on the software implementation of the methods, we recommend GAP for advanced users and GPHMM for a fully driven analysis.

## Background

Two of the genetic instabilities associated with cancer are copy number alterations (CNAs) and loss of heterozygosity (LOH) events. Both are distinctive features of tumoural cells, and their acquisition has been reported to affect the expression of oncogenes and tumour-suppressor genes [1]. Hence, the detection and characterization of both CNAs and LOH in tumoural samples is crucial to identify candidate cancer-related genes, as well as to discriminate cancer types [2] and to understand tumour initiation and complexity [3].

Single nucleotide polymorphism (SNP) arrays of Illumina [4] and Affymetrix [5] platforms allow screening for such alterations at high resolution and throughout the whole genome, providing measures of copy number changes and allelic ratio. Namely, the log R ratio (LRR) reflects the total intensity signals for both alleles, and the B allele frequency (BAF) is the relative proportion of one of the alleles with respect to the total intensity signal. Because they provide complementary information, both LRR and BAF signals are required for a complete characterization of copy number changes and allelic ratio. Yet, although each combination of copy number and allelic ratio has an expected LRR value and a specific BAF band pattern, these can be distorted by experimental probe-specific noise and by autocorrelated [6] and dye [7] biases, respectively.

In the case of tumour samples, three additional issues need to be considered. First, tumour genomes contain numerous altered regions whose copy number is different from two, making the genotypes in nearly the whole genome non-diploid. Under this situation of altered DNA index (i.e., half of the mean copy number), the LRR baseline level is shifted and needs to be estimated. Because this estimation affects copy number assignment [8,9], equally likely results with different biological interpretations are possible. Second, tumour biopsies can be contaminated with normal cells whose genotypes are mainly diploid. This causes the LRR and BAF signals to shrink and converge towards those of a diploid state proportionally to the degree of contamination [10]. Third, tumours can be composed of subclones, this is, subpopulations of cells that harbour specific alterations along with the shared ones, which makes LRR and BAF signals even more complex [11].

Therefore, inferring relevant information such as breakpoint location, copy number state and genotype from tumour samples requires sophisticated mathematical models and computer programs that take general and tumour specific factors into account. There are several methods for automatic CNA and LOH detection in unpaired tumour samples on SNP arrays. Here we focus on those available for the Illumina platform, more abundant than those for Affymetrix: OncoSNP [9], GenoCNA [12], GPHMM [13], MixHMM [14], ASCAT [15] and GAP [8] (see Table 1). The first four are based on hidden Markov models (HMM) whereas the latter two are based on a segmentation procedure followed by ploidy and normal cell contamination estimation, which allow correct segment calling. Other methods, namely PSCN [16] and OverUnder [17], are not considered here, because the former requires matched non-tumoural samples and the latter is not available as a stand-alone software. For a comparison of methods with the Affymetrix platform, we refer to Rasmussen et al. [18]. 

**Table 1:** Characteristics of the six assessed methods

	**MixHMM**	**GenoCNA**	**GPHMM**	**OncoSNP**	**ASCAT**	**GAP**
User-definable noise levels	Yes	No	No	No	No	Yes
User-definable LRR copy number means	Yes	No	No	Yes	No	Yes
Automatic LRR shift estimation	No	No	Yes	Yes	Yes	Yes
Automatic contamination estimation	No	Yes	Yes	Yes	Yes	Yes
Germline and somatic LOH distinction	No	No	No	Yes	No	Yes
Maximum copy number	5	4	7	8	?	8
Computation time	Low	Medium	Low	High	Low	Medium

HMM-based methods infer the most likely succession of genotypes (including copy number and allele distribution) given the LRR and BAF values under the assumption of certain conditions such as the expected LRR means for each copy number and the population B allele frequency (PFB) of each SNP. Sample-wide parameters, such as LRR baseline shift, standard deviation of the noise in both signals and proportion of normal cells, are unknown and typically optimized using expectation-maximization (EM) algorithms. The four aforementioned HMM-based methods differ on which parameters are optimized and how the EM is performed, as well as on the definition of the HMMs, including state characterization.

GAP is based on pattern recognition of a segmented and smoothed bi-dimensional profile. The method is implemented as a three-step workflow: (1) tQN (thresholded quantile normalization) normalization [7] for symmetrisation of BAF signal; (2) extraction of germline LOH regions, transformation of BAF into a unimodal symmetric signal (mBAF), segmentation of LRR and mBAF signals, and merging of LOH germline regions breakpoints with segmentation breakpoints; and (3) local copy number assignment through pattern recognition in the bi-dimensional LRR-BAF space. ASCAT transforms BAF into a unimodal symmetric signal, similar to mBAF, and performs a bivariate segmentation of LRR and the BAF transformation. Then, ASCAT assigns to each region the allele copy numbers that better fit the data, based on the ploidy that maximizes a reliability score.

Previous studies have compared some of these methods among themselves [9,13] and against non-tumour-specific methods [12,14], but so far no systematic assessment of the performance of these recently developed CNA and LOH detection approaches has been performed. Therefore, we have developed a comprehensive model that integrates normal cell contamination and intra-tumour heterogeneity to synthetically generate data that mimics tumoural samples on which to perform benchmarks. Using this synthetic data with a wide range of alteration characteristics and normal cell contamination levels, and a dilution series of a breast cancer cell-line [10], we have compared the performance of the six currently available software that consider normal cell contamination and rely on both BAF and LRR to detect CNAs and LOHs from unpaired samples on Illumina SNP arrays.

## Methods

### The model

The model presented here draws from a model described by Yau et al. [9] that has been extended to integrate normal cell contamination and tumour heterogeneity. Furthermore, with the aim of adapting it to the generation of realistic tumour-like synthetic data, terms for known biases, generation restrictions and alteration variability have been included.

The LRR signal for a locus *i* is modelled as:

(1) $$lrr_{i}=\sum_{j=1}^{J}w_{i,j}r_{x_{\mathrm{ij}}}+l+c_{i}$$

Where *J* is the number of cell types (one for normal cells plus *J*–1 tumour cell types with different copy numbers at locus *i*., *W*_*i,j*_ is the proportion of the *j*-th cell type,$r_{x_{i,j}}$ is the expected LRR value for the copy number *x* of the *j*-th cell type present at locus *i*, the real number *l* is the sample-wide baseline shift, and *c* is an autocorrelated bias that simulates the background noise due to biological features that are not corrected for in the design of the array or in the detection methods.

In the equation above, the cell type proportions are non-null and sum one, and the value *W*_1_ is constant sample-wide given that normal contamination does not change from probe to probe:

(2) $$w_{i,j}\in (0,1]\wedge \sum_{j=1}^{J}w_{i,j}=1\wedge \forall s,t\left(w_{s,1}=w_{t,1}\right)$$

the value *r*_*x*_ draws from a Gaussian distribution with a mean *u*_*x*_, which reflects the expected LRR value for copy number *x*, and a standard deviation σ_*x*_, which can be different for each *x*:

(3) $$r_{x}\sim N\left(\mu_{x},\sigma_{x}\right)$$

Finally, the autocorrelated bias *c* follows a Box-Jenkins (i.e. ARMA) model:

(4) $$c_{i}=ARMA{\left(\rho ,\tau \right)}_{i}$$

The BAF signal for a locus *i* is modelled as:

(5) $$baf_{i}=\frac{\sum_{j=1}^{J}w_{i,j}z_{i,j}}{\sum_{j=1}^{J}w_{i,j}x_{i,j}}+n_{i}$$

Where *z*_*i,j*_ is the number of B alleles of the *j*-th cell type in the locus *i* out of the total copy number *x*_*i,j*_, and *n*_*i*_ is the noise of the BAF signal, modelled as a normal for heterozygous values, and as a mixture of a half-normal and a point mass function (0 in *M*_0_ and 1 in *M*_1_) for homozygous values:

(6) $$P\left(n_{i}\text{|}b_{i}\right)=I_{\left\{b_{i}=0\right\}}\left[\pi N_{+}\left(0,\sigma_{baf\_ho}\right)+\left(1-\pi \right)M_{0}\right]+I_{\left\{b_{i}=1\right\}}\left[\pi N_{-}\left(0,\sigma_{baf\_he}\right)+\left(1-\pi \right)M_{1}\right]+I_{\left\{0<b_{i}<1\right\}}N\left(0,\sigma_{baf\_ho}\right)$$

Here, *I* is the indicator function and *π* is the proportion of BAF values that are forced to take the extreme homozygous values zero or one. The standard deviation σ_*baf_ho*_ of homozygous values is set as a separate parameter from σ_*baf_he*_, given that it is usually observed to be lower.

The relationship between *x*_*i*,1_ = 2 and *z*_*i*,1_, thus in normal cells, is described by a binomial distribution:

(7) $$z_{i,1}=B\left(x_{i,1},p_{i}\right)$$

where *p*_*i*_ is the population B allele frequency (PFB) for the SNP captured by the *i*-th probe. In turn, tumour cells will necessarily be homozygous for a locus *i* if normal cells are homozygous. On the other hand, if normal cells are heterozygous, the number of B alleles *z* is bound by *x*:

(8) $$z_{i,1}\in \left\{0,x_{i,1}\right\}\to \forall j\left(z_{i,j}=x_{i,1}\right)j\in \left\{\text{1…}J\right\}$$

(9) $$z_{i,1}=1\to \forall j\left(0\leq z_{i,j}\leq x_{i,j}\right)j\in \left\{\text{1…}J\right\}$$

Additionally, there is a coherence restriction that applies to SNPs belonging to the same region. The number of B alleles of a SNP *t* should be the same to the number of B or A alleles of any other SNP *s* within the same region:

(10) $$\forall s,t\left(z_{t,j}\in \left\{z_{s,j},x_{s,j}-z_{s,j}\right\}\right)j\in \left\{\text{1…}J\right\}$$

The reason is that different alteration events yield certain possible genotype combinations. For example, given a diploid genotype AB, a CN4 (i.e. copy number 4) AABB genotype would not be coherent with a triplication of one of the alleles, but AAAB and ABBB would.

### CnaGen

We implemented the described model as an R package whose purpose is the generation of synthetic SNP genotyping data. The software, named CnaGen, is available at http://web.bioinformatics.cicbiogune.es/cnagen and has fully customizable parameters as detailed below.

CnaGen allows to generate a broad range of region types combining different copy numbers, presence or not of somatic or germline LOH and spanning different number of SNPs per region. Furthermore, regions with intra-tumour complexity can also be generated establishing the copy number per subclone and the proportion of the major subclone. Regions can be combined in samples by establishing the region types to be present and the number of occurrences of each region type, and by defining the degree of normal cell contamination per sample. Other parameters, such as those defining LRR baseline shift, autocorrelated bias, expected LRR per copy number and noises in the LRR and BAF signals, are also user-definable.

As an alternative to fully specifying region characteristics, the user can provide a scaffold that contains them (length, copy number, allelic ratio and presence of germline LOH). Such scaffold, which implicitly holds the distribution of genomic rearrangements, may be obtained from previous experiments, so we regard this alternatively generated data as hybrid between synthetic and real. In this scenario, additional user-definable genome-wide properties are: long-distance genomic waves and overall level of intra-tumour complexity.

The following options to establish PFBs are available in CnaGen: (i) sampling from B allele frequencies in Caucasian populations of SNPs in the Illumina *Human660W-Quad* array; (ii) sampling from a uniform distribution; (iii) a constant *p*_*i*_=0.5, which maximizes genotype information; (iv) any other constant *p*_*i*_; (v) and a three-peak distribution at 0, 0.5 and 1, which approximates PFBs in the human genome.

### Generation of synthetic samples

In order to assess the performance of each method under different conditions, we generated synthetic SNP genotyping sample sets with CnaGen. The introduced statistical framework models the data observation process, which allows the assessment of the factor it comprises: copy number with the possibility of intra-tumour heterogeneity, presence of LOH, length of region and degree of normal cell contamination. Because the combinatorial space is too large to be explored exhaustively, subsets of values for each factor were selected. To test for the combined effect of number of copies, presence of LOH and length of alteration, fragments with copy numbers 1, 2, 3, 4 and 5 with and without somatic or germline LOH spanning 10, 20, 40, 80 or 160 SNPs were generated. Although longer aberrations, which may even comprise whole chromosomal arms, are typically found in tumoural cells, method performance does not change significantly beyond the longest considered length, specially under low normal cell contamination levels, as shown in the Results section. To test for the effect of different levels of normal cell contaminations, four percentages (0, 25, 50 or 75%) of non-tumoural cells were considered.

**Figure 1:**
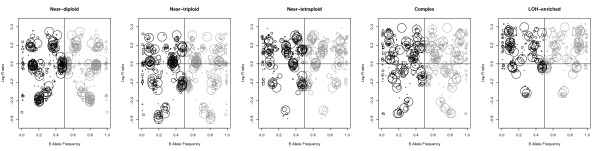
**Characteristic tumour alteration patterns.** Depiction of five tumoural samples with characteristic alteration patterns. Each circle represents the mean LRR (y-axis) and BAF (x-axis) values of a specific region. Circle size represents the length of the corresponding region. The samples present 25% normal cell contamination. For the computation of BAF means, the signal was mirrored along the 0.5 axis and removed from homozygous SNPs in heterozygous regions. Grey circles are simply a mirror from the computed black circles.

The latent genomic rearrangement process of tumorigenesis was recreated in CnaGen by generating samples that mimic characteristic tumoural alteration patterns. We chose five typical patterns (Figure 1, Additional file 1 for the code to generate the samples): near-diploid (DNA index 1.03, 45.4% CN2 regions), near-triploid (DNA index 1.32, 40.3% CN3 regions), near-tetraploid (DNA index 1.57, 38.3% CN4 regions), LOH-enriched (DNA index 1.31, 40.1% LOH regions) and a complex pattern with great intra-tumour complexity (DNA index 1.39, 47.6% complex regions). One hundred replicates were generated for every combination of alteration pattern and considered contamination level, having each replicate between 205 and 280 fragments that cover the range of considered copy numbers, lengths and LOH status.

The rest of the parameters were set as follows: the LRR baseline shifts were drawn from Gaussian distributions with means established based on the correlation between DNA index and baseline shift (see "Parameter relationships" subsection below). In the Box-Jenkins model, the orders of the autoregressive and moving-average processes (i.e. *p* and *τ* ) were selected so that the resulting autocorrelated noise resembles the genomic curves found by Diskin et al. [6]. Expected means, *μ*_x_, for each copy number *x* were established as half of those values specified in [8] and approach those in the models of the methods evaluated. PFB values were drawn from those of the SNPs present in the Illumina *Human660W-Quad* array in Caucasian populations, and 30% of the homozygous probes were forced to take a zero or one value in the BAF signal in order to resemble real data. Finally, Gaussian noises were set to *σ*_*x*_=0.2 , *σ*_*baf_he*_=0.3 and *σ*_*baf_ho*_=0.15, being the former two similar to those HER2-positive samples of high quality analyzed by Li et al. [13]; and the noise for the homozygous SNPs in the BAF signal was set to half of the noise of the heterozygous SNPs, based on our own observations.

### Cell-line

As a complimentary evaluation dataset, we used the dilution series of the CRL-2324 breast cancer cell line, genotyped with Illumina *370k BeadChips* by Staaf et al. [10] where genomic DNA of breast carcinoma cells were mixed with DNA from lymphoblastoid cells at known proportions. This dataset has also been used by other authors [8,9,13] to assess the self-consistency of their methods.

In this dataset, the BAF and LRR signal noises range from 0.02 to 0.03 and from 0.18 to 0.25, respectively. These values are considered good in the case of BAF and on the limit for a sensitive analysis in the case of LRR [19]. GC content bias was found to be between 0.016 and 0.042, which is satisfactory [13]. Chromosomes 6 and 16 were removed from the analysis due to long heterozygous deletions in the lymphoblastoid cells [8,9,13] and sex chromosomes were not included, due to differences in how they are handled by the assessed methods.

### Preprocessing

Because GAP, GenoCNA and MixHMM do not integrate GC content biases into their models, the GC reduction model from the PennCNV package [19] was applied before running these programs on cell-line data. tQN [7], which accounts for dye bias, was only applied in GAP, given that it is part of the method workflow. Similarly, GenoCNA parameters are already adapted to signal asymmetry [12], avoiding the need for such normalization. In turn, no preprocessing was applied to the synthetic data, as it was generated without the known GC and dye biases, and GPHMM and OncoSNP, which do integrate the former, were fed a GC model with constant GC content values.

### Assessment

On synthetic data, a region overlapping approach was taken to define recalls and false discovery rates (FDR). An altered region is considered to be recalled when a call with the same copy number overlaps at least half of the length of such region. Other fractions were considered, but the difference in the results is small, as most calls overlap at least a 95% (not shown). In turn, due to the high density of alterations in the synthetic data, and tumoural samples in general, calls are likely to span more than one actual altered region, rendering the FDR an unsuitable measure for regions. Therefore, we used an approximation that consists on defining FDR as the fraction of alterations called with the wrong copy number. Methods with better breakpoint detection yield higher FDRs with such approach, so FDR are not comparable among methods. However, it is useful because it provides insight into how wrong calls are distributed among copy numbers and to what extend methods reduce the number of alteration calls as normal cell contamination increases.

On cell-line data, the set of alterations and their boundaries are unknown, so we evaluated the possibility of using self-consistency with respect to pure tumour calls in order to measure performance. However, we reckon that such measure does not reflect reality if the best performance is not expected to be good. It was thus decided to use a gold standard set of alterations, defined as a manually selected subset of alterations in the pure tumour sample detected by the best performing method over synthetic data. While on synthetic data we considered copy numbers up to 5, in the case of cell-line data, CNAs with copy number equal or greater than 4 were grouped [13] because of the limitations of GenoCNA and the uncertainty in high copy numbers of the gold standard set.

For the computation of recall rates and FDRs, normal diploid (i.e. heterozygous copy number 2) fragments were excluded from all method calls and the reference region sets in both the synthetic and real data assessments.

## Results and discussion

### LRR and BAF patterns in synthetic data

**Figure 2:**
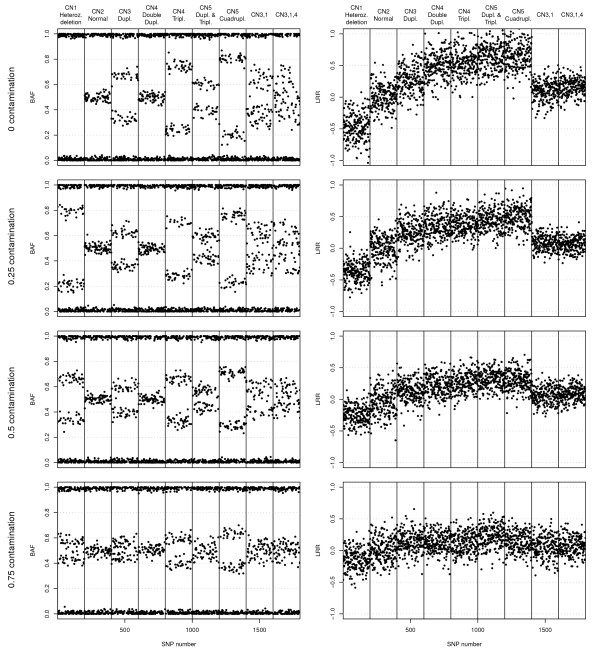
**Synthetic regions.** BAF (left graphs) and LRR (right graphs) signals of some example synthetic regions generated with CnaGen at different normal cell contamination levels: a heterozygous deletion (first column), a normal diploid region (second column), the various heterozygous CNA events up to copy number 5 (third to seventh columns) and two concrete cases of 2 and 3-subclone CNAs (last two columns). Each SNP probe provides a measurement of the proportion of one of the alleles (BAF) and the total intensity coming from the two alleles (LRR). Additional file 2 contains the same figure with noiseless data, so that BAF subclone bands can be seen clearly.

Figure 2 shows the LRR and BAF signals of some of the generated region types by CnaGen at different contaminations levels: a heterozygous deletion (i.e. copy number 1 alteration), a normal diploid region, the various heterozygous CNA events up to copy number 5 and two concrete cases of 2 and 3-subclone CNAs. Specifically, the subclones in the latter present either homozygous deletion (25%), allele duplication (50%) or allele triplication (25%). Although allelic ratio is not among the studied factors, Figure 2 reflects that generated data bears the different imbalances in mind.

In general, the different copy numbers can easily be distinguished on both signals under null contamination, but, as contamination increases, BAF heterozygous bands converge towards 0.5 and LRR levels converge towards zero (LRR shift is disregarded for the sake of clarity). Given the same allelic imbalance pattern of the diploid region and the CNA with double duplication, the only way to distinguish them is the LRR signal, evidencing the need of these two signals for a correct genotype inference. The selected copy numbers and subclone proportions for the depicted complex CNAs yield rather simple band patterns in comparison to most cases. Even so, their BAF signals contain 4 and 6 heterozygous bands, which are so close that they blur into a single wide band even under low normal cell contamination. Besides, under high contamination levels, similar copy numbers are undistinguishable on the LRR signal. Only under unreal conditions of zero probe-specific and autocorrelated noises would some of these scenarios still be distinguishable in both signals (see Additional file 2).

### Performance on synthetic samples

**Figure 3:**
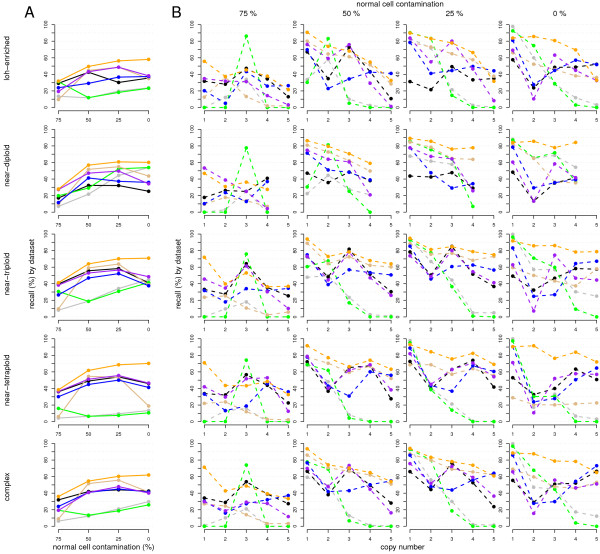
**Recall rates by method, contamination, and alteration copy number and length.** (**A**) Recall rates (y-axis) of each of the assessed methods, calculated by contamination over each of the 5 synthetic sample sets. Colour code: GAP (orange), updated GAP (golden), ASCAT (purple), GPHMM (black), OncoSNP (blue), GenoCNA (green), MixHMM (grey). (**B**) Recall rates (y-axis) of each of the assessed methods, calculated by contamination and alteration copy number over each of the 5 synthetic sample sets. Colour code: GAP (orange), updated GAP (golden), ASCAT (purple), GPHMM (black), OncoSNP (blue), GenoCNA (green), MixHMM (grey).

To determine the effect of the different factors tested in each method's performance, recall rates were plotted against the different values tested for copy number and length (see Figure 3 and Additional file 3). Graphs were grouped by sample pattern and normal cell contamination level. Recall of LOH status was assessed by regarding correct calls as those that matched not only copy number but also LOH status (lack or presence of LOH, regardless of whether germline or somatic), and similar graphs were generated under this criterion (Additional file 4). Methods tested include an updated version of GAP released in September, 2011 (named "updated GAP" in the following). See Additional file 5 for specifications and parameters used on each method.

In general, normal cell contamination works against recall ability, but ASCAT, GPHMM and OncoSNP seem to perform better when there is some degree of contamination (Figure 3). In the case of ASCAT, the reason is that segmentation has low breakpoint sensitivity under null contamination, because the adequate number of breakpoints depends on the segmentation goodness of fit, which is better for lower normal cell contamination levels. Thus, except at heavy normal cell contamination levels, ASCAT outputs less breakpoints for a concrete sample if it contains less normal cells.

Tumoural cells typically present a DNA index greater than 1, and the expected LRR baseline value decreases below zero with the decreasing proportion of normal cells. Therefore, methods that keep the baseline fixed at zero will tend to assign lower copy numbers than the real ones under low normal cell contamination levels, and viceversa. This is the case for GenoCNA and MixHMM, which tend to make copy number 1, 2 and 3 calls under low, medium and high contamination levels respectively (Figure 3B). The lack of baseline shift estimation, and thus of ploidy, makes them perform poorly under any condition that moves away from near-diploidy and little contamination. However, although their performance is similar under little contamination, GenoCNA improves with respect to MixHMM as contamination increases, because the former does estimate normal cell contamination, in contrast to the latter. The rest of the methods estimate both LRR baseline shift and normal cell contamination, and present similar performance over the different copy numbers, except for their exceptional recall rates for copy number 1 alterations and the fact that they tend to make few copy number 2 calls and prefer to assign regions copy numbers 1 and 3. GPHMM presents another exception to such trend. It has the most stable performance over different contamination levels, which is coherent with the results reported by Li et al. However, such stability and self-consistency arises from its tendency to make copy number 3 calls, a preference that is shared with ASCAT. This is visible if we separate recall rates by copy number and also if we look at the overall performance by dataset, as they perform better over samples with more copy number 3 alterations, specially near-triploid samples.

As expected, recall rates are higher for longer regions (Additional file 4). At low normal cell contamination levels, recall rates do not seem to improve much for regions beyond 160 SNPs in length but, at 75% contamination, regions need to be longer in order to be called correctly. Yet, given the observed trend in recall rates by length, we do not expect recall rates to significantly vary beyond lengths of a few hundred SNPs. The greater difference is seen on shorter regions, where GAP is more sensitive than the rest of the methods.

GAP is by far the best performer under the range of tested conditions and alterations. One of the advantages of GAP is that it puts breakpoint sensitivity before specificity. Then, it merges similar segments if necessary. This way, it is less probable for changes in mean to be missed. Additionally, ploidy estimation based on the whole segmented data, a feature that GAP shares with ASCAT, proves to give better results than the expectation maximisation performed by their HMM-based counterparts. Surprisingly, the updated version of GAP, in addition to having a worse performance than its older release, has a clear problem at the pattern recognition step under both null and high normal cell contamination levels, where the recognition fails in most of the samples. This is seen in its graphical output and reflected on the overall recall performance.

If we regard correct calls as those that match not only copy number but also LOH status, methods manage to keep around 90% of the correct calls, with the greatest recall drop being 13.2% for ASCAT under null contamination (see Additional file 5). Nevertheless, the recalling behaviour is similar to the case where only copy number is required to consider calls correct. Therefore, LOH status recalling is similar to the recalling ability seen in the overall performance.

Complementary to the recall rates, we investigated FDRs and how wrong calls are distributed among copy numbers (see Additional file 6). GPHMM and OncoSNP tend to call higher copy numbers than the real ones, whereas MixHMM and GenoCNA clearly do the opposite. Furthermore, as contamination increases, the calls of these latter two have a bias towards copy number 3, as also seen in Figure 3B. The reduction in their FDRs at higher contamination levels does not mean better specificity. Instead, it reflects the fact that these methods decrease the number of alteration calls, probably due to the high uncertainty under heavy contamination levels.

**Figure 4:**
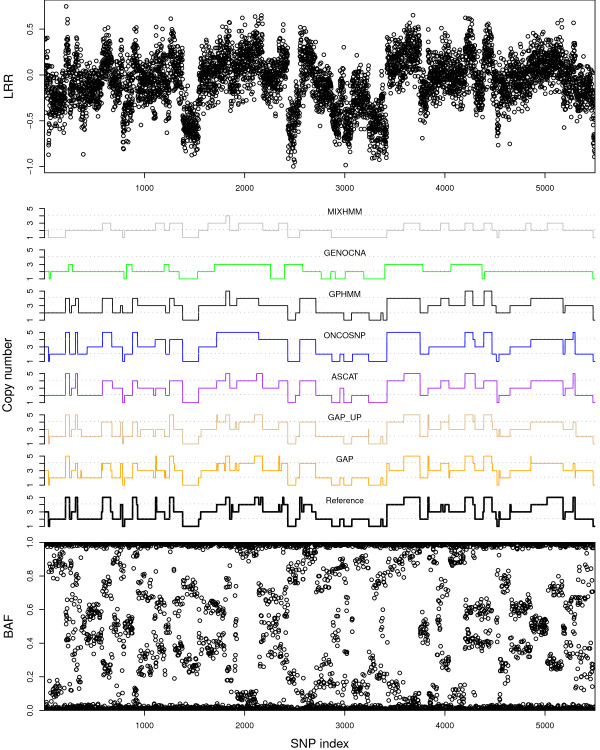
**Example synthetic genomic data and method calls.** LRR (top graph) and BAF (bottom graph) signals for a 5,500 SNP-long sequence of one of the complex-patterned samples with 25% normal contamination. In the middle, the calls made by the seven methods and the reference true calls. If any, calls made with copy numbers higher than 5 are displayed as copy number 5.

When we visualize each method´s calls on one of the complex-patterned samples at 25% contamination (Figure 4), we see that MixHMM and GenoCNA have a bias towards specific copy number, here copy number 2; that GPHMM, OncoSNP and ASCAT have similar call sequences, although each of them ascertains some specific regions; and that GAP is slightly better than the rest, specially because it is able to detect more short regions.

### Performance on hybrid samples

In order to assess whether (i) the spatial distribution of real genomic rearrangements and (ii) the inclusion of long regions result in different method performances from what is observed on purely synthetic data, we generated hybrid samples based on scaffolds taken from GAP's output [8] over three real tumour cell-line samples: BLC_B1_T17 (near-diploid), L_B1_T24B (high complexity) and MDA_468 (LOH-rich). Similarly to their fully synthetic counterparts, the samples were generated at 0, 25, 50 and 75% normal cell contamination levels (more details in Additional file 7).

In comparison to the tests on synthetic data, we observed slightly better performance in GPHMM and worse in OncoSNP and ASCAT (see Additional file 8). Nevertheless, GAP remained as the best performer, and MixHMM and GenoCNA as the worst. Furthermore, observations made using purely synthetic data on the effect of alteration pattern, LRR baseline shift and normal cell contamination also apply.

### Performance on cell-line samples

Given its superior and stable performance over synthetic data, the GAP output on the pure tumour sample was selected as the gold standard for the performance assessment over the cell-line data. There are additional reasons for the selection of GAP: (i) its ability to generate a visual output, which aids in the task of manual adjustment, also available, (ii) the fact that it is open source, which enables manual fine-tuning, and (iii) its confidence scores in the calls. After visual assessment of the output, calls were filtered to leave only those with maximum confidence in a scale from 1 to 4 and a minimum length of 40 SNPs, the length at which GAP recall rates under null contamination start to stabilize on synthetic data (Additional file 4). A total of 261 regions out of the original 367 were left. Only 12 of them were copy number 1, so results regarding this copy number were not expected to be reliable. Grouping recall rates by copy number and contamination level, we observed that, in general, methods behave on cell-line data similarly to synthetic data. Specifically, the samples are near-diploid with many copy number-neutral LOH regions and copy number 3 and 4 alterations, so we expected similar results to those with the synthetic LOH-enriched and near-triploid samples.

**Figure 5:**
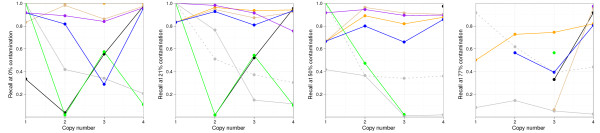
**Recall rates of CNAs on cell-line samples.** Recall rates (y-axis), by normal cell contaminations and copy number (x-axis), with respect to a high-confidence subset of CNAs detected by GAP on the cell-line pure tumour sample. Colour code: GAP (orange), updated GAP (golden), ASCAT (purple) GPHMM (black), OncoSNP (blue), MixHMM (grey), MixHMM with manual parameterization (dashed grey) and GenoCNA (green).

We examined the recall rates at the available contamination levels closer to those in synthetic data: 0%, 21%, 50% and 77% (see Figure 5). Except at 77% contamination, ASCAT proves to be the best performer together with GAP and the updated GAP, and its recall rates are stable throughout the different copy numbers. The updated GAP recognizes the pattern under null contamination, although as we saw on synthetic data, this does not always happen. Additionally, both ASCAT and the updated GAP fail to recognize the alteration pattern at 77% contamination, an issue also observed with synthetic data, and the old GAP is the only method that still correctly estimates the LRR shift at such contamination, a fact that is independent from having selected GAP as a reference. Despite a general good performance, OncoSNP has trouble with copy number 3 regions under null contamination, as it had with the synthetic near-triploid samples. Cell-line results also confirm that MixHMM and GenoCNA are unable to correctly recall most high copy number regions and have a tendency to call higher copy numbers as contamination increases. Manually providing the contamination and LRR shift parameters improves MixHMM recall rates, but its high sensitivity to changes in intensity result in overfragmented calls (see Additional file 9). Finally, whereas GPHMM manages to carry out a rather correct breakpoint detection, it fails to estimate the LRR baseline shift on all four contamination levels. Hence, all copy numbers are increased and recall rates are minimum except for copy numbers 4 and higher, given that they are grouped. GPHMM’s results stress the importance of correct LRR baseline shift estimation, whether it is made directly, or through ploidy or DNA index estimation. We wish to note that the baseline shift can be correctly estimated (see GPHMM paper [13]), if a PFB [19] with a modified specification is used. 

The greater approximation of ASCAT and updated GAP calls to the GAP reference in comparison to GAP calls on non-pure samples validates the gold standard approach we took, based on the results from the best method over the pure tumour sample.

### Parameter relationships

In [8], the reduction of signal that is lost proportionally to the level of normal cell contamination is modelled as a separate parameter q, the coefficient of contraction. However, such parameter only makes sense under experimental variability, given its actual theoretical linear relationship with normal contamination. As expected, a linear regression of these two parameters in the samples analyzed by Popova et al. [8] shows a high correlation (R²=0.8, R²=0.9 without one outlier) (see Additional file 10), where the coefficient of LRR contraction is approximately half of the level of tumour purity (q=0.49(1-p)). The definition of our synthetic data automatically generates this correlation.

Given that DNA index is linearly affected by normal cell contamination, the relationship between baseline shift and contamination described by Li et al. [13] can be redefined in terms of DNA index against baseline shift. Using the breast cancer samples profiled by Popova et al. [8], we compared the DNA index and the baseline shift obtained by GPHMM [13] and found a high correlation (R²=0.89) (see Additional file 10). The baseline shift for our synthetic samples was thus established based on this correlation.

Depending on the estimation of the baseline shift, different and plausible biological solutions may be obtained for a sample, and it has been stated that alternative experiments, such as fluorescence in situ hybridization (FISH), are required in such cases [12-14]. We reckon that a sensible integration of the aforementioned relationships into future methods' models can aid on the restriction of possible solutions. Still, because of the convolution of tumour subclones, in presence of normal cell contamination, regions with 2 or more tumour subclones cannot be uniquely genotyped [11] with current SNP array technology.

## Conclusions

The model implemented in the software CnaGen, which integrates normal cell contamination and intra-tumour heterogeneity, has proven to generate synthetic samples that mimic the characteristic BAF and LRR signal patterns of real tumour samples. Supporting this, synthetic, hybrid and cell-line data reach similar outcomes in the assessment of the performance of the methods tested in this work.

When it comes to selecting a method to detect germline copy number variations (CNVs), where the additional tumour-related issues do not apply, some authors consider that it is better to use several algorithms and compare data [20], while others consider that this approach might not be appropriate [21]. Given the results of the present study, we consider that the safest option would be to carefully choose a good method that is adequate for the characteristics of the data and knowledge of the researcher. For example, in our case, GAP is the best method, outperforming the rest in nearly all situations, followed by ASCAT and GPHMM, even though this latter and OncoSNP present a more sustained performance at heavy contamination levels. Yet, because the success of a method does not solely depend on performance, but also on the interaction between the user and the software implementation, GAP and ASCAT may not be suitable for users without basic programming skills, who may prefer the easy to use GPHMM. Thus, bearing in mind performance, parameterization and ease of use, we recommend GAP for advanced users and GPHMM for a fully driven analysis.

## Competing interests

The authors declare no competing interests.

## Authors' contributions

DMA, AMA and NRE conceived the study. DMA and NRE wrote the manuscript. DMA devised the statistical model, generated the synthetic samples, ran the analyses and compared the methods. NRE participated in the specification of the synthetic samples and in the comparison of the methods. All authors read and approved the final manuscript.

## Supplementary Material

Additional file 1 Code to generate the synthetic samples. Click here for file

Additional file 2 ** Synthetic regions without noise.**Synthetic BAF (left graph) and LRR (right graph) signals of some example regions generated with CnaGen at different contamination levels and without probe-specific and autocorrelated noises: a heterozygous deletion (first column), a normal diploid region (second column), the various heterozygous CNA events up to copy number 5 (third to seventh columns) and two concrete cases of 2 and 3-subclone CNAs (last two columns). Each SNP probe provides a measurement of the proportion of one of the alleles (BAF) and the total intensity coming from the two alleles (LRR). Click here for file

Additional file 3 ** Recall rates by method, contamination and alteration length.** Recall rates (y-axis) of each of the assessed methods, calculated by contamination and alteration length over each of the 5 synthetic sample sets. Colour code: GAP (orange); Colour code: GAP (orange), updated GAP (golden), ASCAT (purple), GPHMM (black), OncoSNP (blue), GenoCNA (green), MixHMM (grey). Click here for file

Additional file 4 ** Recall rates, considering LOH status, by method, contamination, and alteration copy number and length.** Recall rates (y-axis) of calls made with correct copy number and LOH status. By: (i) normal cell contamination (x-axis), (ii) contamination and copy number (x-axis), and (iii) contamination and alteration length (x-axis) over each of the 5 synthetic sample sets. Colour code: GAP (orange), updated GAP (golden), ASCAT (purple), GPHMM (black), OncoSNP (blue), GenoCNA (green), MixHMM (grey). Click here for file

Additional file 5 ** Method version and parameterization details.** Versions of the methods used in this study and details of parameterization when the default parameters were not used. Additionally, details on the PFB and GC content files used as input when required. Click here for file

Additional file 6 ** FDRs on synthetic samples.** Overall false discovery rates on synthetic samples, broken down by normal cell contamination level and called/real copy number. Cell colour represents the amount of incorrectly made calls when the predicted copy number (y-axis) is different from the actual copy number (x-axis). There are no copy number 0 regions in the samples, but some methods make copy number 0 calls. The total FDR for a certain method and contamination is indicated in the lower right corner of each plot, and is the sum of all the corresponding cell values. Good performance is reflected in the symmetry and narrowness of the wrong call distribution along the correct call diagonal. Departure from such symmetry evidences some kind of bias. Click here for file

Additional file 7 Generation of hybrid samples. Click here for file

Additional file 8 ** Recall rates by method, contamination, and alteration length.** (A) Recall rates (y-axis) of each of the assessed methods, calculated by contamination over each of the 3 hybrid sample sets. Colour code: GAP (orange), updated GAP (golden), ASCAT (purple), GPHMM (black), OncoSNP (blue), GenoCNA (green), MixHMM (grey). (B) Recall rates (y-axis) of each of the assessed methods, calculated by contamination and alteration length over each of the 3 hybrid sample sets. Alteration lengths (y-axis) are grouped into increasingly bigger bins (10–19 SNPs, 20–39, 40–79, 80–159, 160–319, 320–639, 640–1279 and from 1280 SNPs on) and each bin is represented by the shorter length within it. Alterations shorter than 10 SNPs were not assessed. Colour code: GAP (orange), updated GAP (golden), ASCAT (purple), GPHMM (black), OncoSNP (blue), GenoCNA (green), MixHMM (grey). Click here for file

Additional file 9 ** Cell-line data and method calls.** LRR (top graph) and BAF (bottom graph) signals for the cell-line sample at 21% contamination. Chromosomes 6, 16 and X are excluded for the reasons described in the main text. In the middle, the calls made by the seven methods, including MixHMM with manually set global parameters (LRR shift and contamination), and the reference true calls. If any, calls made with copy numbers higher than 4 are displayed as copy number 4. Click here for file

Additional file 10 ** Parameter relationships.** Tables and regression plots that show the relationship between: (i) coefficient of LRR contraction and degree of normal cell contamination; and (ii) DNA index and baseline shift. Click here for file
