# Supplementary figures and images for: Comparison of methods to detect copy number alterations in cancer using simulated and real genotyping data
Source: BMC Bioinformatics. 2012 Aug 7;13:192. doi: 10.1186/1471-2105-13-192 (PMC3472297; doi:10.1186/1471-2105-13-192)

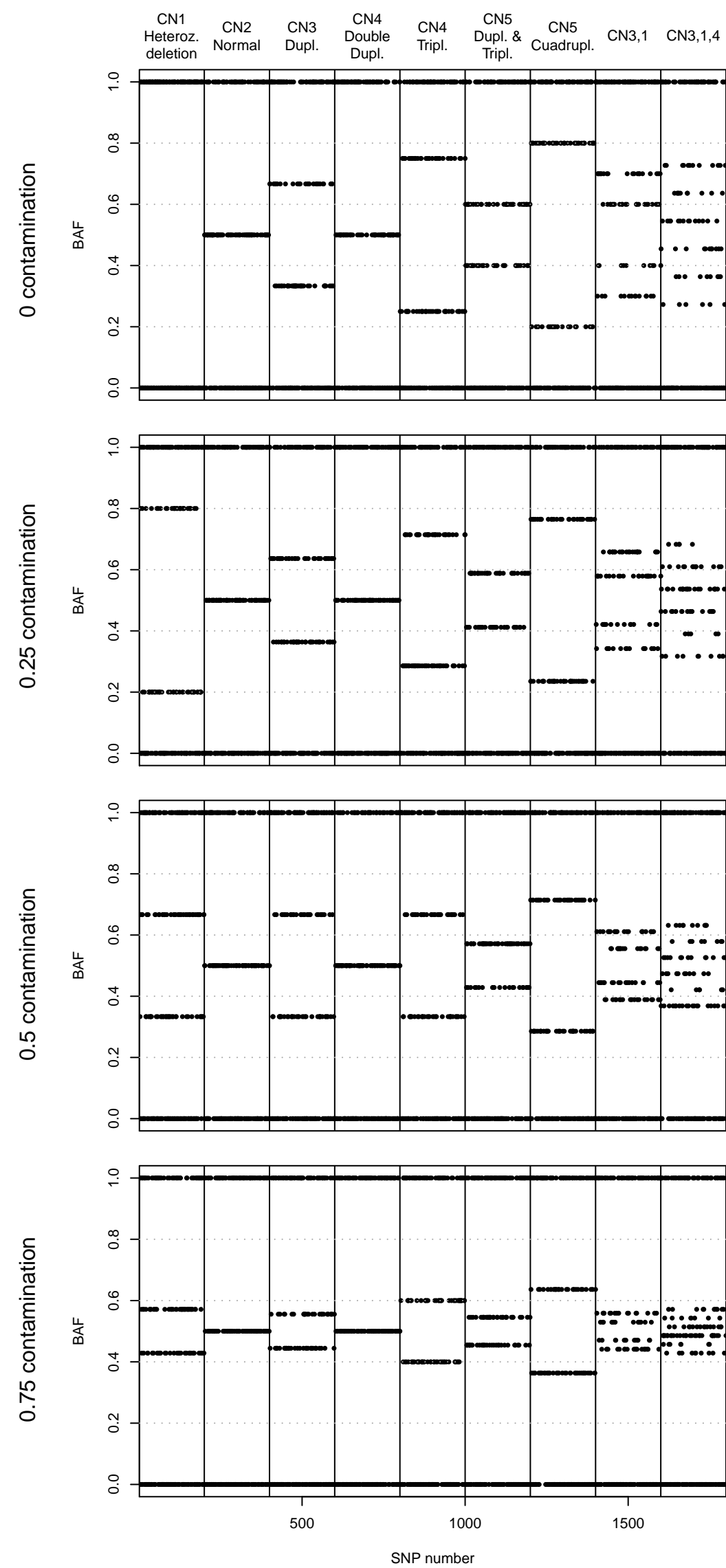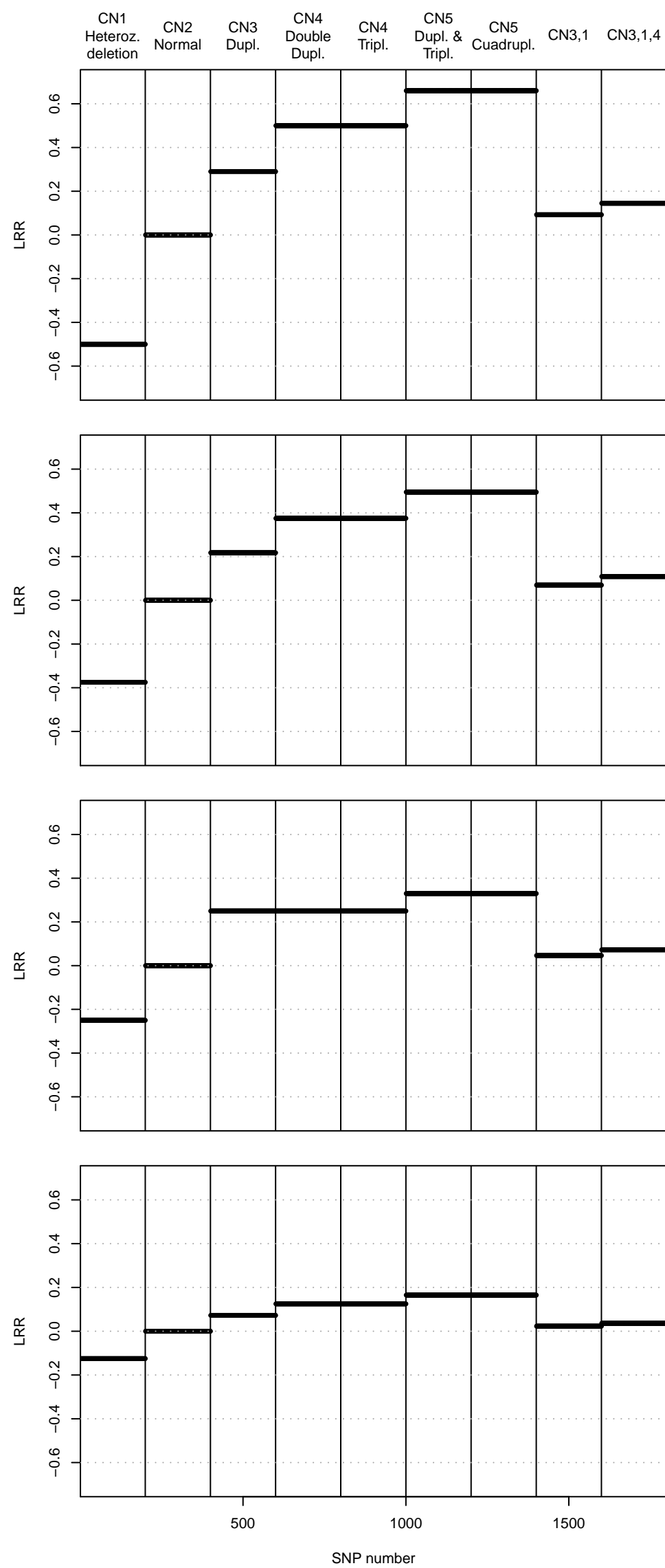

Supplement: Additional file 2 — Synthetic regions without noise.Synthetic BAF (left graph) and LRR (right graph) signals of some example regions generated with CnaGen at different contamination levels and without probe-specific and autocorrelated noises: a heterozygous deletion (first column), a normal diploid region (second column), the various heterozygous CNA events up to copy number 5 (third to seventh columns) and two concrete cases of 2 and 3-subclone CNAs (last two columns). Each SNP probe provides a measurement of the proportion of one of the alleles (BAF) and the total intensity coming from the two alleles (LRR). [file 1471-2105-13-192-S2.pdf]

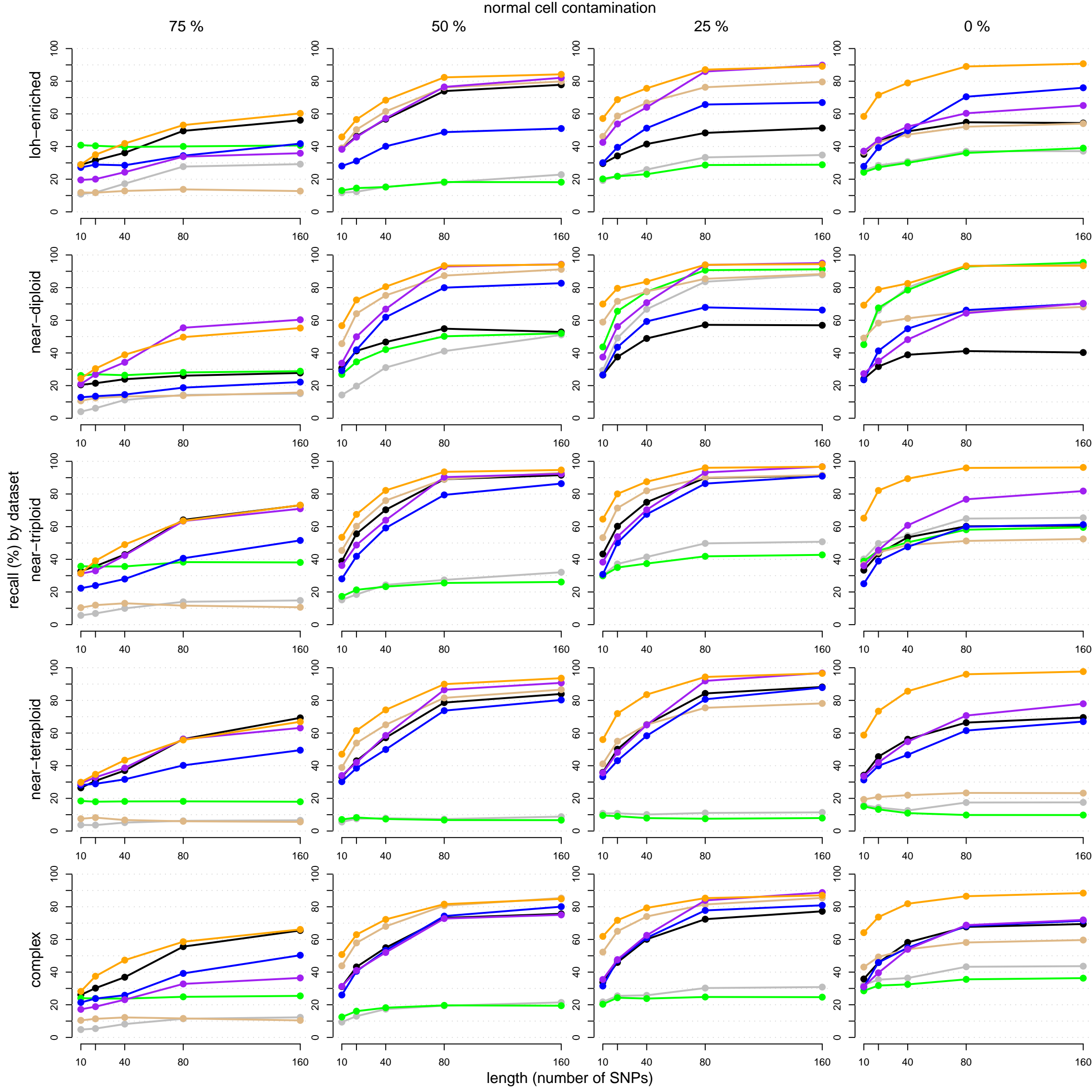

Supplement: Additional file 3 — Recall rates by method, contamination and alteration length. Recall rates (y-axis) of each of the assessed methods, calculated by contamination and alteration length over each of the 5 synthetic sample sets. Colour code: GAP (orange); Colour code: GAP (orange), updated GAP (golden), ASCAT (purple), GPHMM (black), OncoSNP (blue), GenoCNA (green), MixHMM (grey). [file 1471-2105-13-192-S3.pdf]

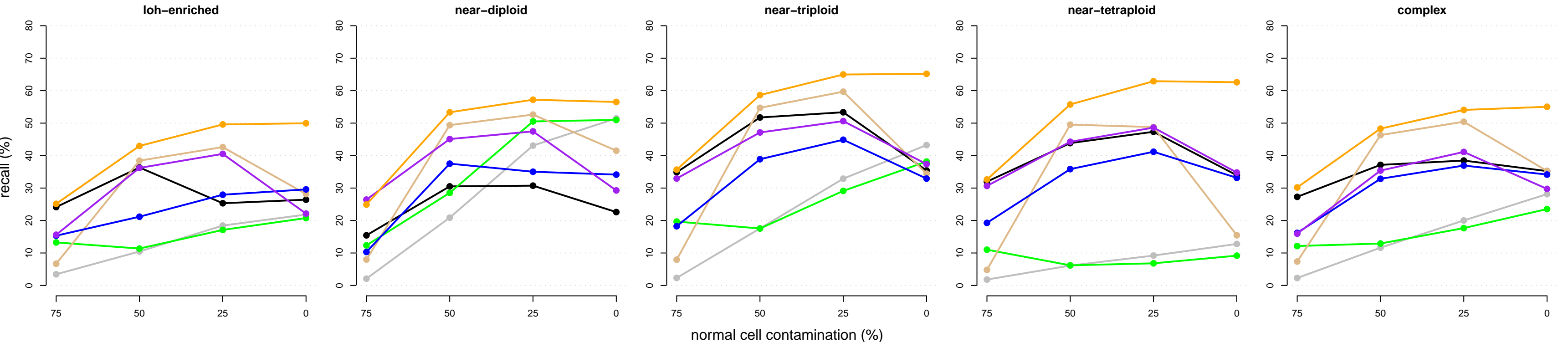

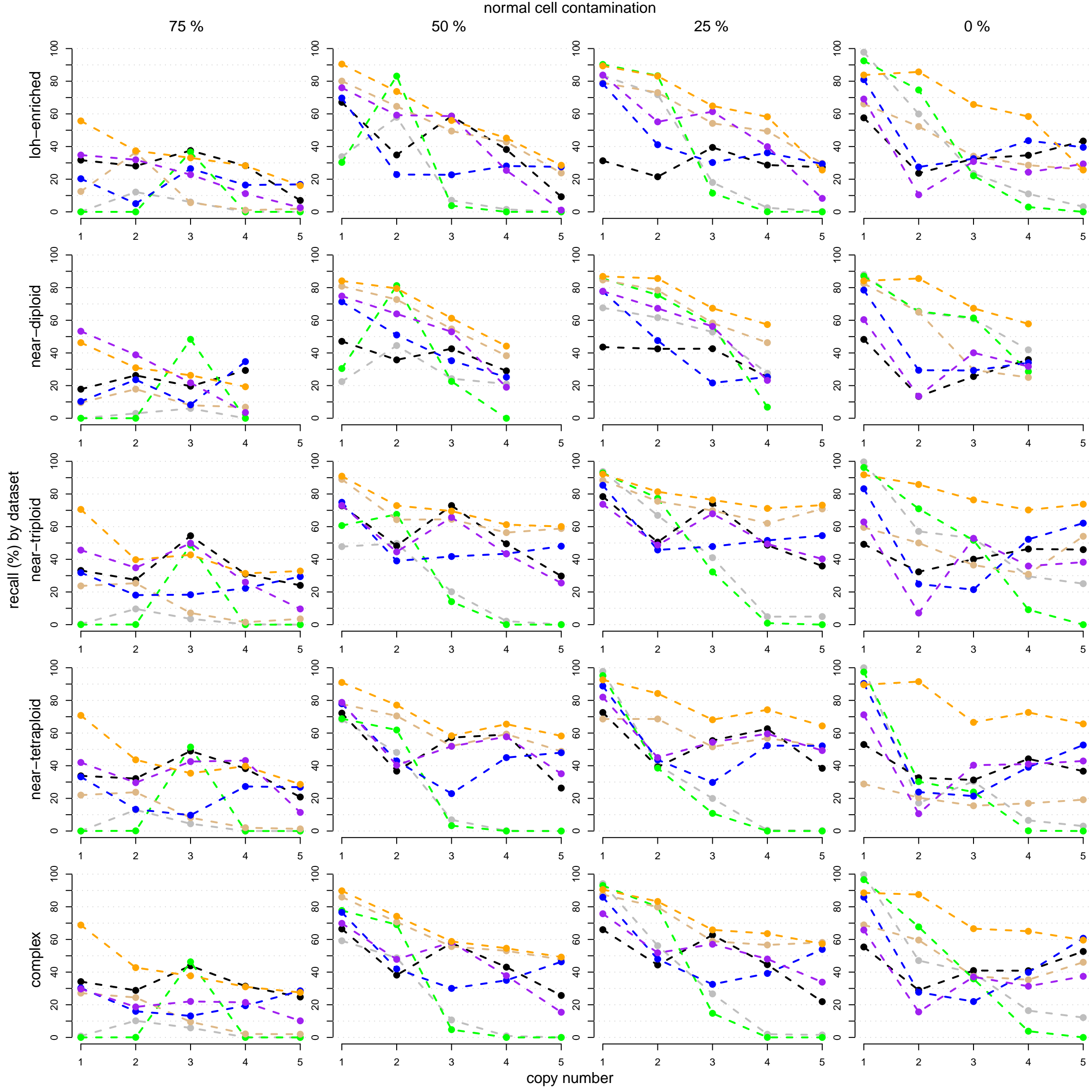

normal cell contamination

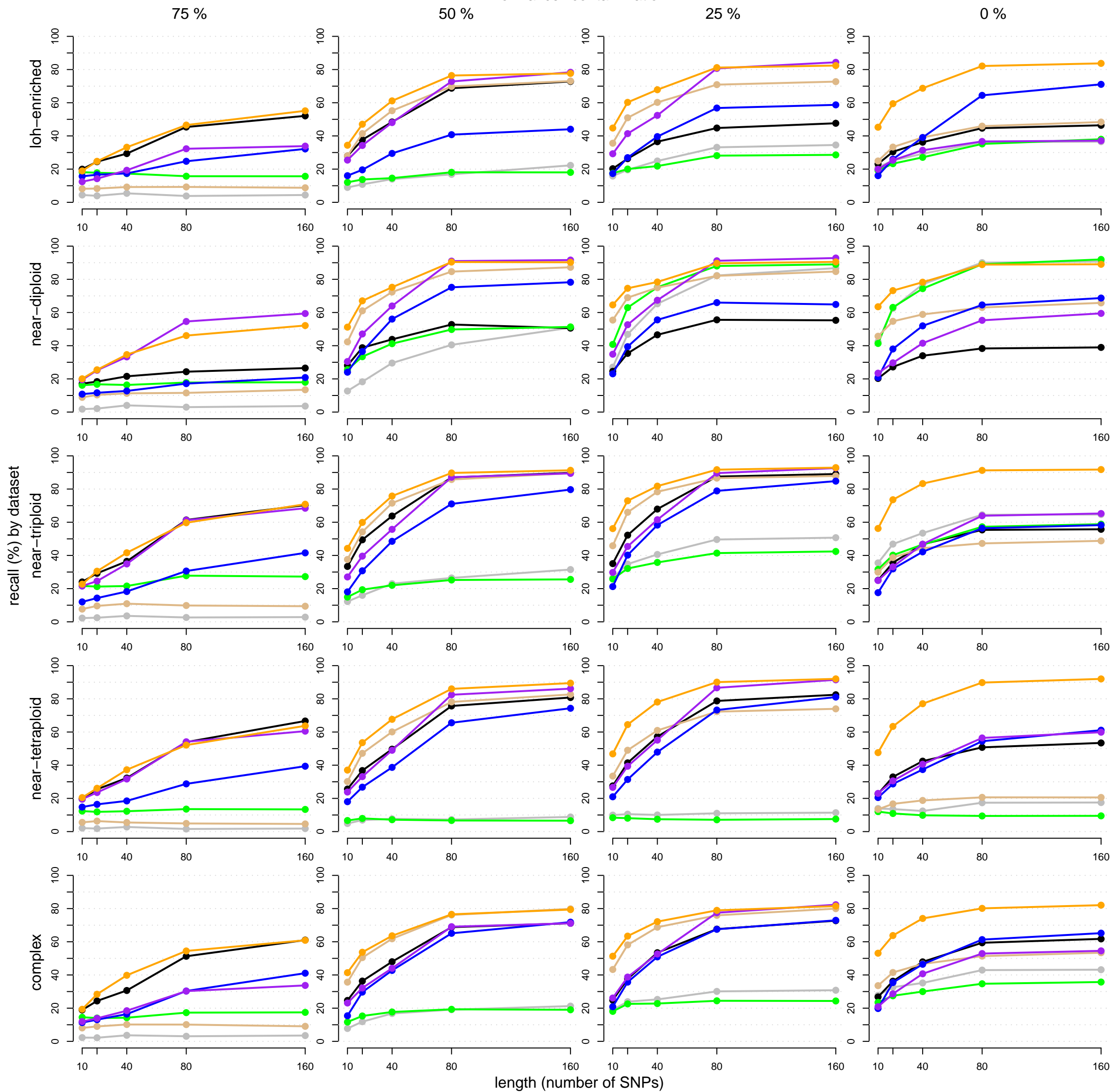

Supplement: Additional file 4 — Recall rates, considering LOH status, by method, contamination, and alteration copy number and length. Recall rates (y-axis) of calls made with correct copy number and LOH status. By: (i) normal cell contamination (x-axis), (ii) contamination and copy number (x-axis), and (iii) contamination and alteration length (x-axis) over each of the 5 synthetic sample sets. Colour code: GAP (orange), updated GAP (golden), ASCAT (purple), GPHMM (black), OncoSNP (blue), GenoCNA (green), MixHMM (grey). [file 1471-2105-13-192-S4.pdf]

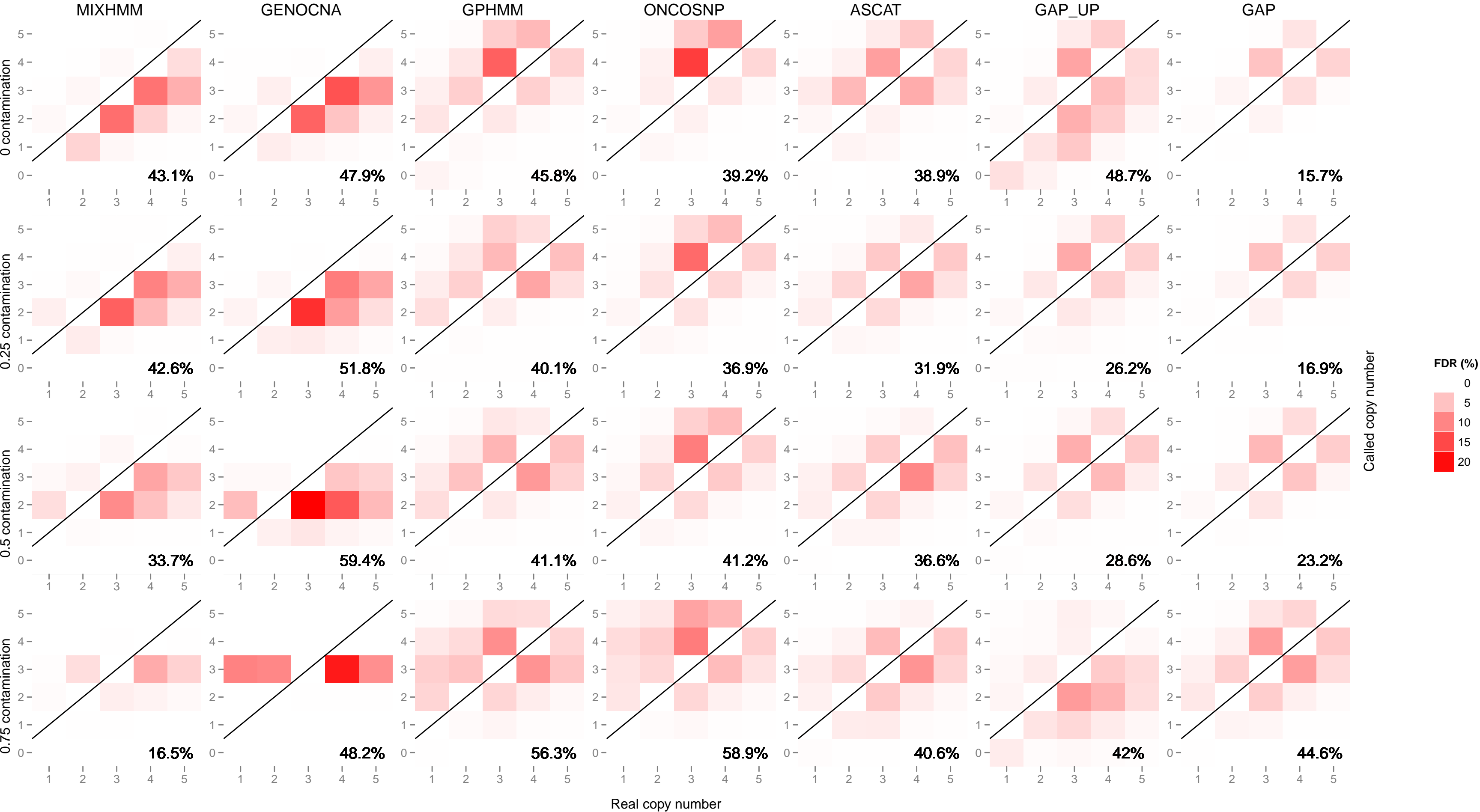

Supplement: Additional file 6 — FDRs on synthetic samples. Overall false discovery rates on synthetic samples, broken down by normal cell contamination level and called/real copy number. Cell colour represents the amount of incorrectly made calls when the predicted copy number (y-axis) is different from the actual copy number (x-axis). There are no copy number 0 regions in the samples, but some methods make copy number 0 calls. The total FDR for a certain method and contamination is indicated in the lower right corner of each plot, and is the sum of all the corresponding cell values. Good performance is reflected in the symmetry and narrowness of the wrong call distribution along the correct call diagonal. Departure from such symmetry evidences some kind of bias. [file 1471-2105-13-192-S6.pdf]

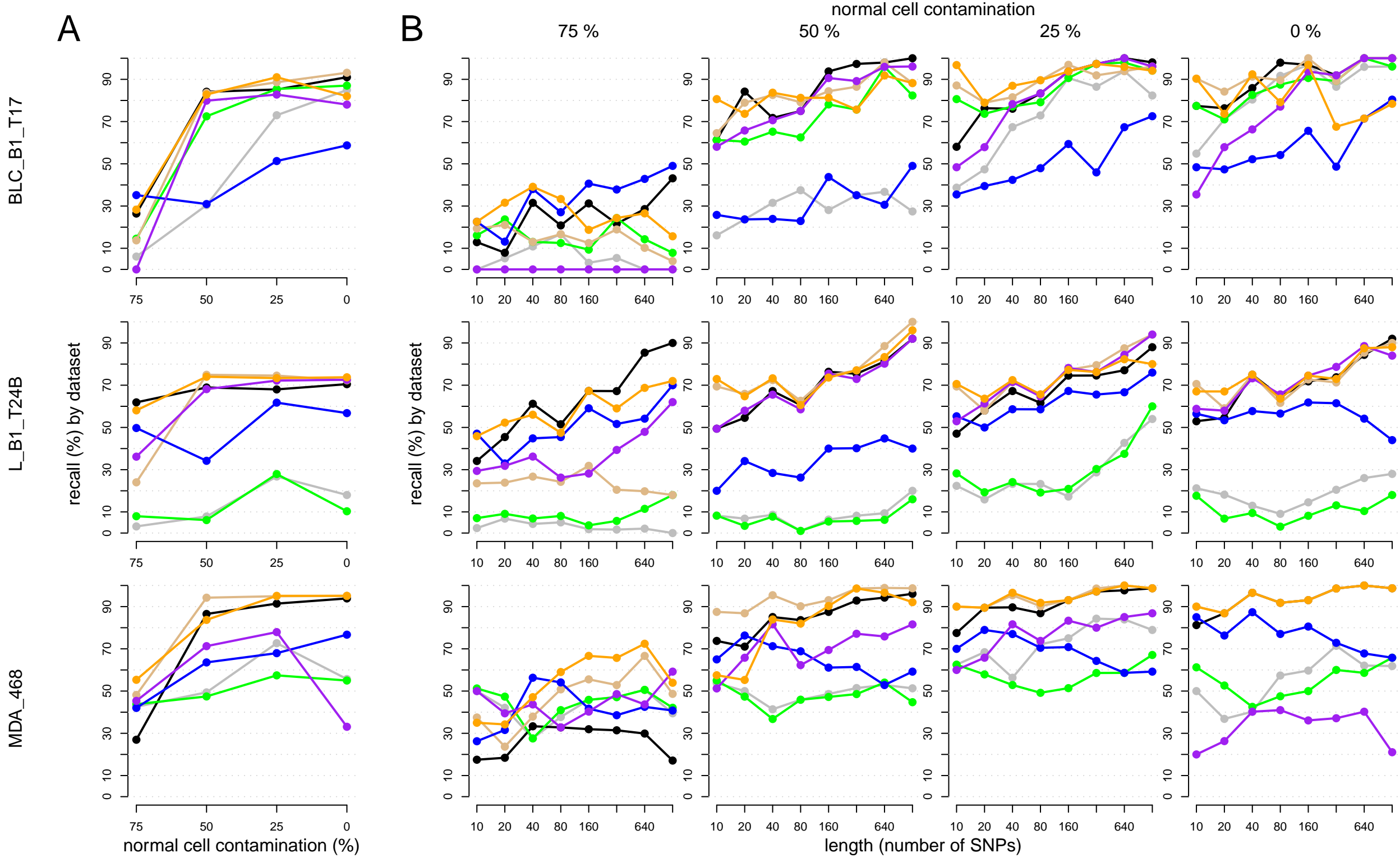

Supplement: Additional file 8 — Recall rates by method, contamination, and alteration length. (A) Recall rates (y-axis) of each of the assessed methods, calculated by contamination over each of the 3 hybrid sample sets. Colour code: GAP (orange), updated GAP (golden), ASCAT (purple), GPHMM (black), OncoSNP (blue), GenoCNA (green), MixHMM (grey). (B) Recall rates (y-axis) of each of the assessed methods, calculated by contamination and alteration length over each of the 3 hybrid sample sets. Alteration lengths (y-axis) are grouped into increasingly bigger bins (10–19 SNPs, 20–39, 40–79, 80–159, 160–319, 320–639, 640–1279 and from 1280 SNPs on) and each bin is represented by the shorter length within it. Alterations shorter than 10 SNPs were not assessed. Colour code: GAP (orange), updated GAP (golden), ASCAT (purple), GPHMM (black), OncoSNP (blue), GenoCNA (green), MixHMM (grey). [file 1471-2105-13-192-S8.pdf]

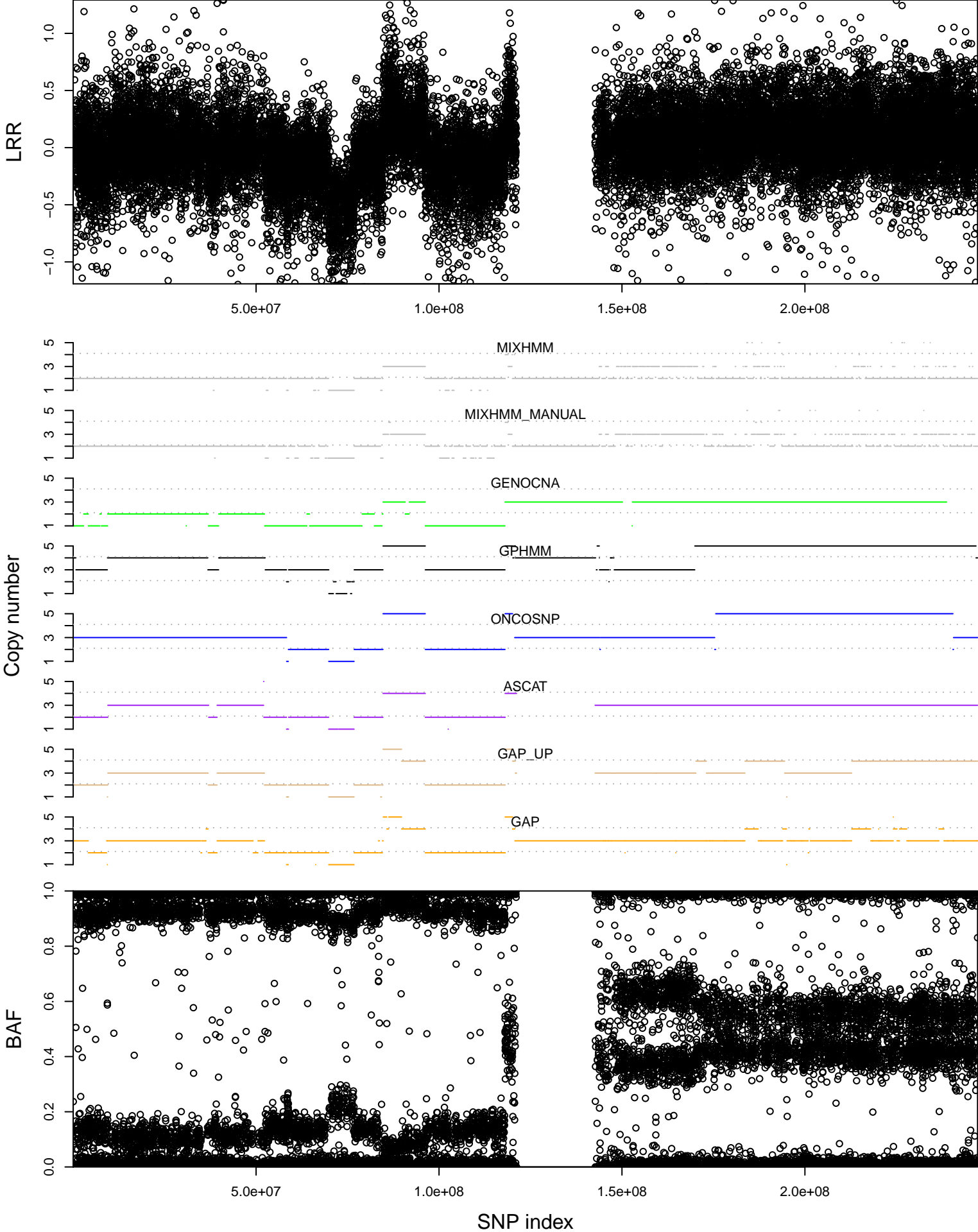

Supplement: Additional file 9 — Cell-line data and method calls. LRR (top graph) and BAF (bottom graph) signals for the cell-line sample at 21% contamination. Chromosomes 6, 16 and X are excluded for the reasons described in the main text. In the middle, the calls made by the seven methods, including MixHMM with manually set global parameters (LRR shift and contamination), and the reference true calls. If any, calls made with copy numbers higher than 4 are displayed as copy number 4. [file 1471-2105-13-192-S9.zip › chr1_calls.pdf]

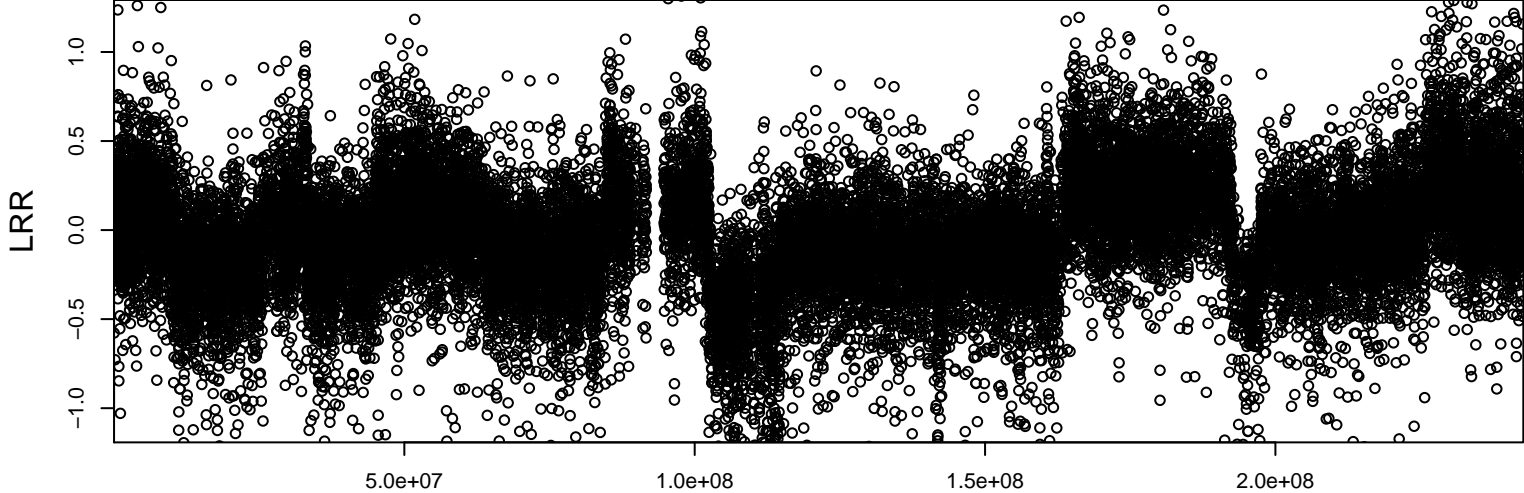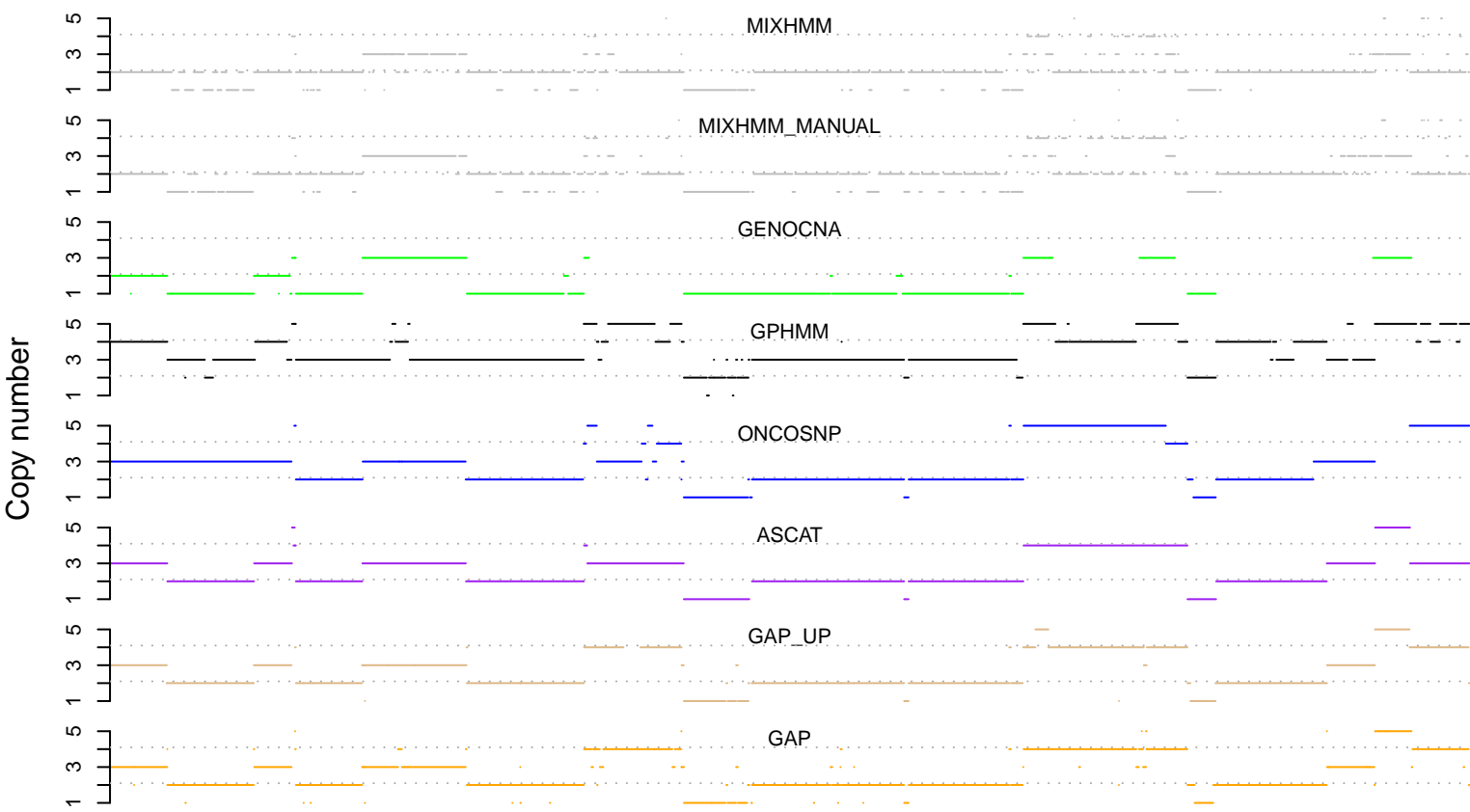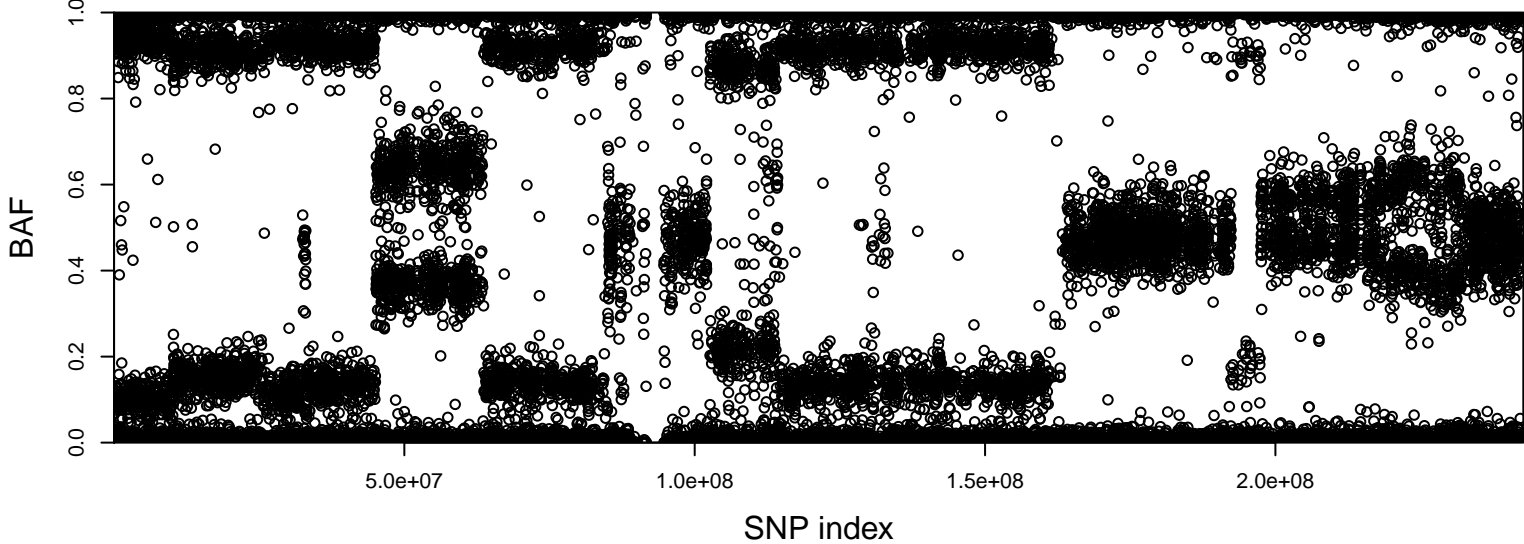

Supplement: Additional file 9 — Cell-line data and method calls. LRR (top graph) and BAF (bottom graph) signals for the cell-line sample at 21% contamination. Chromosomes 6, 16 and X are excluded for the reasons described in the main text. In the middle, the calls made by the seven methods, including MixHMM with manually set global parameters (LRR shift and contamination), and the reference true calls. If any, calls made with copy numbers higher than 4 are displayed as copy number 4. [file 1471-2105-13-192-S9.zip › chr2_calls.pdf]

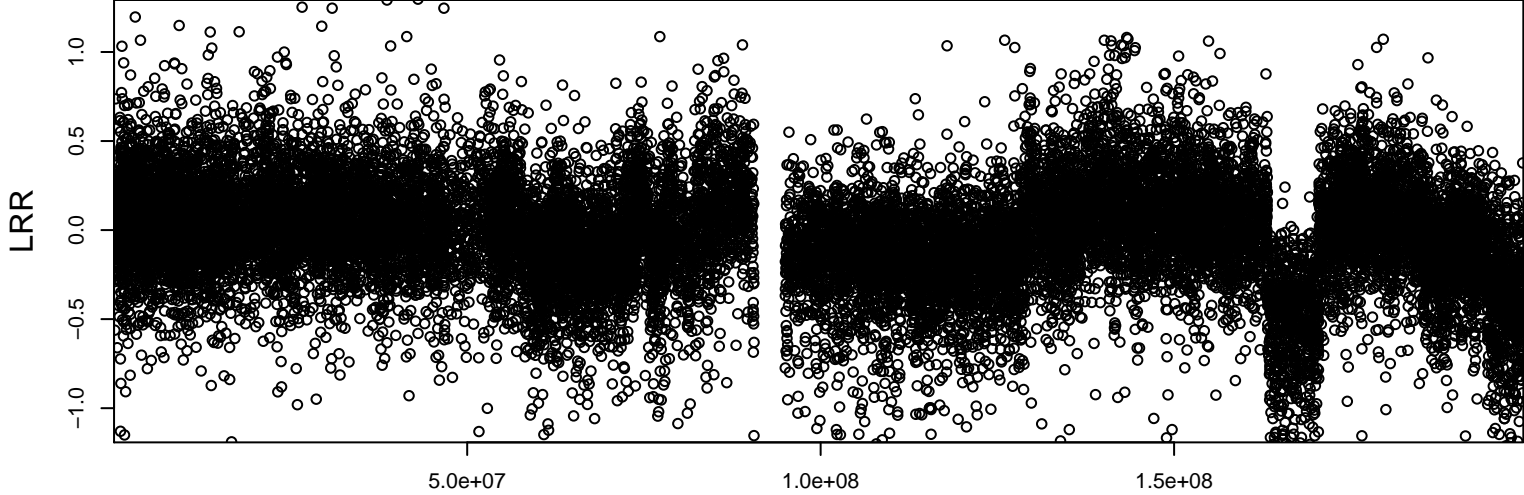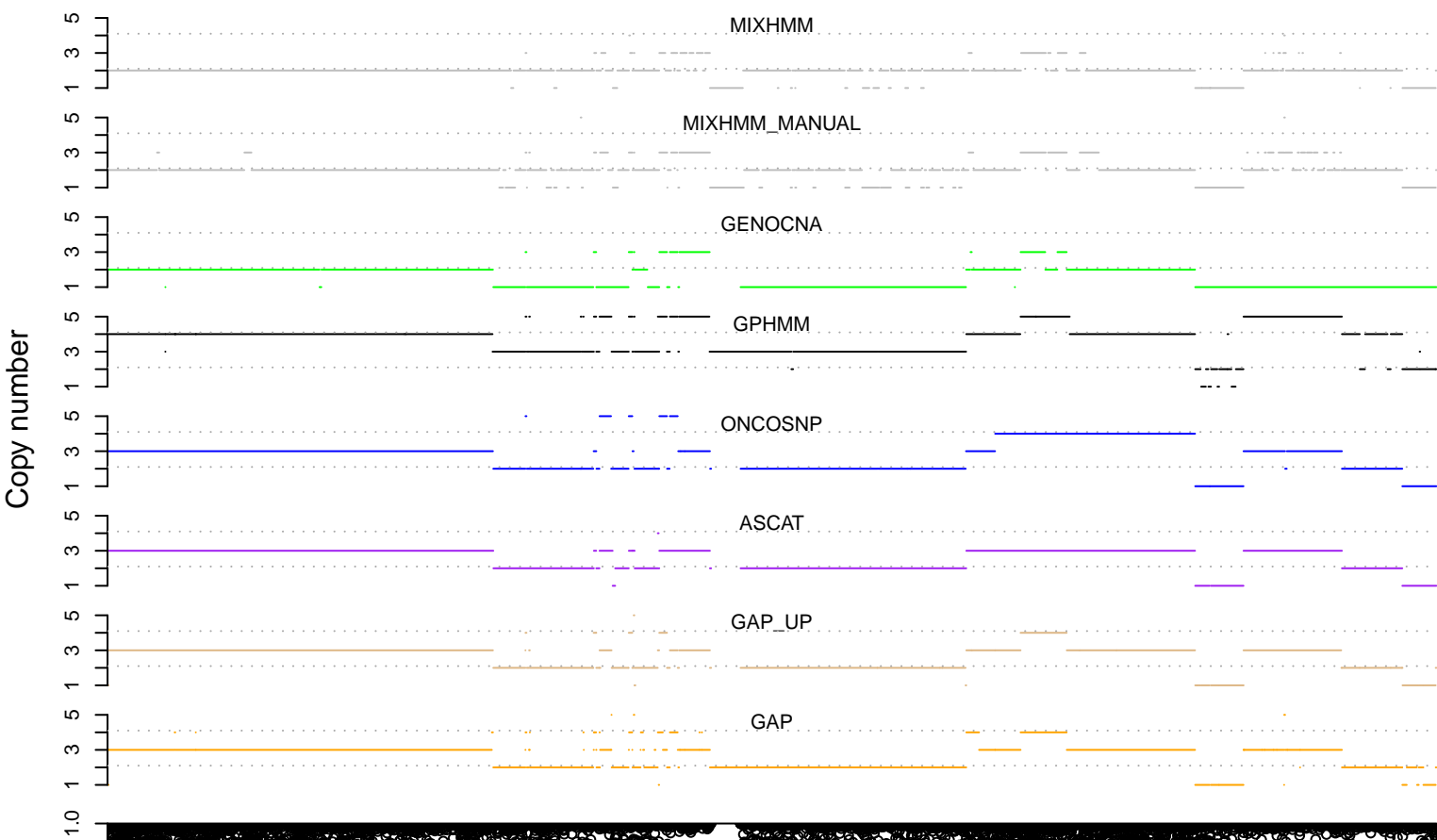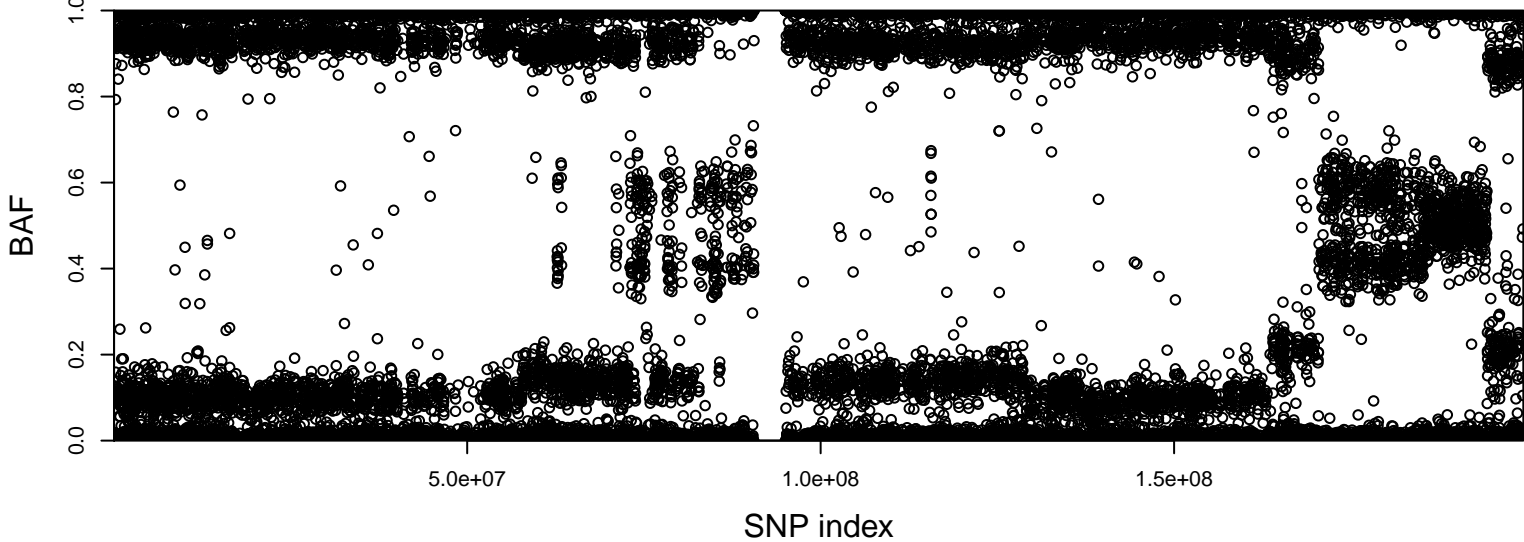

Supplement: Additional file 9 — Cell-line data and method calls. LRR (top graph) and BAF (bottom graph) signals for the cell-line sample at 21% contamination. Chromosomes 6, 16 and X are excluded for the reasons described in the main text. In the middle, the calls made by the seven methods, including MixHMM with manually set global parameters (LRR shift and contamination), and the reference true calls. If any, calls made with copy numbers higher than 4 are displayed as copy number 4. [file 1471-2105-13-192-S9.zip › chr3_calls.pdf]

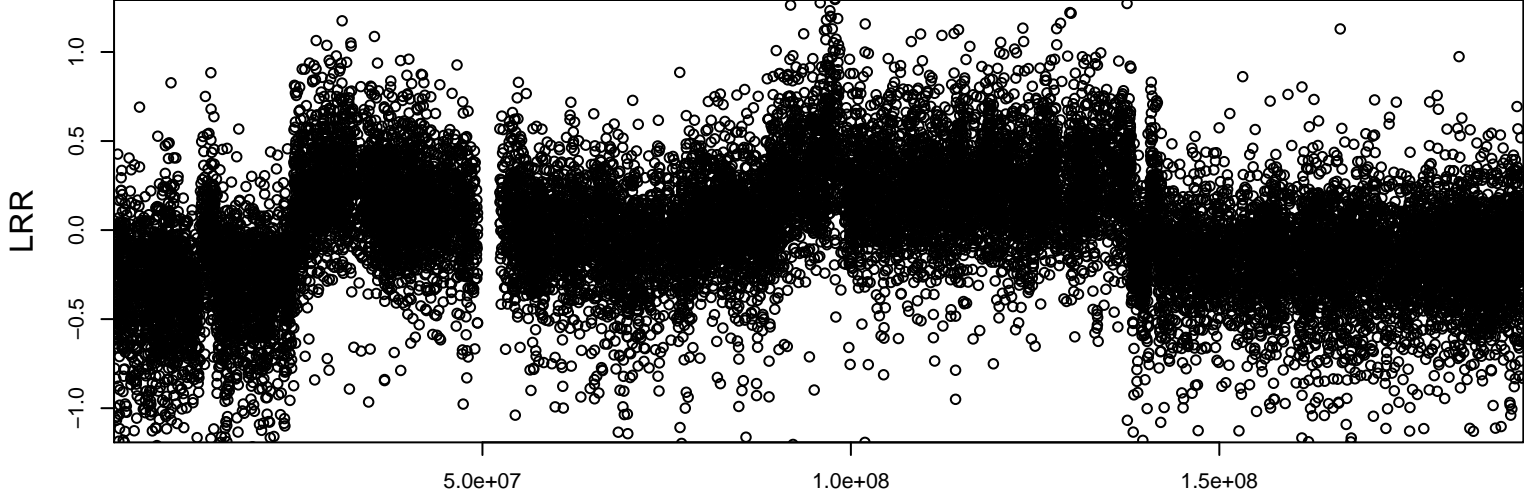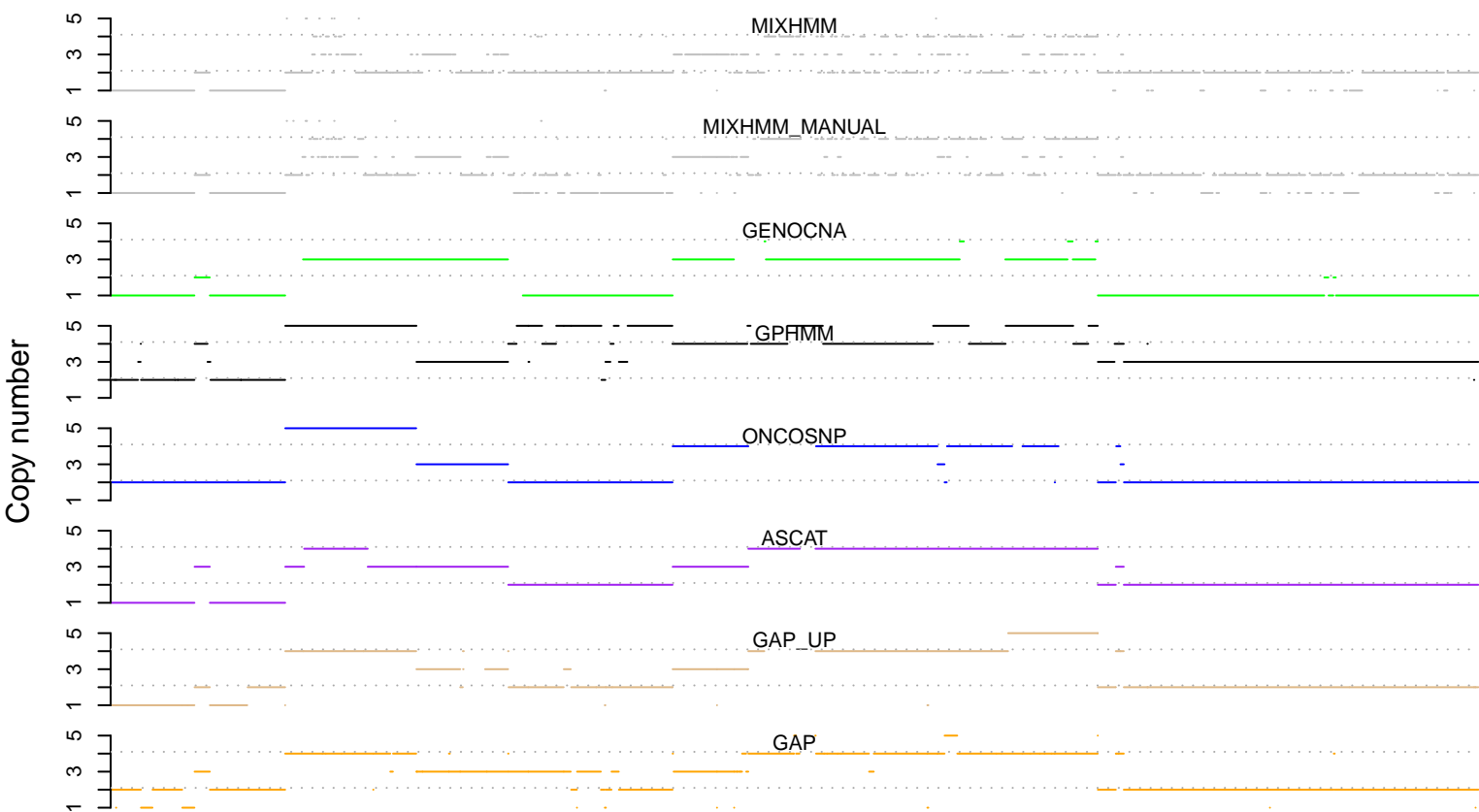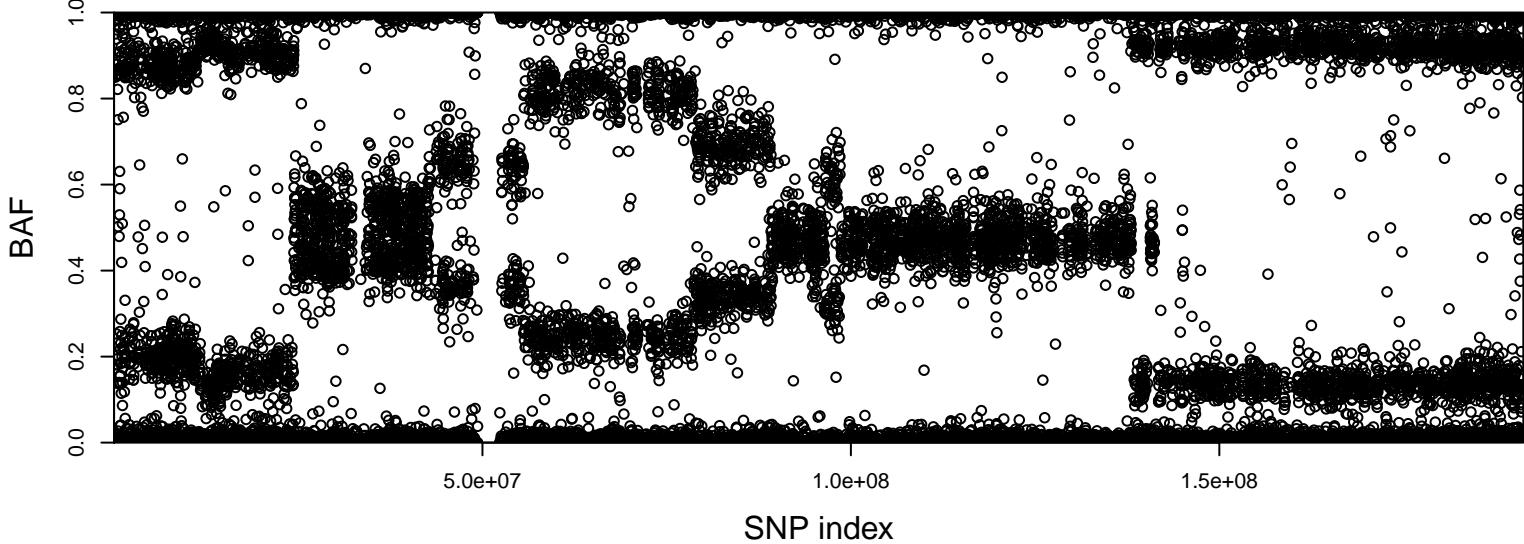

Supplement: Additional file 9 — Cell-line data and method calls. LRR (top graph) and BAF (bottom graph) signals for the cell-line sample at 21% contamination. Chromosomes 6, 16 and X are excluded for the reasons described in the main text. In the middle, the calls made by the seven methods, including MixHMM with manually set global parameters (LRR shift and contamination), and the reference true calls. If any, calls made with copy numbers higher than 4 are displayed as copy number 4. [file 1471-2105-13-192-S9.zip › chr4_calls.pdf]

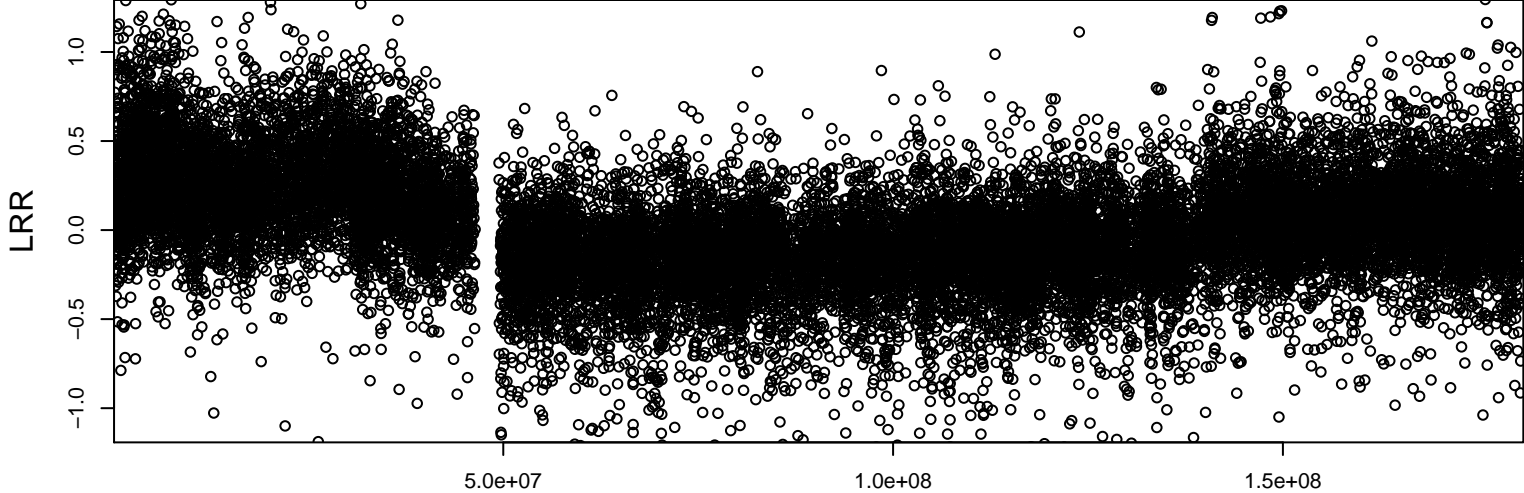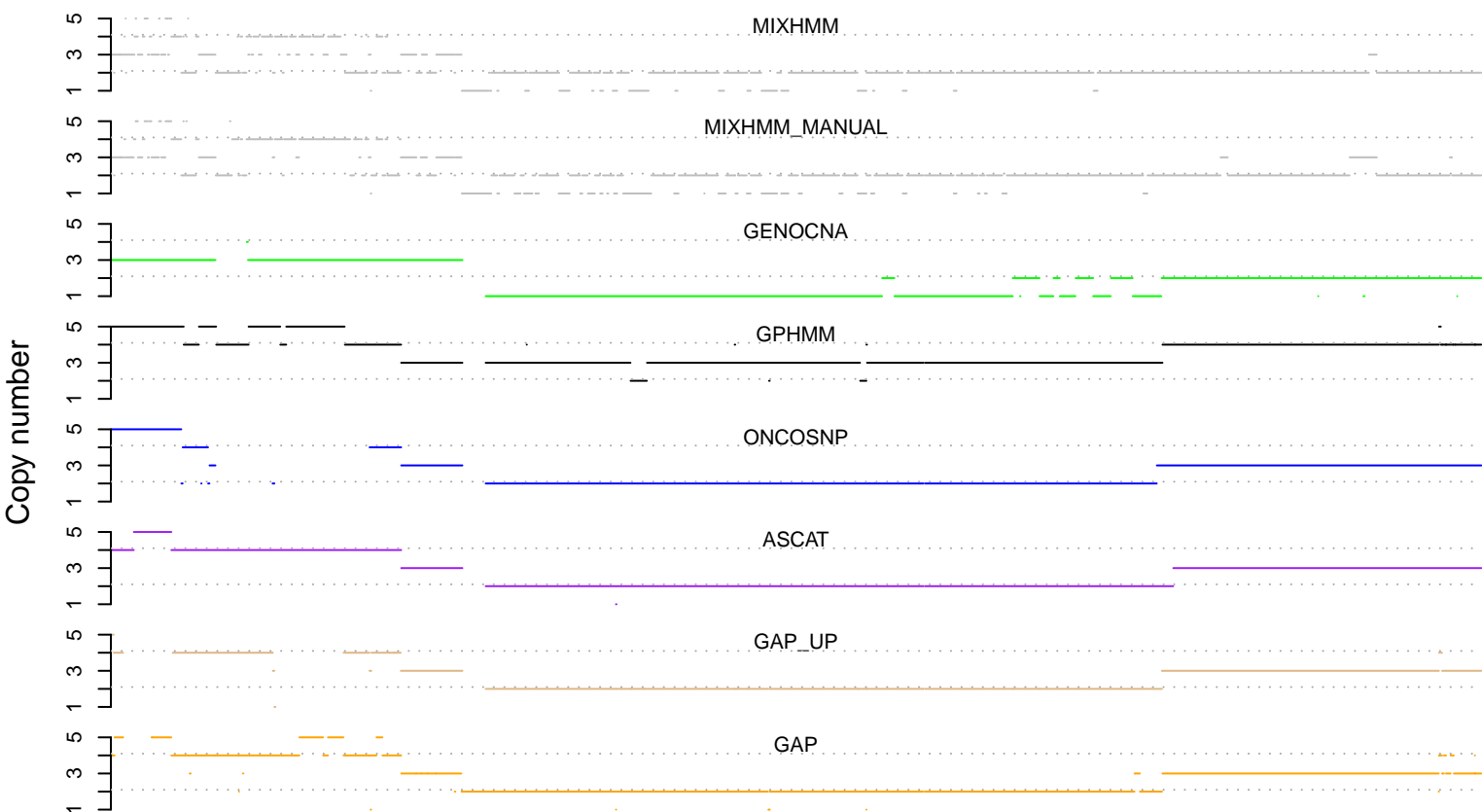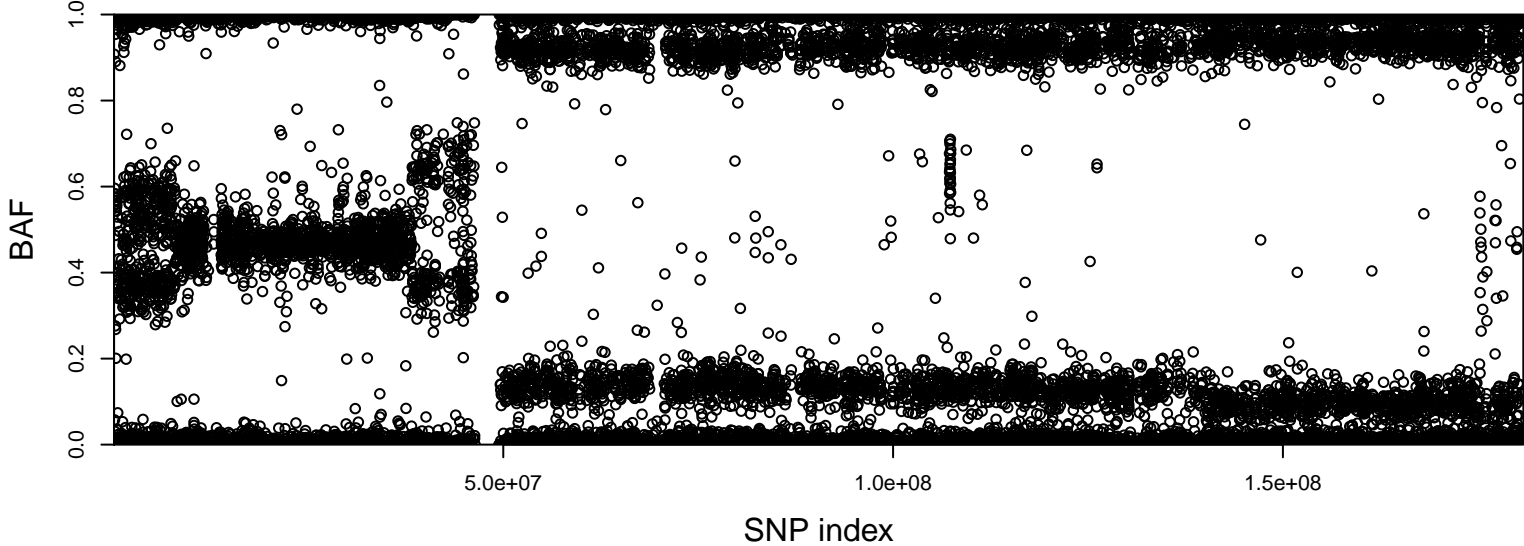

Supplement: Additional file 9 — Cell-line data and method calls. LRR (top graph) and BAF (bottom graph) signals for the cell-line sample at 21% contamination. Chromosomes 6, 16 and X are excluded for the reasons described in the main text. In the middle, the calls made by the seven methods, including MixHMM with manually set global parameters (LRR shift and contamination), and the reference true calls. If any, calls made with copy numbers higher than 4 are displayed as copy number 4. [file 1471-2105-13-192-S9.zip › chr5_calls.pdf]

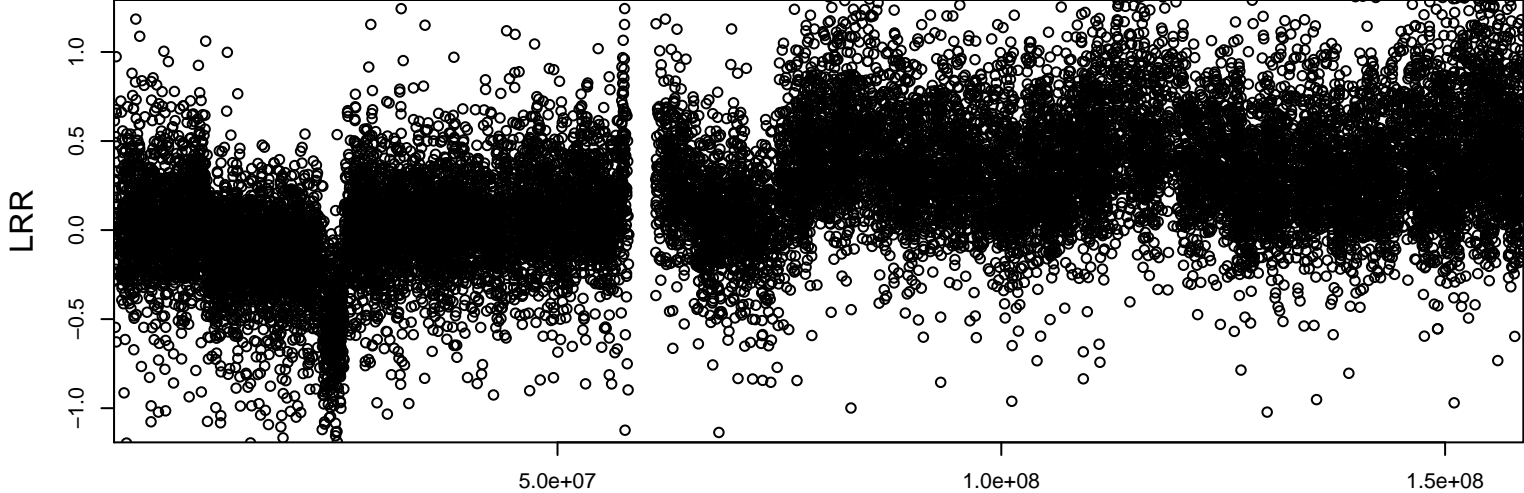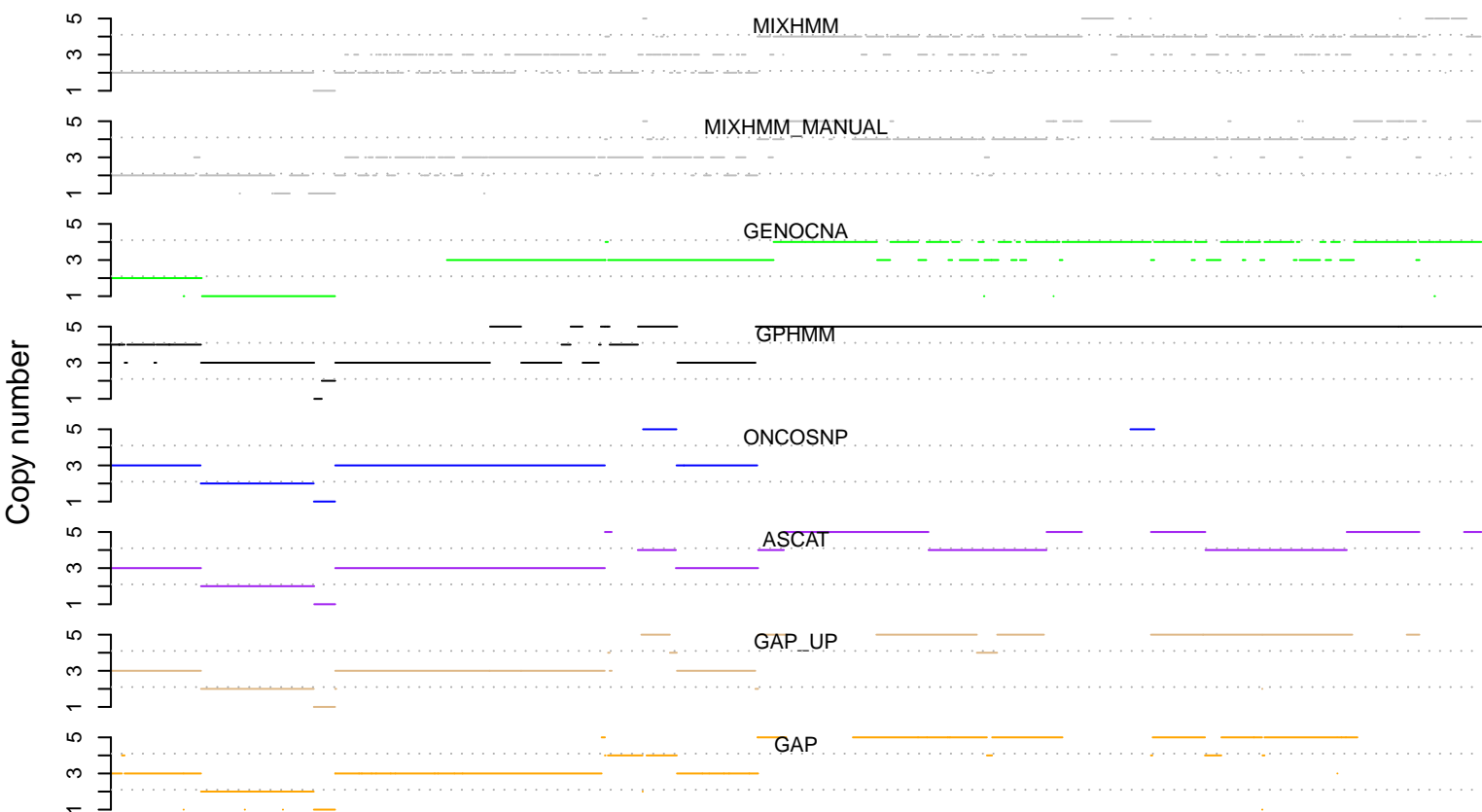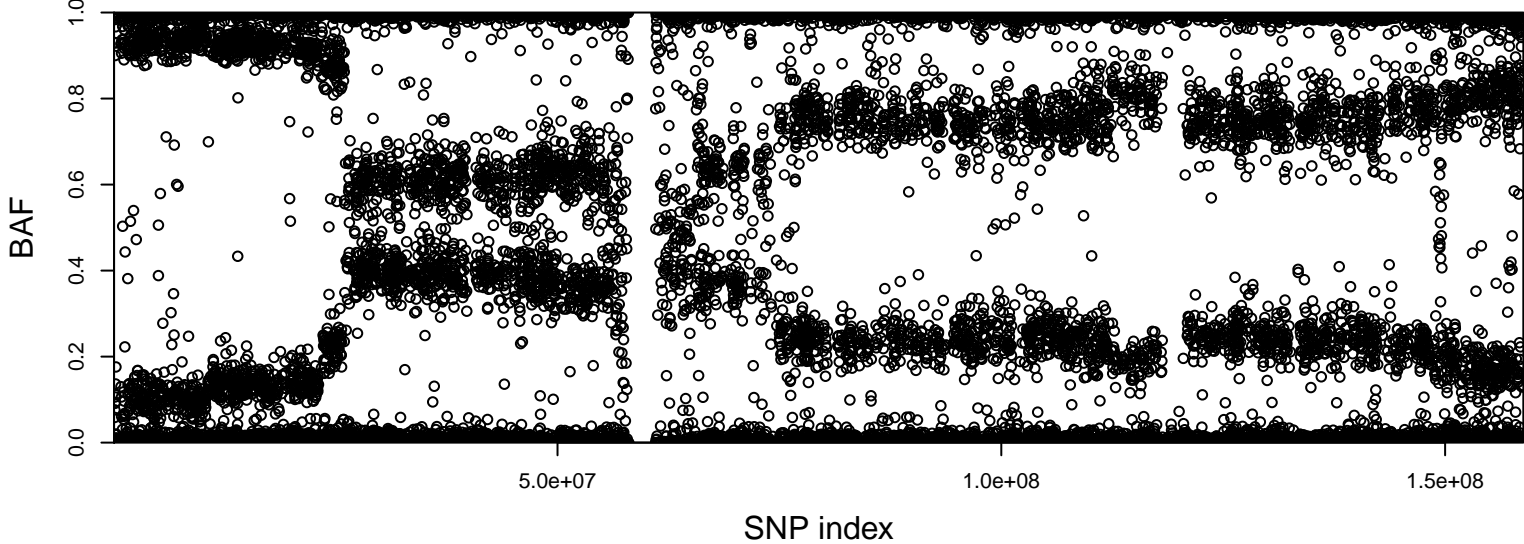

Supplement: Additional file 9 — Cell-line data and method calls. LRR (top graph) and BAF (bottom graph) signals for the cell-line sample at 21% contamination. Chromosomes 6, 16 and X are excluded for the reasons described in the main text. In the middle, the calls made by the seven methods, including MixHMM with manually set global parameters (LRR shift and contamination), and the reference true calls. If any, calls made with copy numbers higher than 4 are displayed as copy number 4. [file 1471-2105-13-192-S9.zip › chr7_calls.pdf]

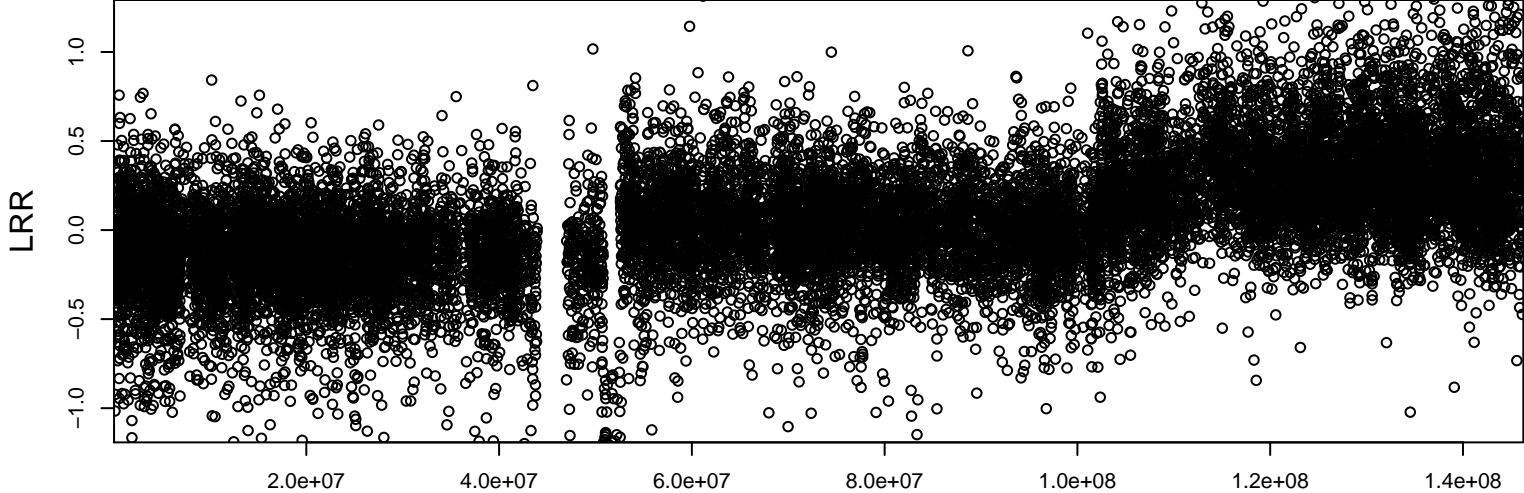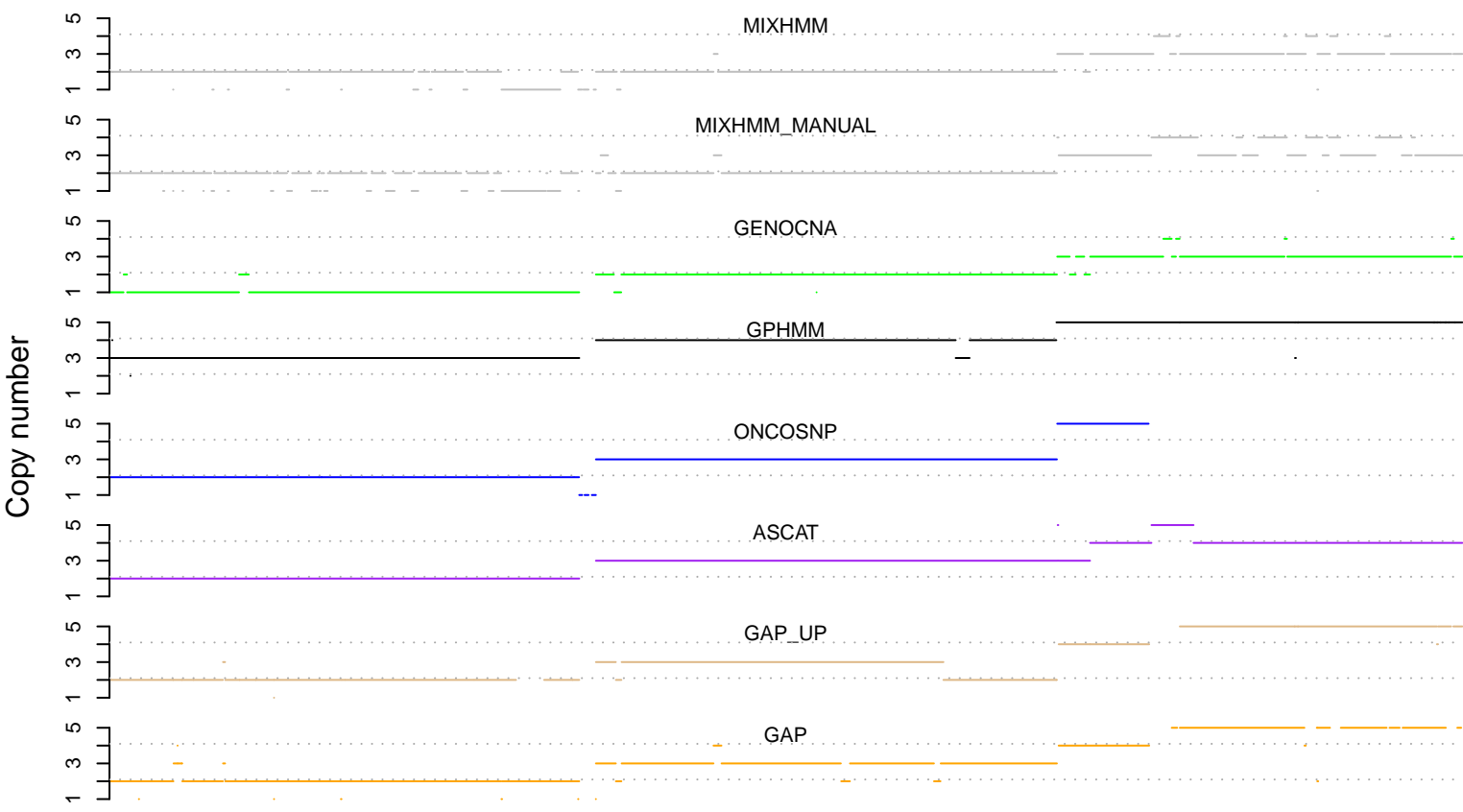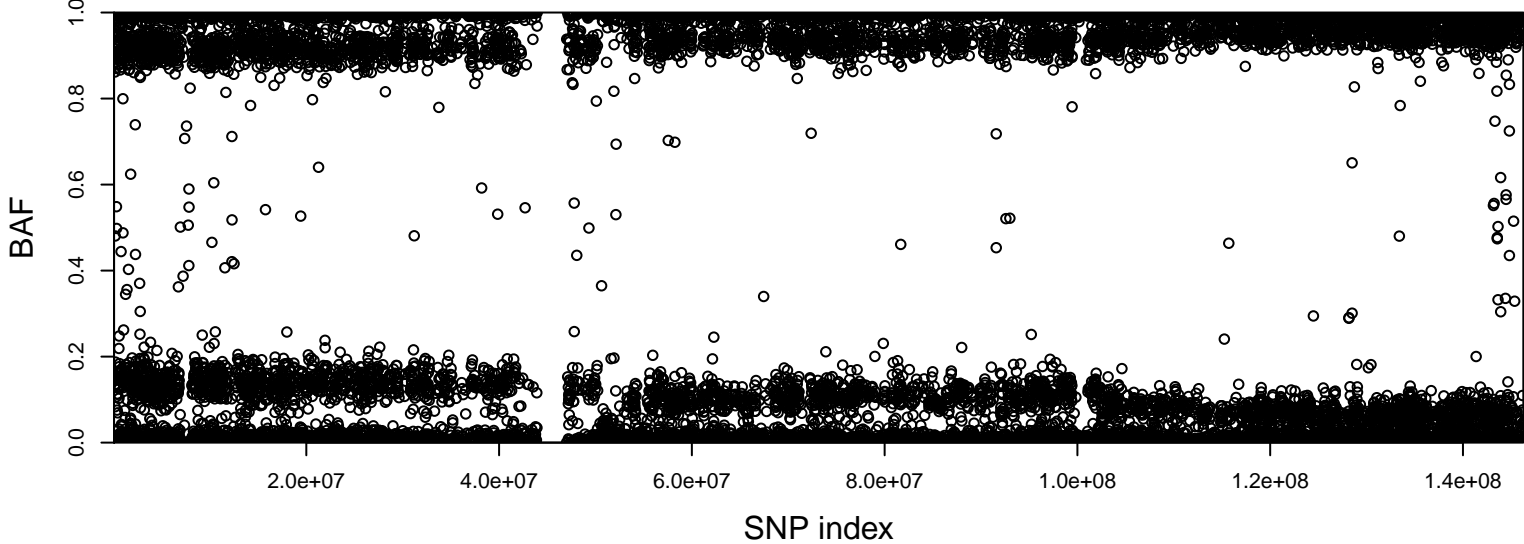

Supplement: Additional file 9 — Cell-line data and method calls. LRR (top graph) and BAF (bottom graph) signals for the cell-line sample at 21% contamination. Chromosomes 6, 16 and X are excluded for the reasons described in the main text. In the middle, the calls made by the seven methods, including MixHMM with manually set global parameters (LRR shift and contamination), and the reference true calls. If any, calls made with copy numbers higher than 4 are displayed as copy number 4. [file 1471-2105-13-192-S9.zip › chr8_calls.pdf]

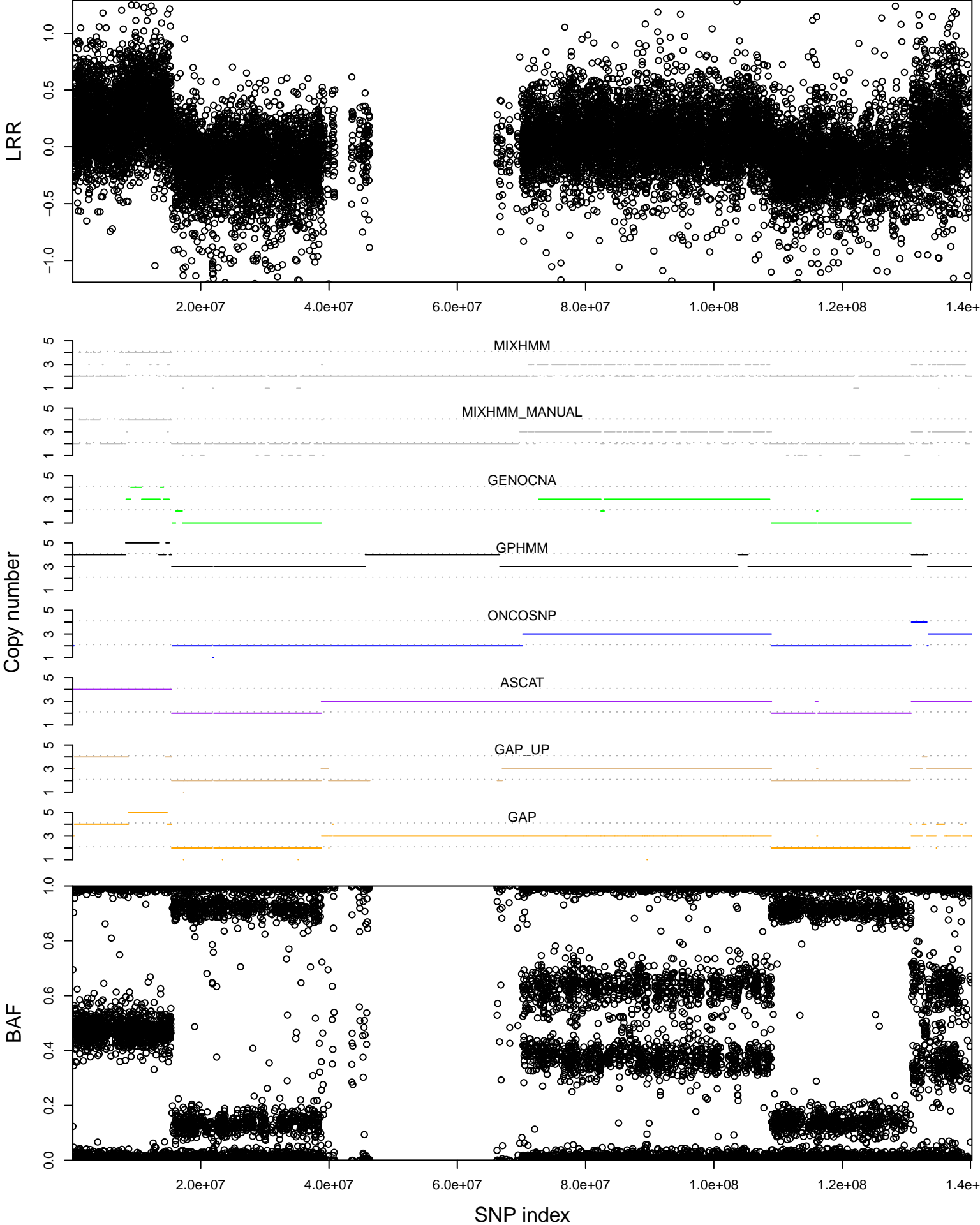

Supplement: Additional file 9 — Cell-line data and method calls. LRR (top graph) and BAF (bottom graph) signals for the cell-line sample at 21% contamination. Chromosomes 6, 16 and X are excluded for the reasons described in the main text. In the middle, the calls made by the seven methods, including MixHMM with manually set global parameters (LRR shift and contamination), and the reference true calls. If any, calls made with copy numbers higher than 4 are displayed as copy number 4. [file 1471-2105-13-192-S9.zip › chr9_calls.pdf]

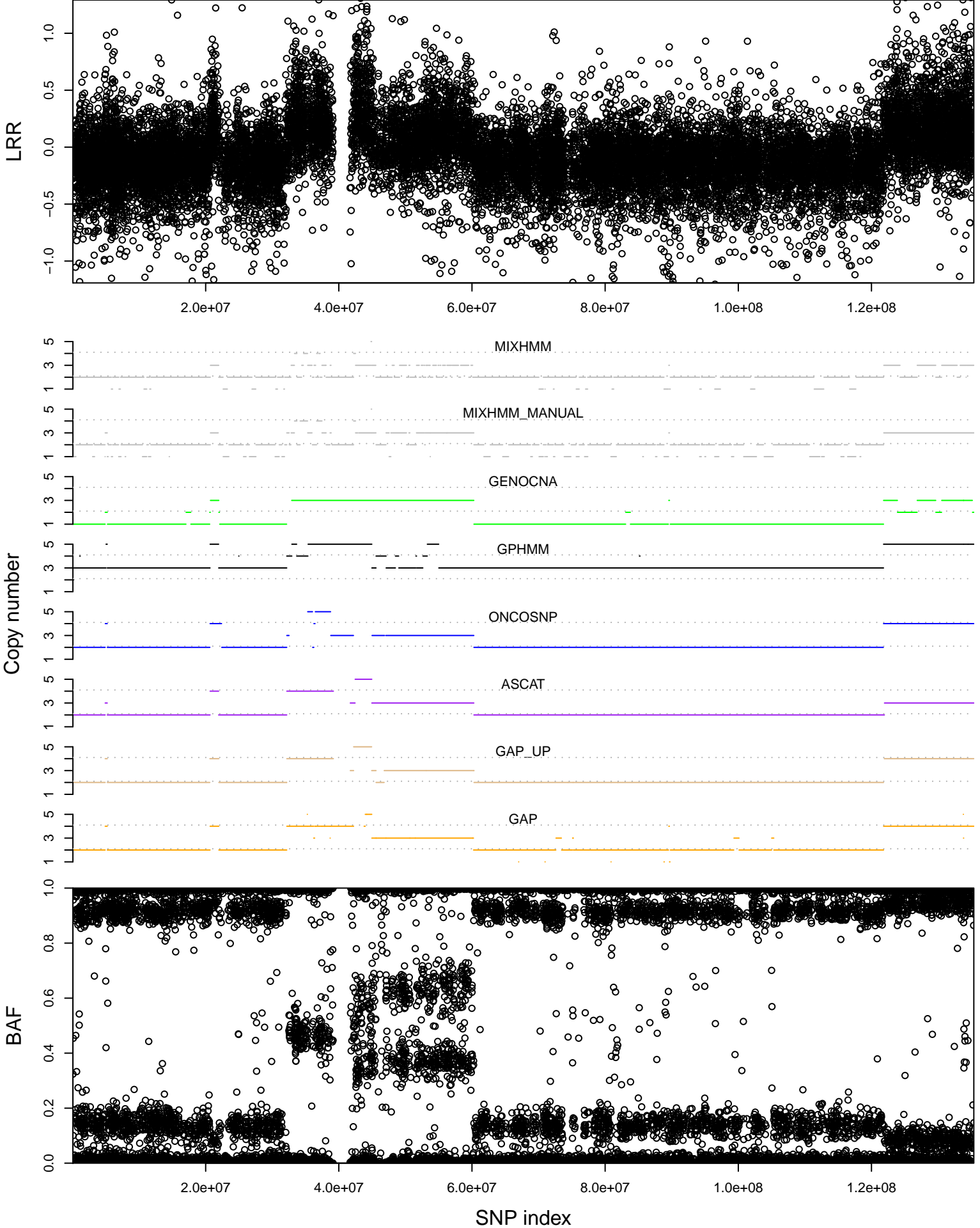

Supplement: Additional file 9 — Cell-line data and method calls. LRR (top graph) and BAF (bottom graph) signals for the cell-line sample at 21% contamination. Chromosomes 6, 16 and X are excluded for the reasons described in the main text. In the middle, the calls made by the seven methods, including MixHMM with manually set global parameters (LRR shift and contamination), and the reference true calls. If any, calls made with copy numbers higher than 4 are displayed as copy number 4. [file 1471-2105-13-192-S9.zip › chr10_calls.pdf]

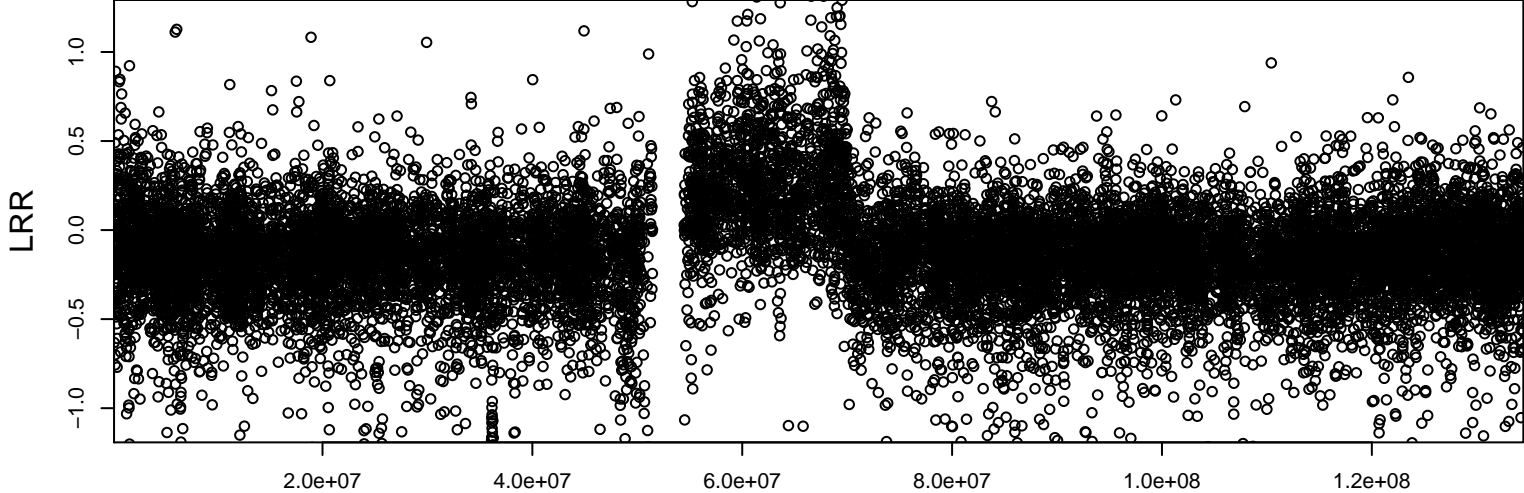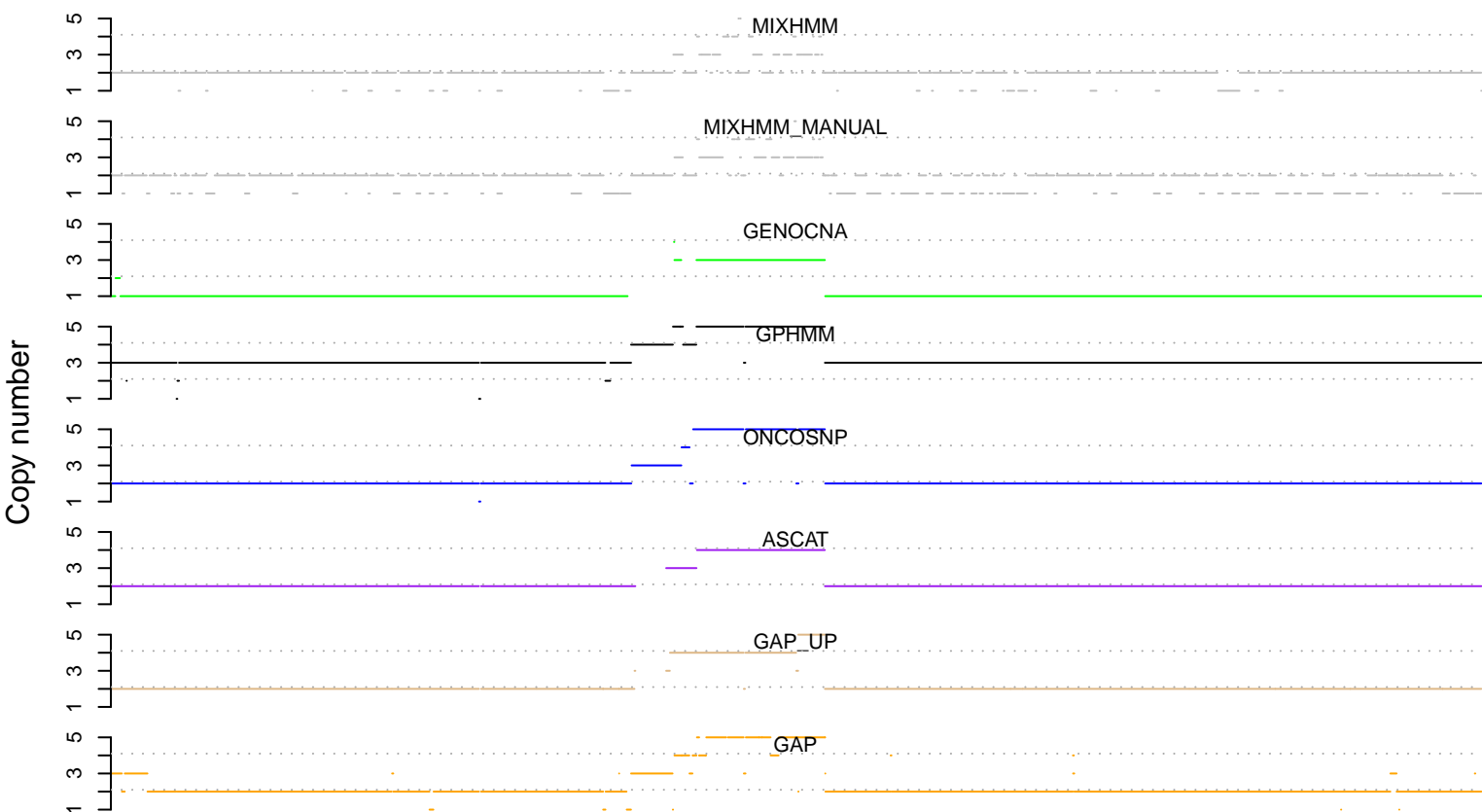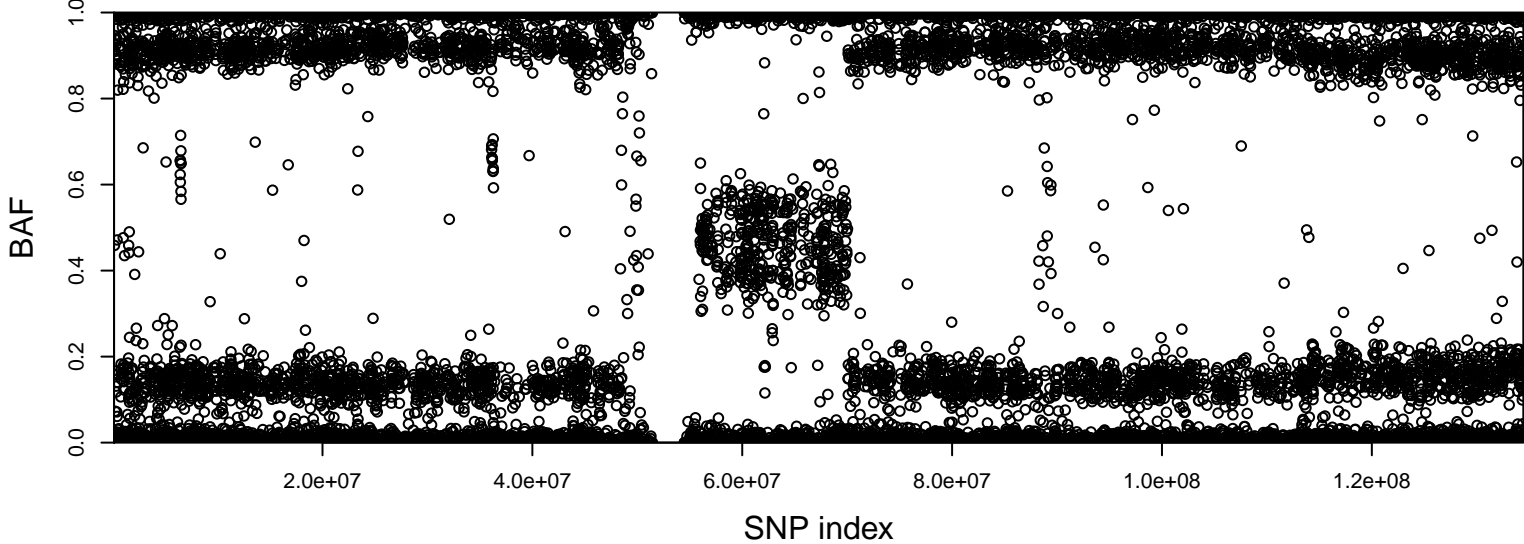

Supplement: Additional file 9 — Cell-line data and method calls. LRR (top graph) and BAF (bottom graph) signals for the cell-line sample at 21% contamination. Chromosomes 6, 16 and X are excluded for the reasons described in the main text. In the middle, the calls made by the seven methods, including MixHMM with manually set global parameters (LRR shift and contamination), and the reference true calls. If any, calls made with copy numbers higher than 4 are displayed as copy number 4. [file 1471-2105-13-192-S9.zip › chr11_calls.pdf]

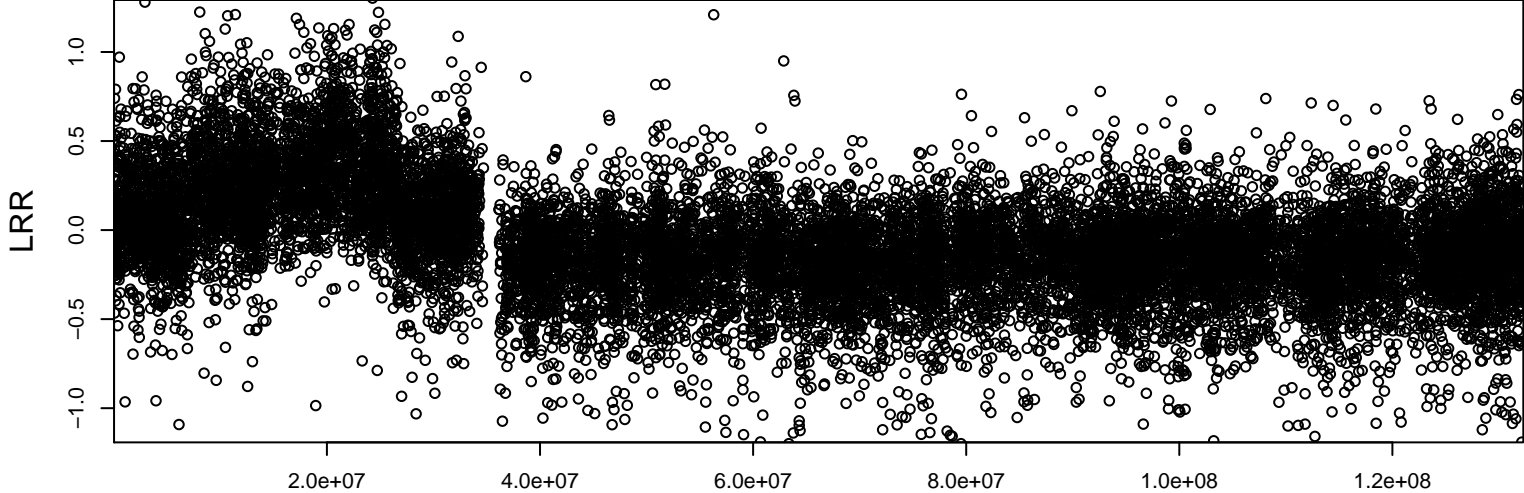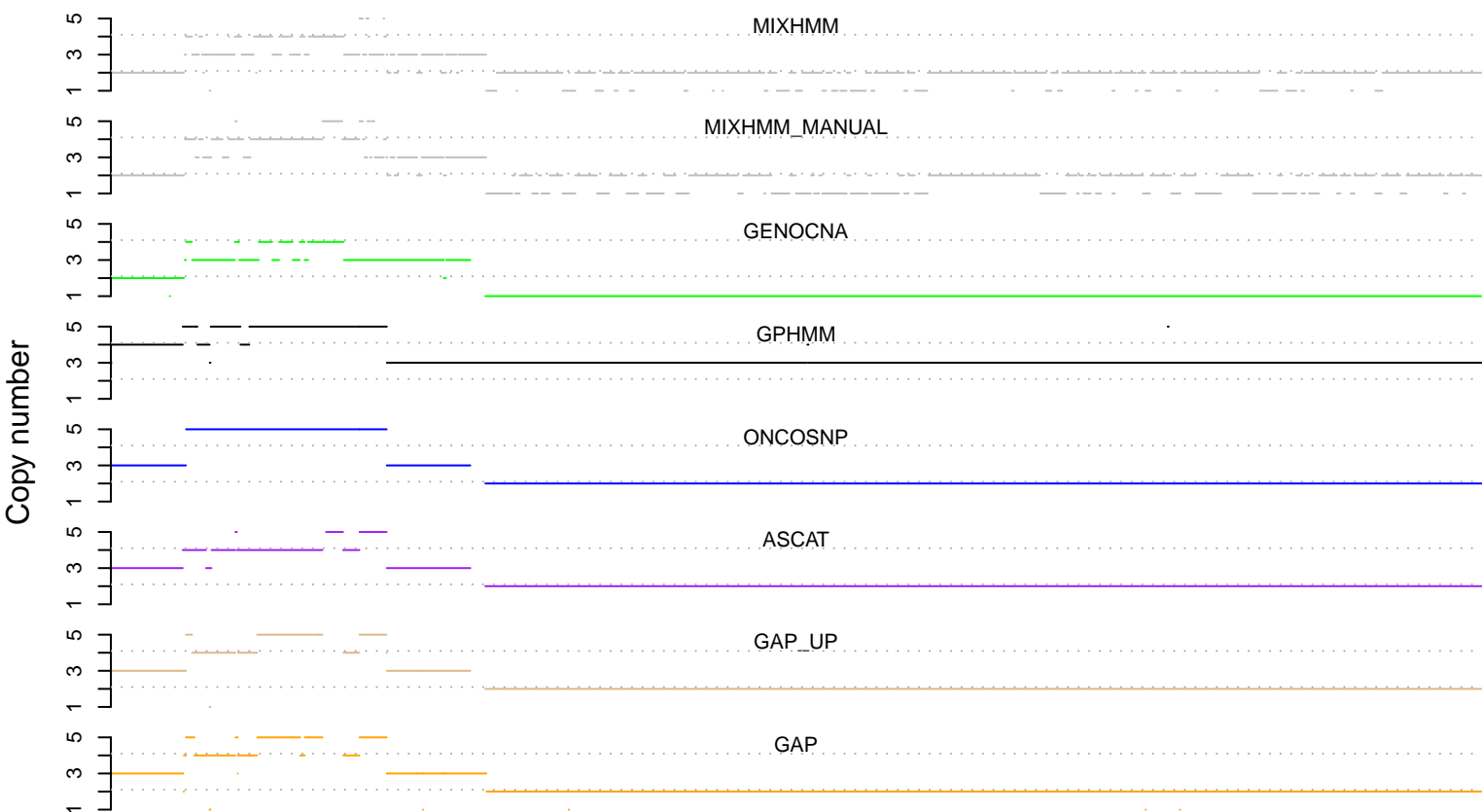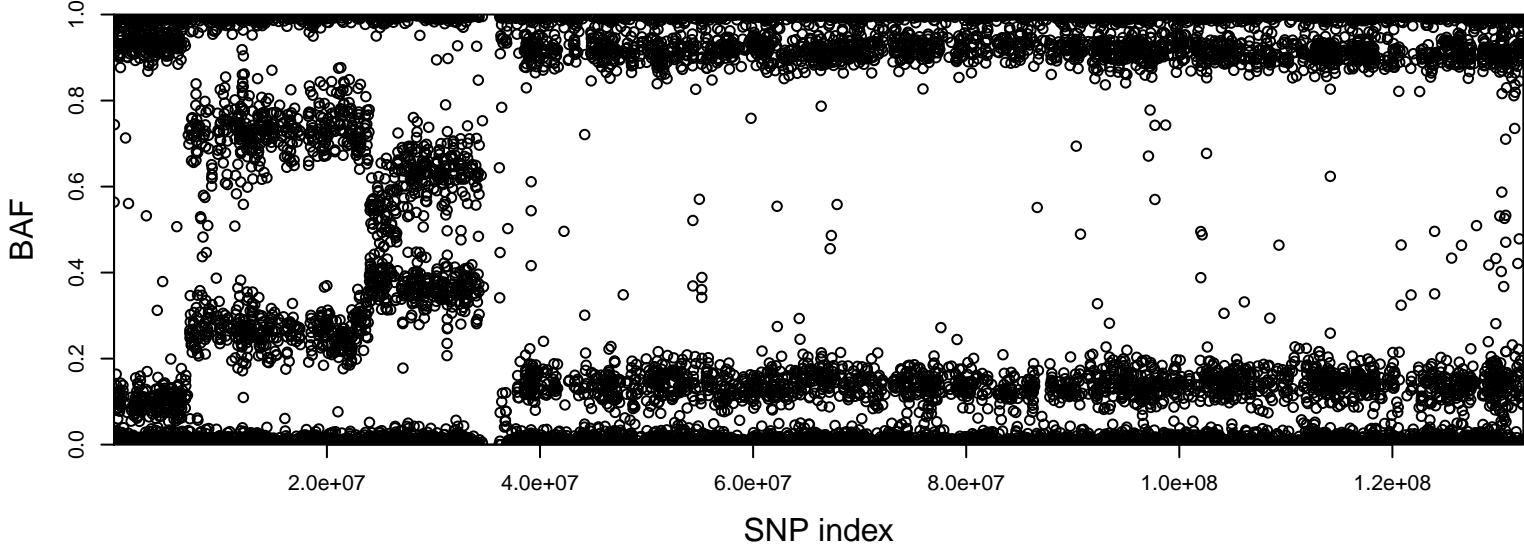

Supplement: Additional file 9 — Cell-line data and method calls. LRR (top graph) and BAF (bottom graph) signals for the cell-line sample at 21% contamination. Chromosomes 6, 16 and X are excluded for the reasons described in the main text. In the middle, the calls made by the seven methods, including MixHMM with manually set global parameters (LRR shift and contamination), and the reference true calls. If any, calls made with copy numbers higher than 4 are displayed as copy number 4. [file 1471-2105-13-192-S9.zip › chr12_calls.pdf]

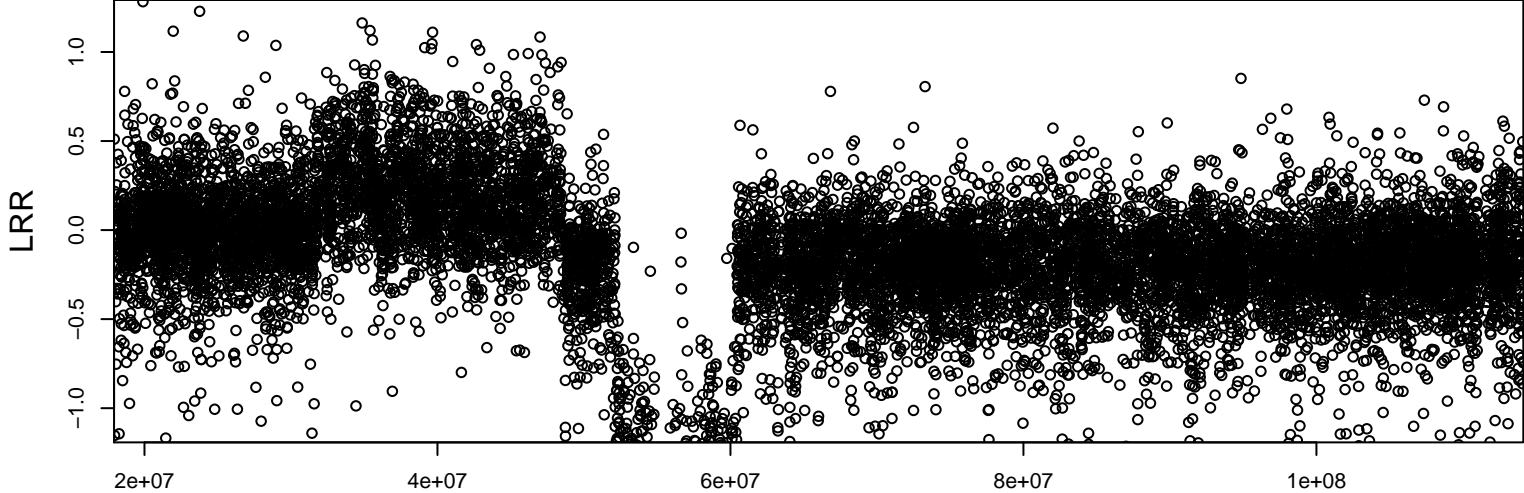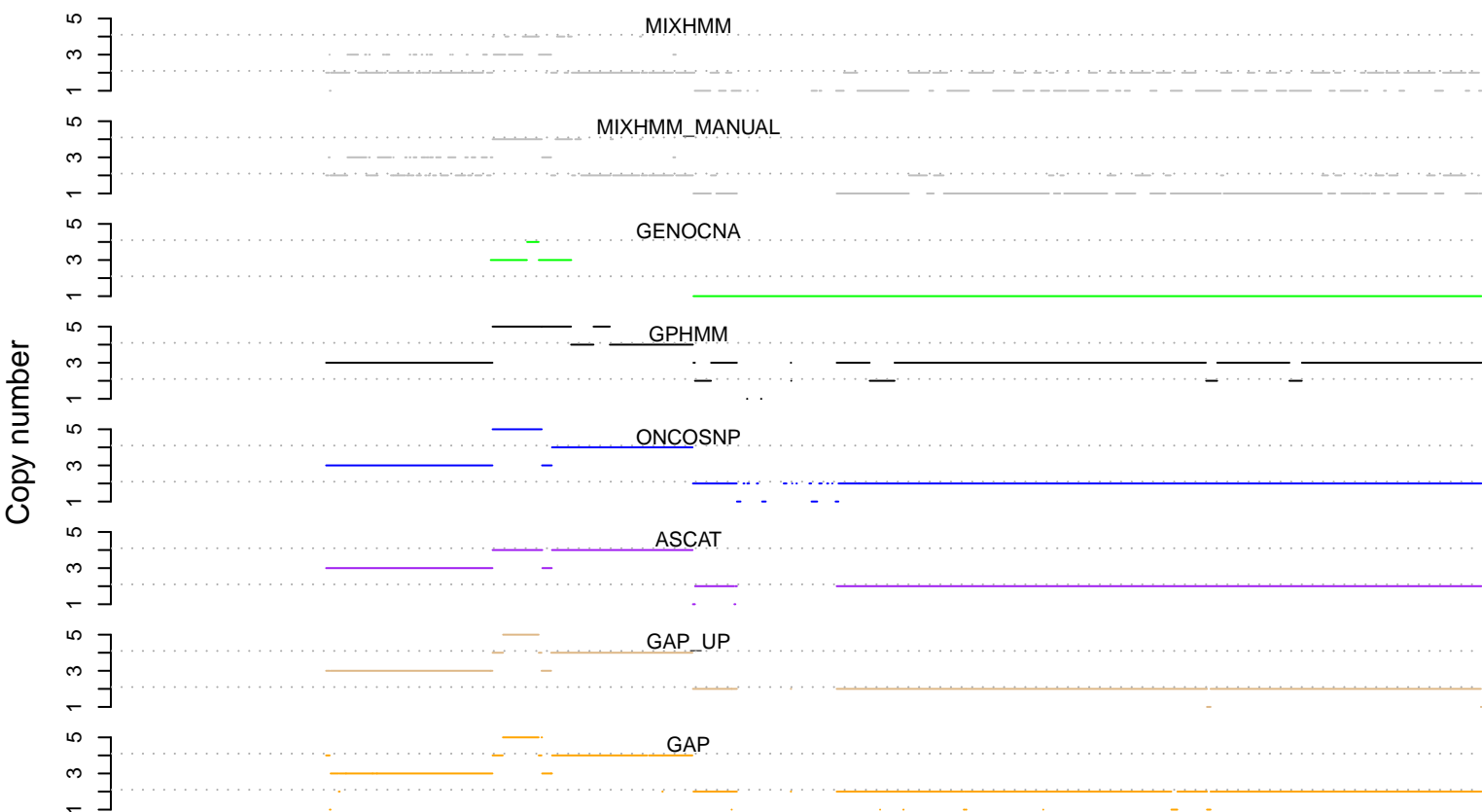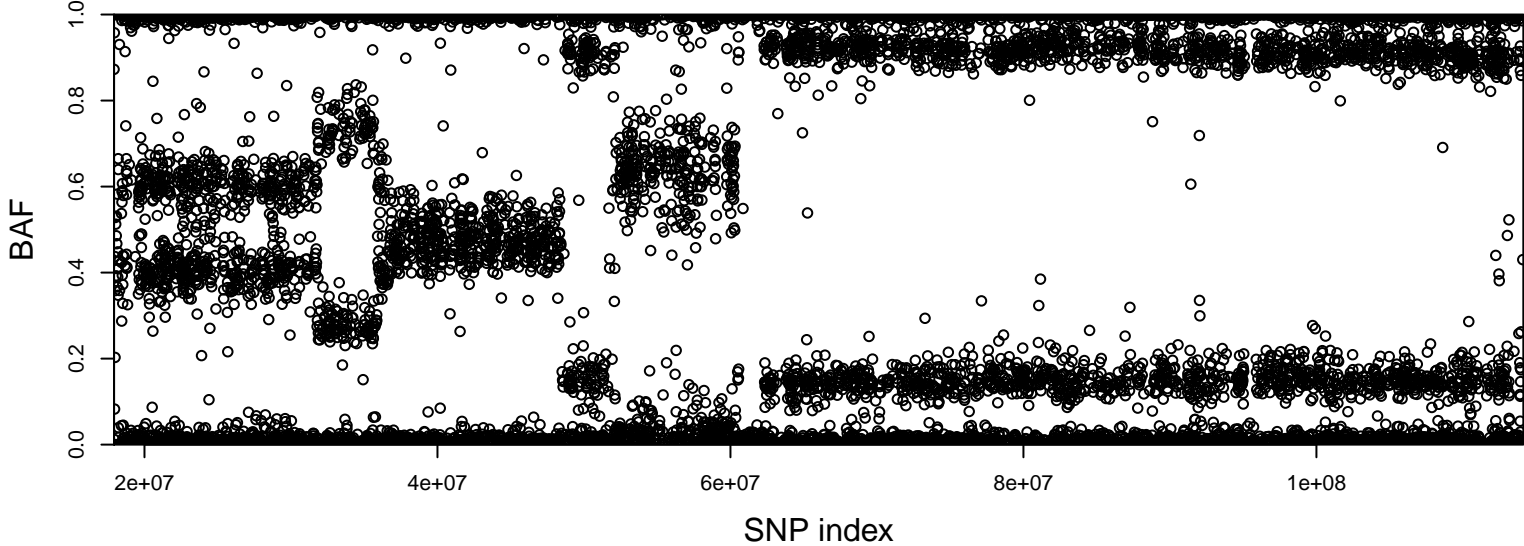

Supplement: Additional file 9 — Cell-line data and method calls. LRR (top graph) and BAF (bottom graph) signals for the cell-line sample at 21% contamination. Chromosomes 6, 16 and X are excluded for the reasons described in the main text. In the middle, the calls made by the seven methods, including MixHMM with manually set global parameters (LRR shift and contamination), and the reference true calls. If any, calls made with copy numbers higher than 4 are displayed as copy number 4. [file 1471-2105-13-192-S9.zip › chr13_calls.pdf]

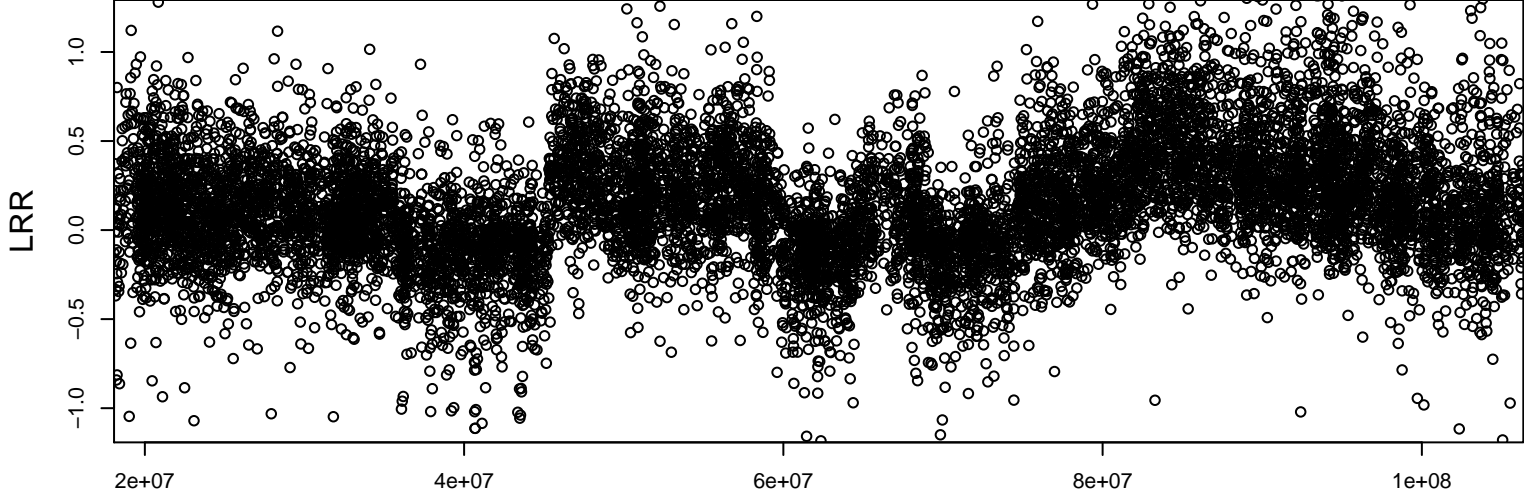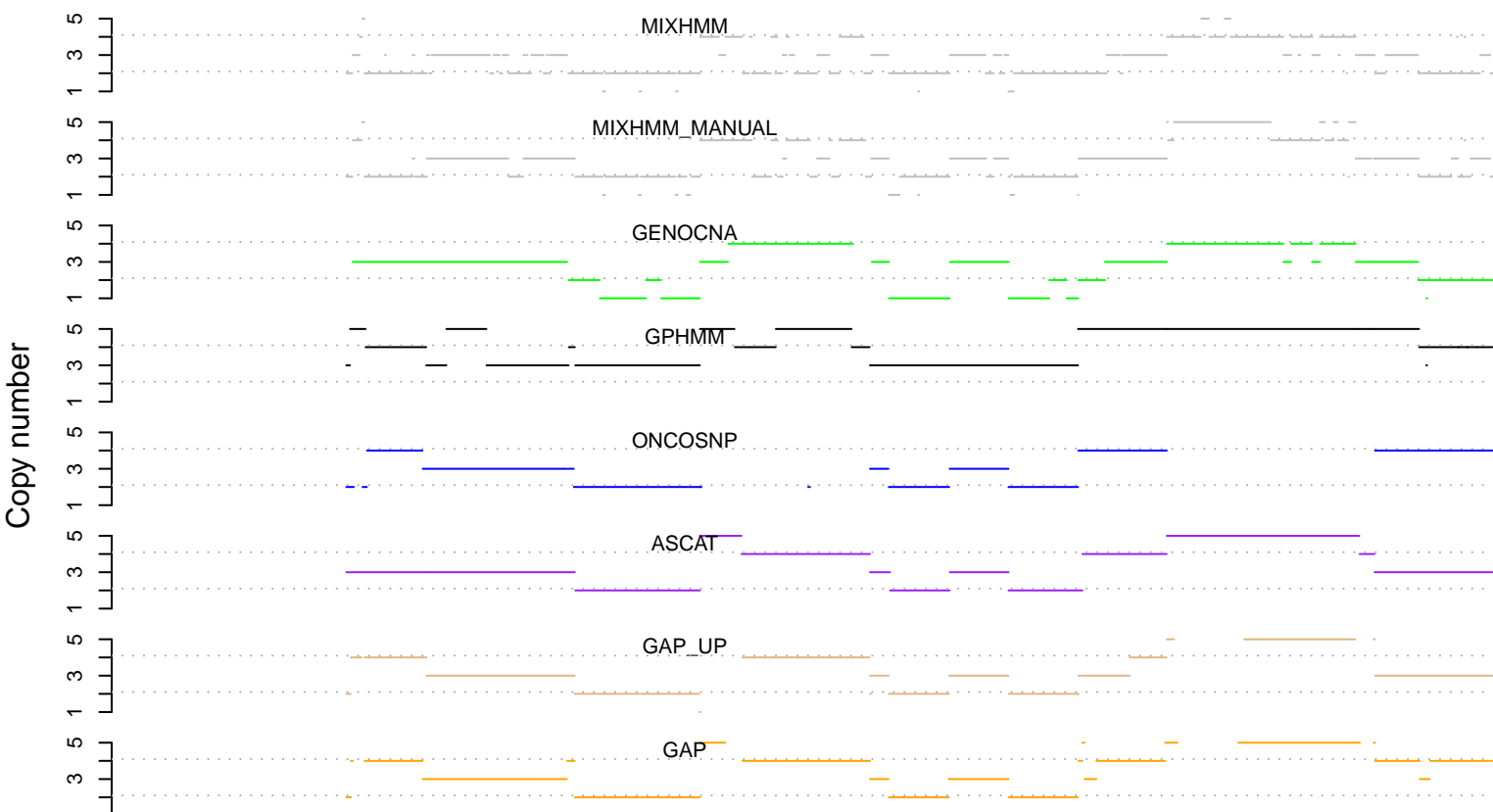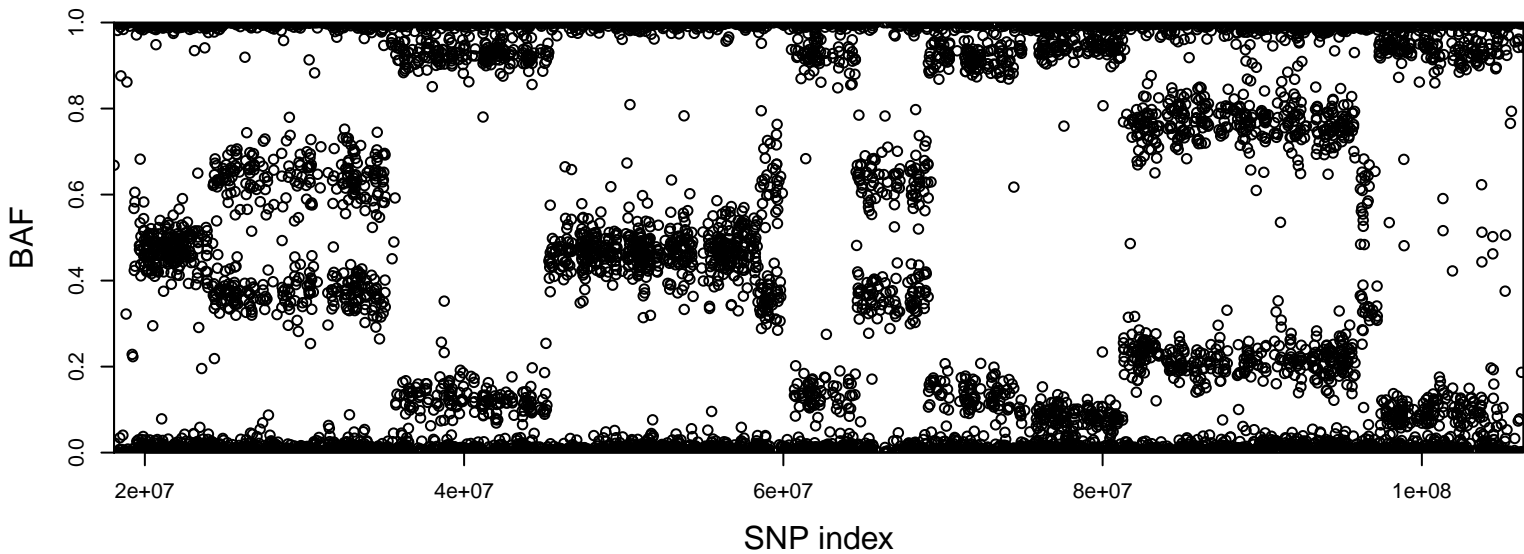

Supplement: Additional file 9 — Cell-line data and method calls. LRR (top graph) and BAF (bottom graph) signals for the cell-line sample at 21% contamination. Chromosomes 6, 16 and X are excluded for the reasons described in the main text. In the middle, the calls made by the seven methods, including MixHMM with manually set global parameters (LRR shift and contamination), and the reference true calls. If any, calls made with copy numbers higher than 4 are displayed as copy number 4. [file 1471-2105-13-192-S9.zip › chr14_calls.pdf]

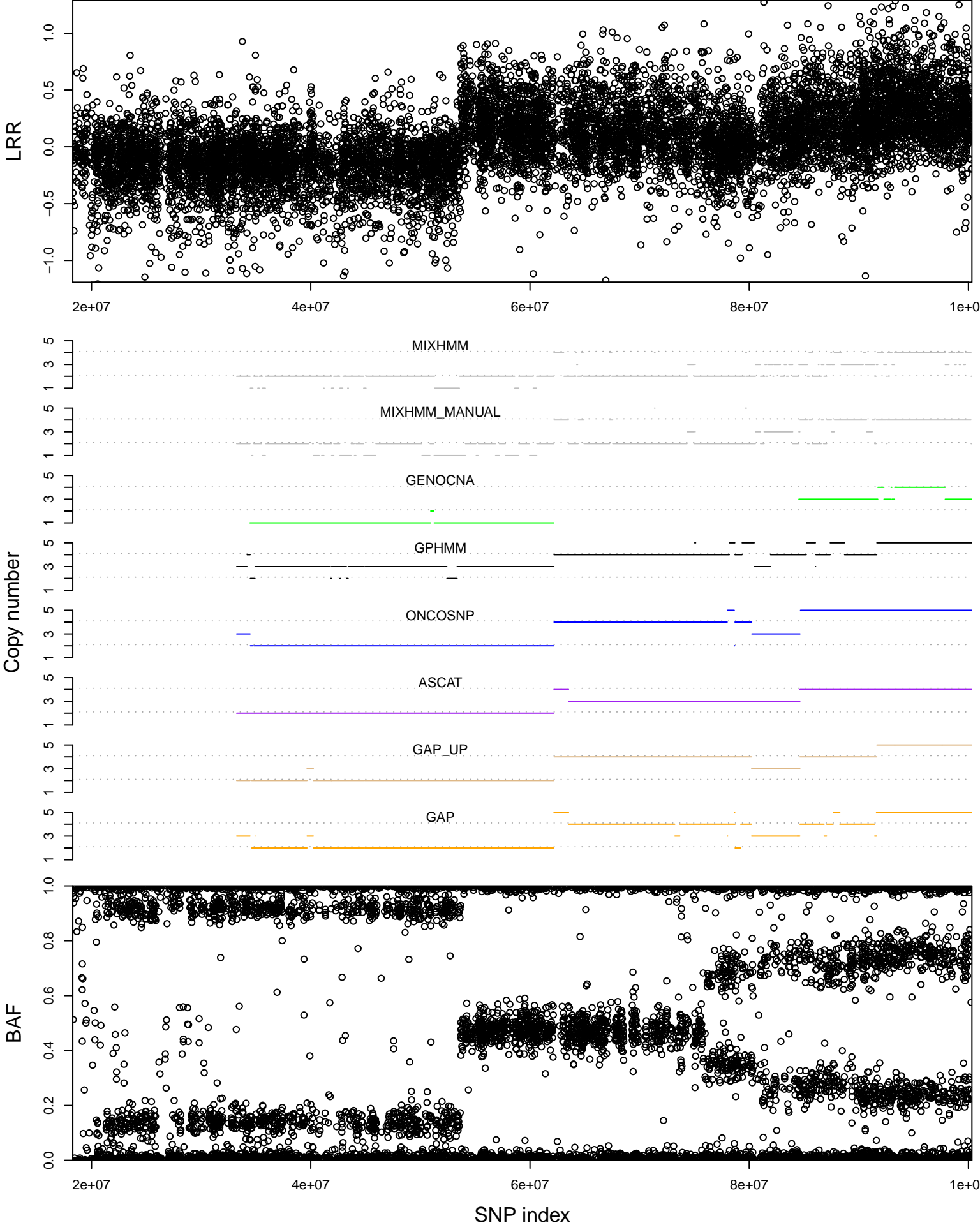

Supplement: Additional file 9 — Cell-line data and method calls. LRR (top graph) and BAF (bottom graph) signals for the cell-line sample at 21% contamination. Chromosomes 6, 16 and X are excluded for the reasons described in the main text. In the middle, the calls made by the seven methods, including MixHMM with manually set global parameters (LRR shift and contamination), and the reference true calls. If any, calls made with copy numbers higher than 4 are displayed as copy number 4. [file 1471-2105-13-192-S9.zip › chr15_calls.pdf]

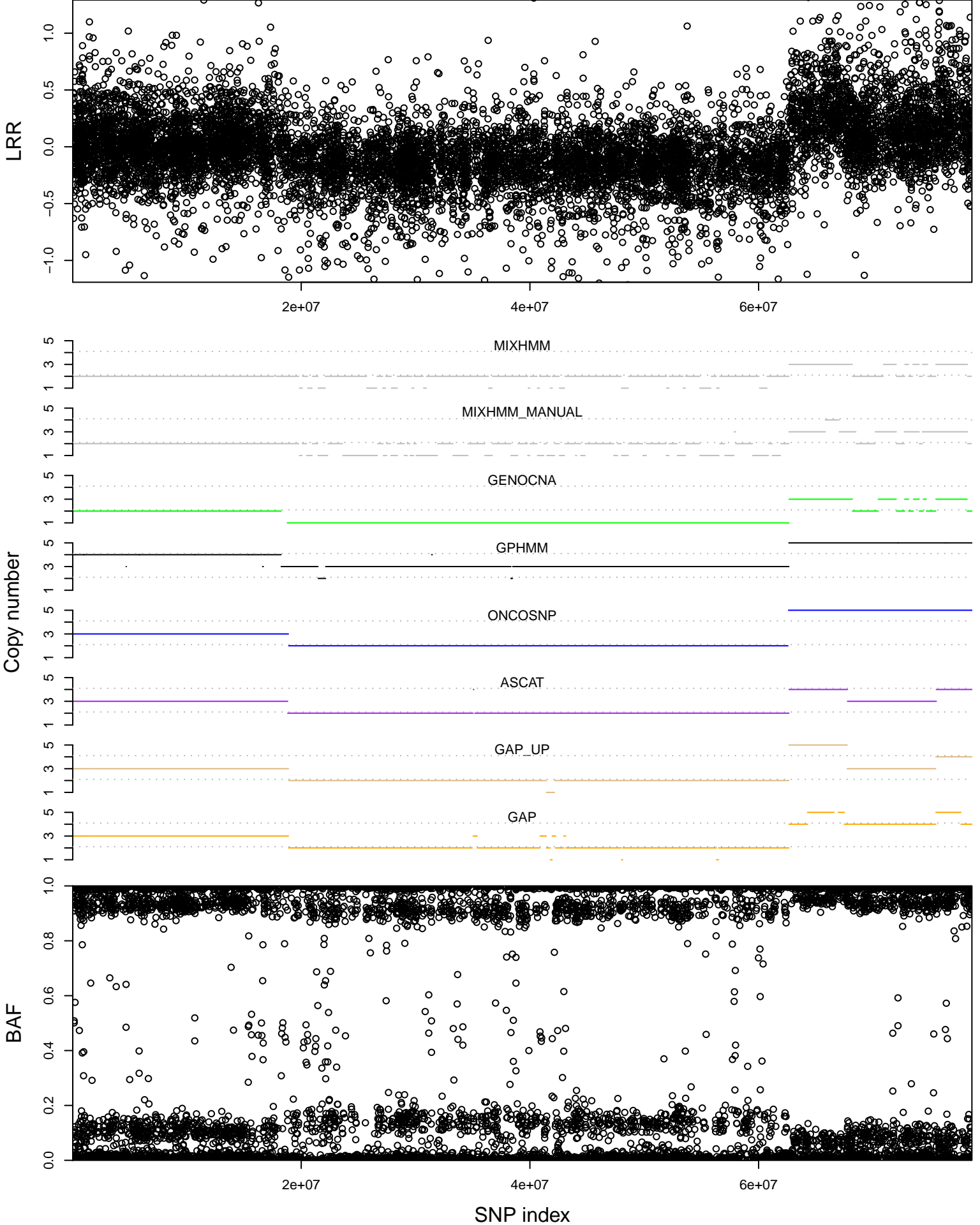

Supplement: Additional file 9 — Cell-line data and method calls. LRR (top graph) and BAF (bottom graph) signals for the cell-line sample at 21% contamination. Chromosomes 6, 16 and X are excluded for the reasons described in the main text. In the middle, the calls made by the seven methods, including MixHMM with manually set global parameters (LRR shift and contamination), and the reference true calls. If any, calls made with copy numbers higher than 4 are displayed as copy number 4. [file 1471-2105-13-192-S9.zip › chr17_calls.pdf]

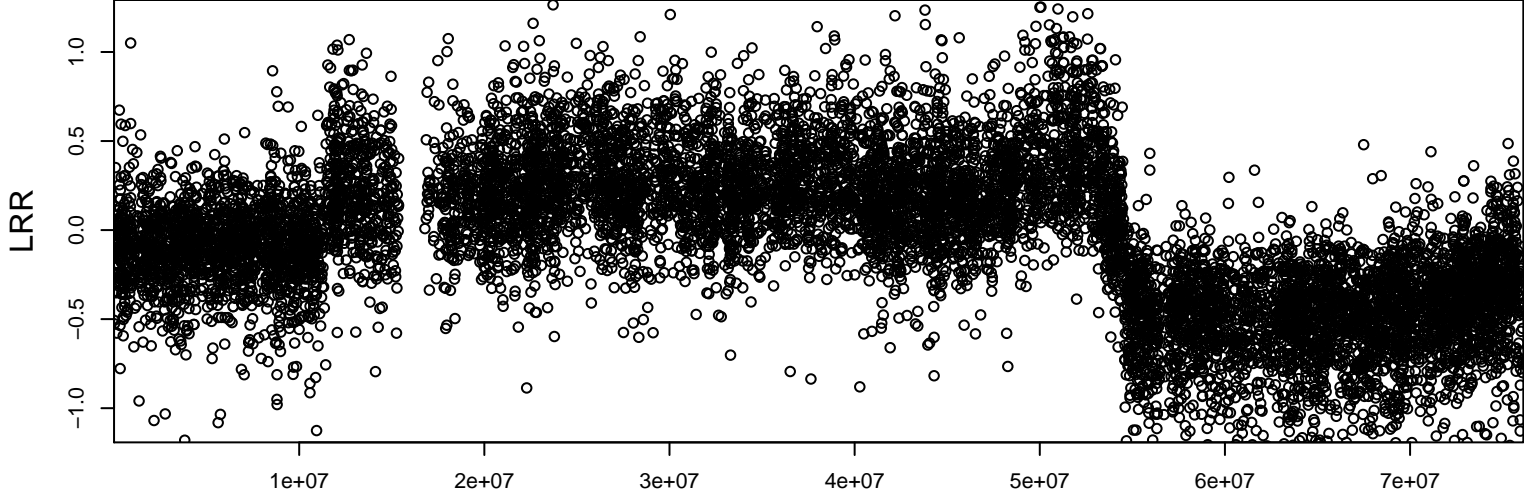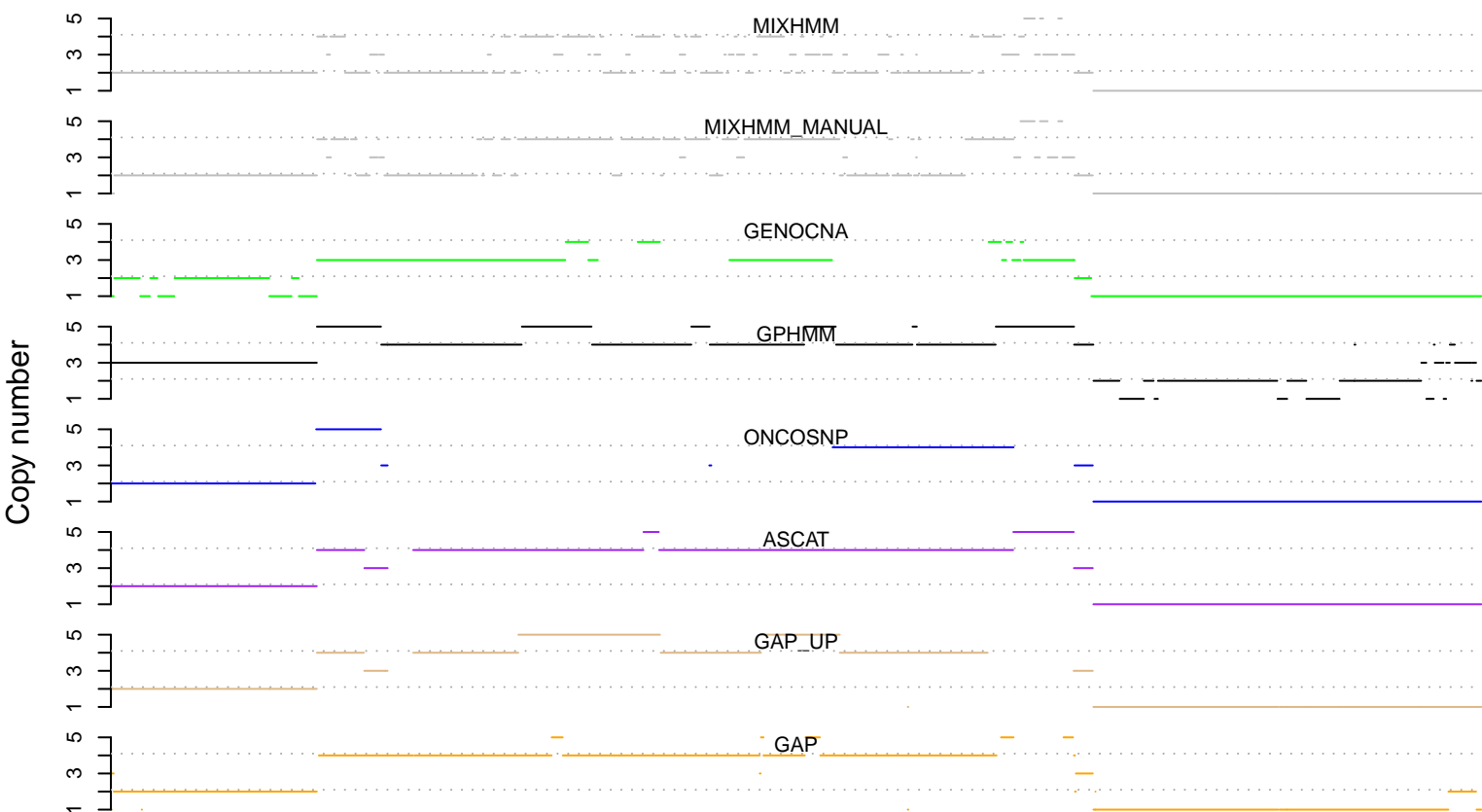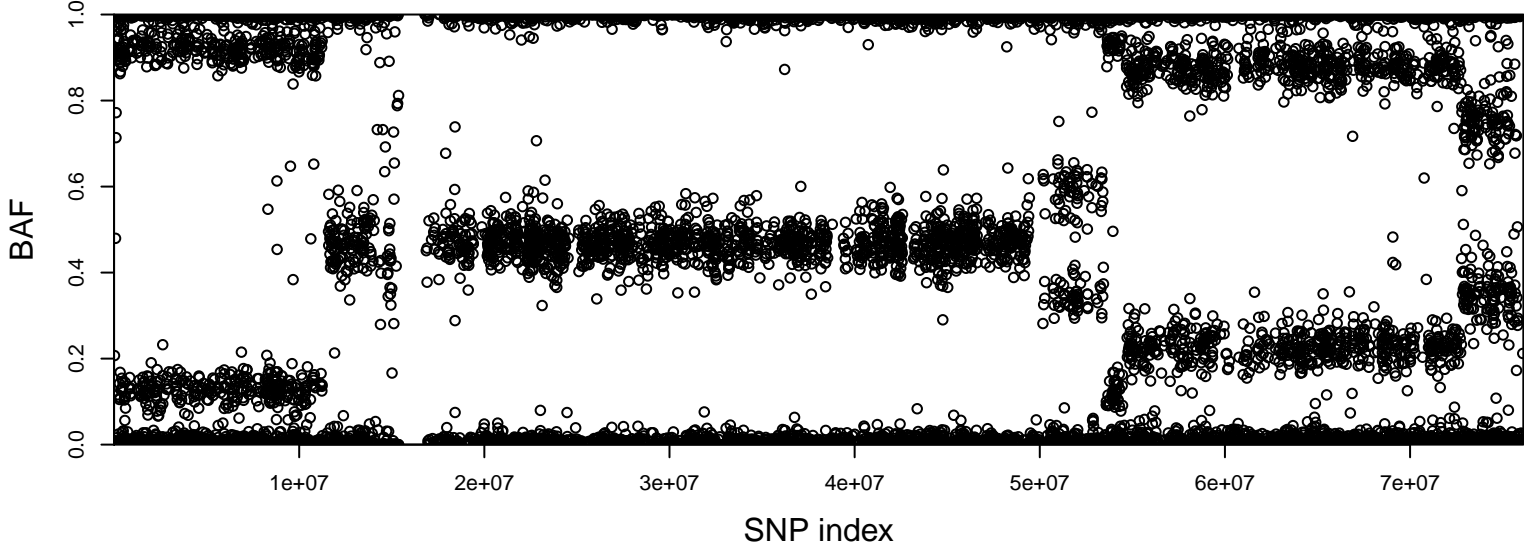

Supplement: Additional file 9 — Cell-line data and method calls. LRR (top graph) and BAF (bottom graph) signals for the cell-line sample at 21% contamination. Chromosomes 6, 16 and X are excluded for the reasons described in the main text. In the middle, the calls made by the seven methods, including MixHMM with manually set global parameters (LRR shift and contamination), and the reference true calls. If any, calls made with copy numbers higher than 4 are displayed as copy number 4. [file 1471-2105-13-192-S9.zip › chr18_calls.pdf]

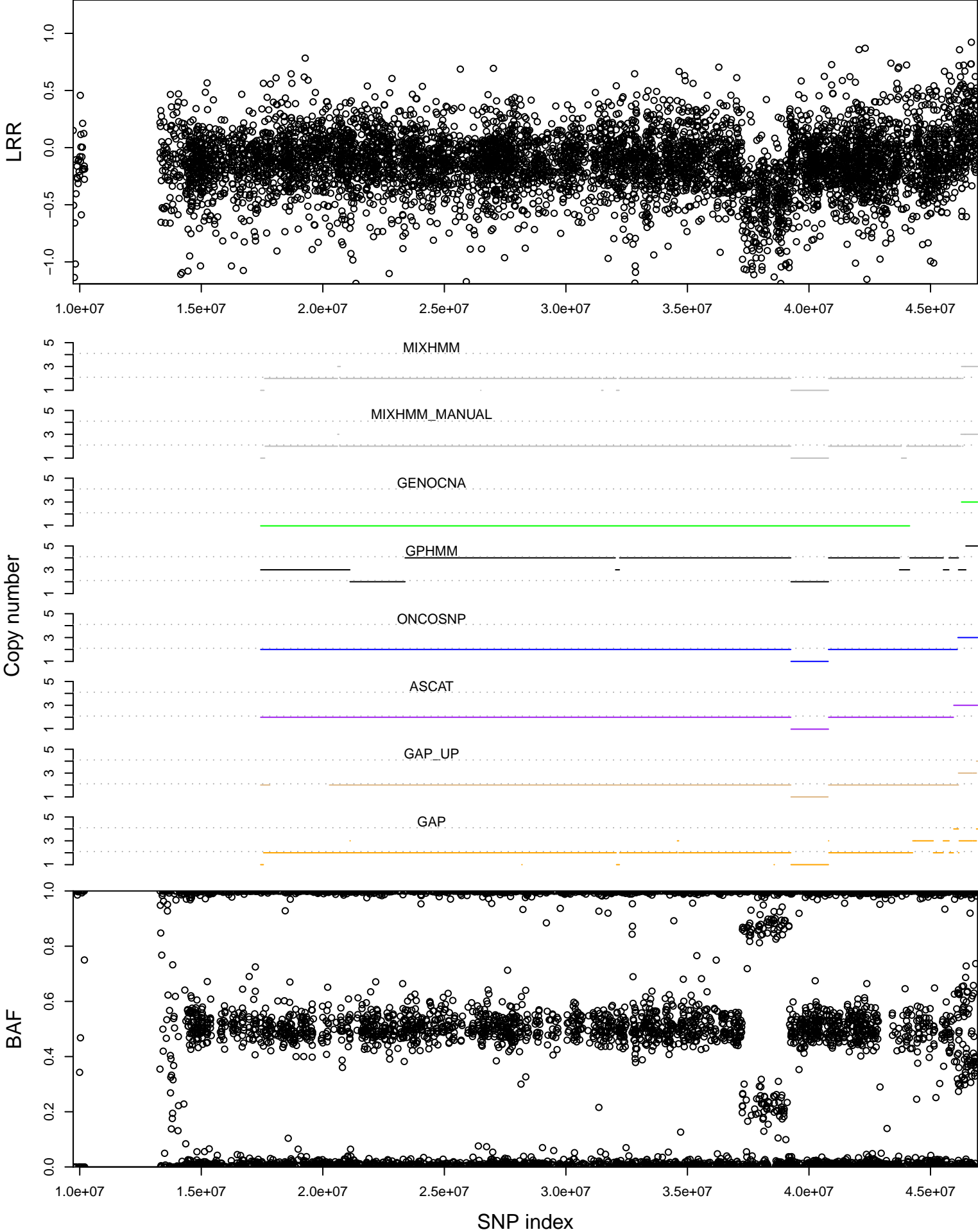

Supplement: Additional file 9 — Cell-line data and method calls. LRR (top graph) and BAF (bottom graph) signals for the cell-line sample at 21% contamination. Chromosomes 6, 16 and X are excluded for the reasons described in the main text. In the middle, the calls made by the seven methods, including MixHMM with manually set global parameters (LRR shift and contamination), and the reference true calls. If any, calls made with copy numbers higher than 4 are displayed as copy number 4. [file 1471-2105-13-192-S9.zip › chr21_calls.pdf]

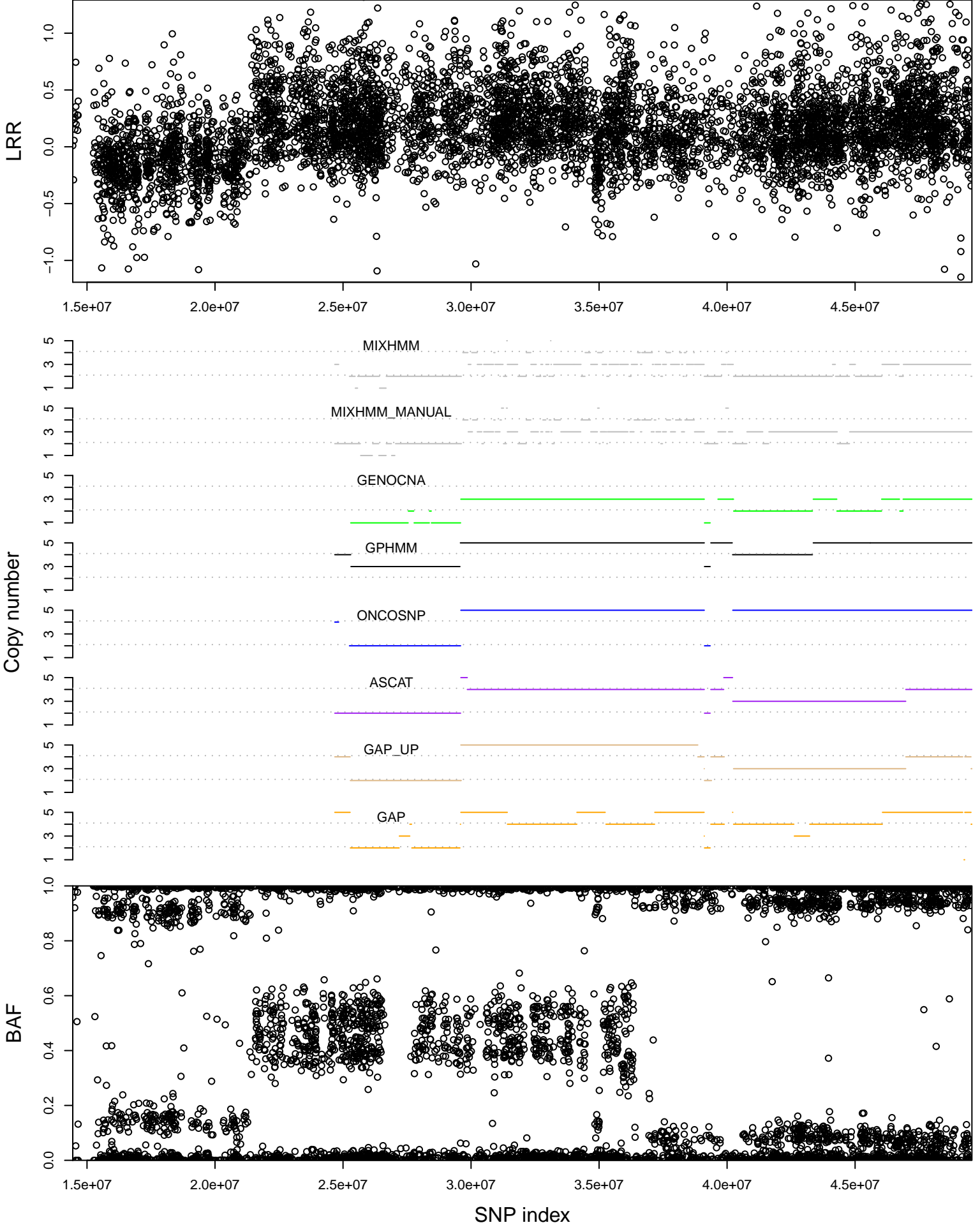

Supplement: Additional file 9 — Cell-line data and method calls. LRR (top graph) and BAF (bottom graph) signals for the cell-line sample at 21% contamination. Chromosomes 6, 16 and X are excluded for the reasons described in the main text. In the middle, the calls made by the seven methods, including MixHMM with manually set global parameters (LRR shift and contamination), and the reference true calls. If any, calls made with copy numbers higher than 4 are displayed as copy number 4. [file 1471-2105-13-192-S9.zip › chr22_calls.pdf]

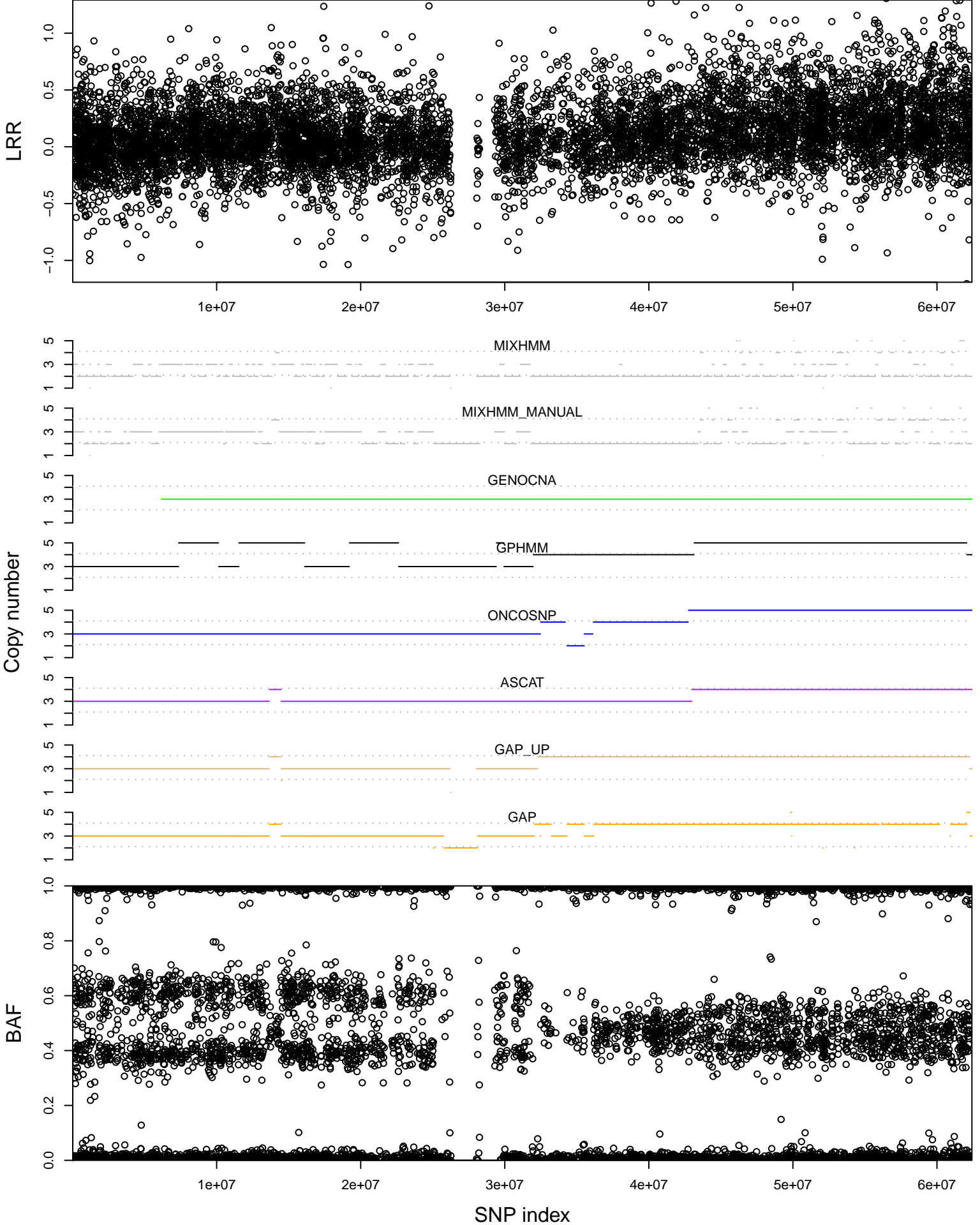

Supplement: Additional file 9 — Cell-line data and method calls. LRR (top graph) and BAF (bottom graph) signals for the cell-line sample at 21% contamination. Chromosomes 6, 16 and X are excluded for the reasons described in the main text. In the middle, the calls made by the seven methods, including MixHMM with manually set global parameters (LRR shift and contamination), and the reference true calls. If any, calls made with copy numbers higher than 4 are displayed as copy number 4. [file 1471-2105-13-192-S9.zip › chr20_calls.pdf]

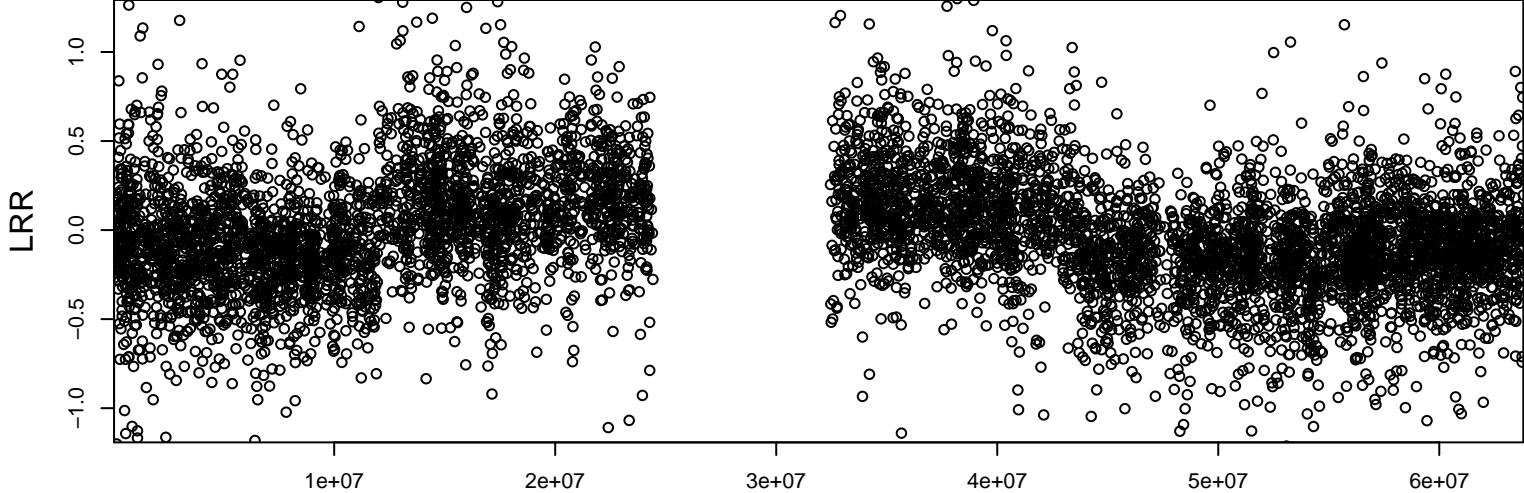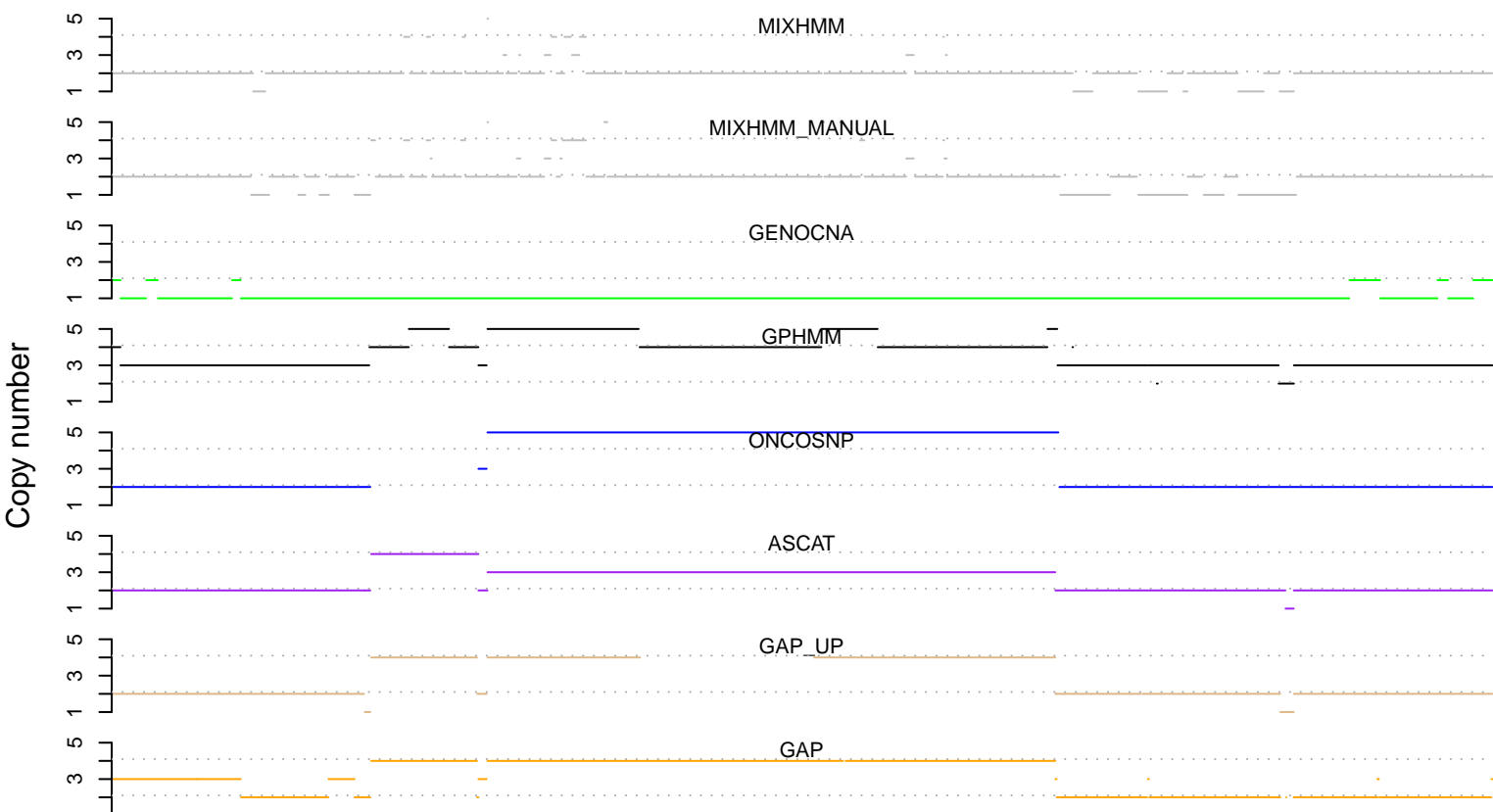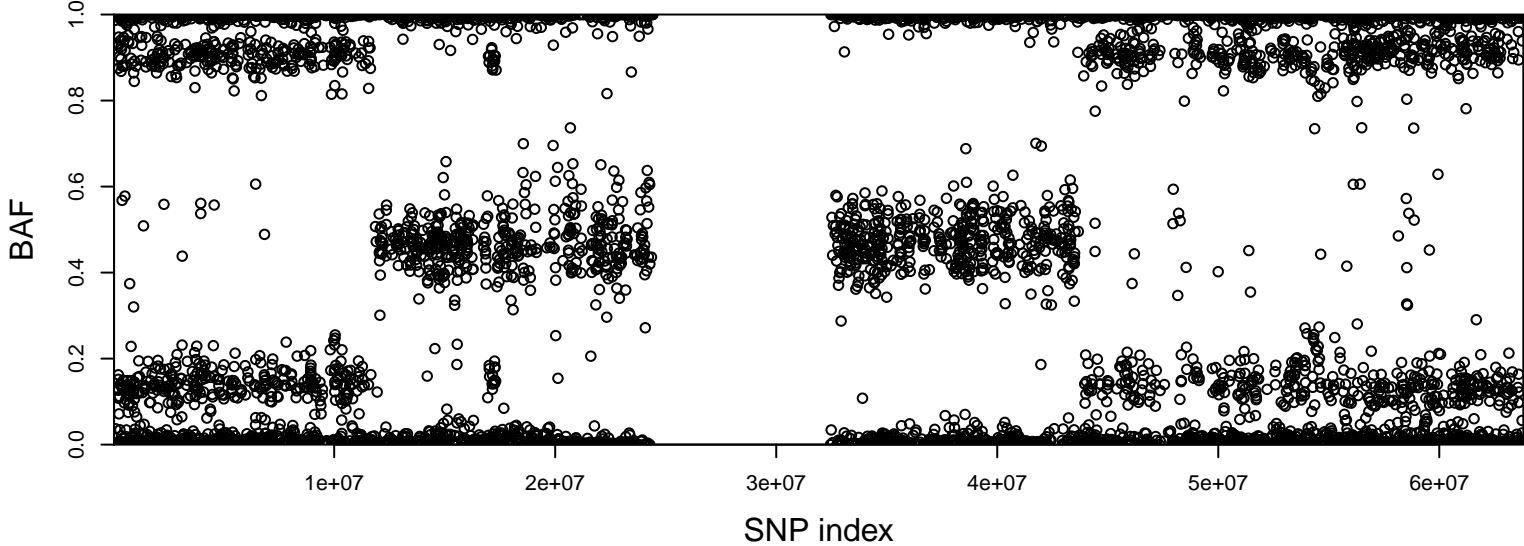

Supplement: Additional file 9 — Cell-line data and method calls. LRR (top graph) and BAF (bottom graph) signals for the cell-line sample at 21% contamination. Chromosomes 6, 16 and X are excluded for the reasons described in the main text. In the middle, the calls made by the seven methods, including MixHMM with manually set global parameters (LRR shift and contamination), and the reference true calls. If any, calls made with copy numbers higher than 4 are displayed as copy number 4. [file 1471-2105-13-192-S9.zip › chr19_calls.pdf]
