# Supplementary material for: Comparison of methods to detect copy number alterations in cancer using simulated and real genotyping data
Source: BMC Bioinformatics. 2012 Aug 7;13:192. doi: 10.1186/1471-2105-13-192 (PMC3472297; doi:10.1186/1471-2105-13-192)
Supplement: Additional file 5 — Method version and parameterization details. Versions of the methods used in this study and details of parameterization when the default parameters were not used. Additionally, details on the PFB and GC content files used as input when required. [file 1471-2105-13-192-S5.doc]

**Versions**

*ASCAT*: version 2.1 (released on the 15th of January, 2012)

http://heim.ifi.uio.no/bioinf/Projects/ASCAT/

*GAP*: version released on the 15th of November, 2009

http://bioinfo-out.curie.fr/projects/snp_gap/

*GenoCNA*: version 1.09 (released on the 22nd of February, 2010)

http://www.bios.unc.edu/~wsun/software/genoCN.htm

*GPHMM*: executable built on 2nd of October, 2010

http://genecube.med.yale.edu:8080/GPHMM/

*MixHMM*: version released on the 15th of April, 2010

http://genecube.med.yale.edu:8080/MixHMM/

*OncoSNP*: version 1.1 (released on 31st of May, 2011)

https://sites.google.com/site/oncosnp/

Updated *GAP*: version released on the September, 2011

http://bioinfo-out.curie.fr/projects/snp_gap/

**Parameterization**

*OncoSNP* was run with default parameters except for the expected LRR values, which were adjusted to match those of the generation, and the HMM states, limited to a copy number 5 also to match the limit in the synthetic data. Subsampling was set to 10 and 15 for the hybrid and cell-line samples, respectively.

The expected LRR values were also changed in *MixHMM* for the same reason.

In *GenoCNA*, method iterations were set to a limit of 100, instead of the default 200, as we saw that convergence was not achieved past that point.

*GAP* and *GPHMM* were run with default parameters. The parameters for the LOH region definition in the updated version of *GAP* were set to match those in the old version so that they would be the same.

*ASCAT* was run with default parameters except for those platform-specific needed to infer the genotype, given that a parameter set was not included for neither the "array type" of the synthetic samples nor for the array of the hybrid and cell-line samples.

**PFBs and GC model**

The corresponding files with PFBs and GC contents of the synthetic SNPs were generated together with the synthetic samples. We observed that results for methods that use PFBs tended to be better when the input PFB values were not those that corresponded to the generated data. These results, instead of the results with actual PFB values, are shown in the paper. GC content values were set to a constant dummy value, acknowledging the absence of GC bias in the synthetic data.

For the analyses over cell-line samples, the PFBs and GC content values of the corresponding real SNPs were used. PFBs correspond to the Caucasian population, as defined in the PFB file of the PennCNV package.
